# Evidence of structural segmentation of the Uttarakhand Himalaya and its implications for earthquake hazard

**DOI:** 10.1038/s41598-023-29432-z

**Published:** 2023-02-06

**Authors:** Prantik Mandal, Raju Prathigadapa, D. Srinivas, Satish Saha, Gokul Saha

**Affiliations:** 1 CSIR-National Geophysical Research Institute, Uppal Road, Hyderabad, India; 2 IISER, Pune, Maharashtra India

**Keywords:** Natural hazards, Solid Earth sciences

## Abstract

The earthquake hazard associated with the Main Himalayan Thrust (MHT) is a critical issue for India and its neighbouring countries in the north. We used data from a dense seismic network in Uttarakhand, India, to model the lateral variations in the depths of MHT (2–6% drop in V_s_ at 12–21 km depths), Moho (a sharp increase in V_s_ (by ~ 0.5–0.7 km/s) at 39–50 km depths) and lithosphere (a marked decrease in V_s_(~ 1–3%) at 136–178 km depths), across the Himalayan collisional front. Our joint inversion of radial PRFs and group velocity dispersion data of Rayleigh waves detects three NNE trending transverse lithospheric blocks segmenting the lithosphere in Uttarakhand Himalaya, which spatially correlate well with the northward extension of the Delhi -Haridwar Indian basement ridge, an inferred tectonic boundary and great boundary fault, respectively. Our radial receiver function imaging detects highly deformed and segmented crustal and lithospheric structures associated with three mapped transverse lithospheric blocks, suggesting a reduction in rupture lengths of future earthquakes, thereby, reducing earthquake hazards in Uttarakhand.

## Introduction

Numerous fractures, faults and folds trending normal and oblique to the trend of the Himalayan collisional zone have been recognized, which have also been found to be parallel to the great faults mapped in the basement of the Ganga basin and the South Indian block^1,2^. This observation has led to the suggestion that these sets of faults in the Himalayas and Peninsular India are possibly genetically related^1–4^. The signatures of many sub-surface hidden ridges in the basement of the Ganga basin (correlating with many peninsular orogenic trends) have been recognized in the Himalayas, which are also observed to be transverse to the main trend of the Himalayan collisional zone^2^.

The spatial distribution of these transverse faults or ridges and rupture areas of different moderate to great Himalayan earthquakes along the 2900 km long Himalayan frontal arc reveals that the Kumaon—Garhwal Himalayas in the central part has been segmented by at least three transverse structural features namely, the Delhi-Haridwar Ridge, Moradabad Fault and Great boundary fault^1–4^ (see Supplementary Fig. S1). This could be one of the reasons for the occurrences of only moderate to large size earthquakes (e.g. 1803 M_w_7.8 Garhwal, 1991 M_w_6.6 Uttarkashi and 1999 M_w_6.4 Chamoli) in this part of the Himalayas. However, the Kashmir, Nepal, and Assam Himalayas have experienced less segmentation by transverse structural features, which might have resulted in the occurrences of great Himalayan earthquakes of M_w_ ≥ 8 in these parts of the Himalayas. Thus, based on this observation it could be suggested that segmentation of the decollement surface at the Himalayan collisional boundary could be considered as a crucial parameter for assessing the likely magnitude of a major/great earthquake^2^. This structural segmentation in the Himalayas could be related to the crustal/lithospheric structure and pre-existing tectonic fabric of the underthrusting plate. Modelling of seismological and GPS data has already suggested segmentation of the Indian lithosphere along the arc^2,3,5^, which has also been evidenced by analysis of topography and Bouguer gravity anomaly data^4^. Several geophysical studies have been carried out to delineate along with arc variations in the crustal structure, geometry of MHT and angle of subduction^6–11^. Further, major transverse ridges (see Supplementary Fig. S1; e.g. Delhi-Haridwar ridge (DHR), the Faizabad ridge (FR), and the Monghyr-Saharsa ridge (MSR)) and faults (e,g, great boundary fault (GBF), Moradabad fault (MF) etc.) inherited within the underthrusting Indian plate have shown to play a key role in controlling evolution and seismicity of the Himalaya^1,2,12–14^. In 2018, Li and Song^9^ suggested shallow angle subduction in the western and eastern Himalayas compared to the central Himalayas while Dal Zilo et al.^11^ suggested shallow angle subduction for the central Himalaya as compared to western and eastern Himalayan blocks. These studies also suggested tearing the Indian lithosphere into four blocks. Dal Zilo et al.^11^ have also proposed that the inter-seismic coupling at the MHT becomes weaker in the regions coinciding with basement ridges. Thus, these ridges could act as stress barriers to stop the propagation of earthquake rupture^4^. Arc parallel topography and gravity have also been used to study the segmentation of the Indian lithosphere by these transverse ridges, which revealed a new segmentation due to the northward extension of the great boundary fault (GBF) resulting in the generation of smaller strike-slip events along the Kali river near Dharchula^15^. Therefore, the mapping of segmentation of the Indian lithosphere along the Himalayan frontal arc will be very crucial to assess the earthquake hazard associated with the different parts of the Himalayan frontal arc^16^.

The Himalayan frontal arc has been generating moderate to great size earthquakes since the initial collision between the Indian and Eurasian plates at 55 Ma^17^. During the past 1000 years, at least four M8 earthquakes occurred on the shallow portion of the megathrust boundary, with the maximum size of M_w_8.6 associated with the Assam earthquake of 1950^3^. The last large Himalayan earthquake with M_w_7.8 occurred (at 15 km depth) on 25 April 2015 in the Nepal Himalaya^18^. Modelling of geological, seismological and magneto-telluric data has mapped a north dipping shallow low velocity and conductive layer at 10–25 km depths in the Uttarakhand Himalaya on which most of the moderate to great earthquakes (e.g. the 1803 M_w_7.8 earthquake, the 1991 Uttarkashi, M_w_6.8 and the 1999 Chamoli, M_w_6.4 earthquakes) have occurred^19–21^. This mid-crustal layer is known as the seismically active main Himalayan thrust (MHT)^17^. Modelling of GPS data shows that the accumulated strain energy due to the ongoing Himalayan convergence is getting accommodated along the MHT, and is released from time to time through moderate to great earthquake occurrence^17,22^. At mid-crustal depth, the other north dipping major Himalaya thrusts (like MCT, MBT and MFT) merge with the MHT^12,23^. Seismic velocity tomograms for the rupture zone of the 2015 Nepal earthquake of M_w_7.8 have shown the MHT as a low-velocity layer at 15–30 km depths^24^. Since the MHT is formed due to the continent–continent collision of the Himalayan and Eurasian plates, thus, its geometry would vary in different parts of the 2500 km long Himalayan frontal arc^17^. The variation in the geometry of the MHT has also been observed in terms of flat and ramp structure of the MHT in different parts of the 2500 km long Himalayan frontal arc^17,19,25–30^.

Several investigations^30–37^ have found significant crustal thickness variation across the Himalayan collisional zone. In the Kumaon-Garhwal Himalaya, Moho depths vary from 35 to 55 km^5,30,31^. However, Moho depths range from 40 to 70 km in Nepal and Tibet Himalaya^32^ while they vary between 35 and 50 km in the north-eastern Himalaya^30^. Seismic imaging of the western Nepal^33^ reveals that the Moho geometry deepens from 40 km beneath the lesser Himalaya to 58 km beneath the Higher Himalaya. Modelling of gravity data^34^ has modelled a crustal thickening (68–84 km) in west Tibet. Through S-RF imaging, Xu et. al.^35^ have modelled thicknesses of lithosphere along the L1 profile (extending from (30^o^N, 81^o^E) to (36^o^N, 83^o^E)) suggesting 120–160 km in the lesser Himalaya to 200–220 km in the Higher Himalaya. Tilmann and Ni^34^ have imaged lithospheric thicknesses at 100–400 km below the INDEPTH profile in Tibet. Zhao et al.^6^ have modelled the LAB depths of the Indian lithosphere through SRF imaging that range from ~ 120–200 km in western and central Tibet, and ∼160–220 km in eastern Tibet. Below the Tian Shan belt, a much thinner lithosphere has been modelled through S-RF imaging ~ 120–170 km^35^ and ~ 90–120 km^36^, with higher temperatures (~ 1390 °C at 150 km depth)^37^. Thus, the predicted crustal and lithospheric thicknesses in different sections of the Himalayas vary greatly, requiring a better dataset from a close digital seismic network.

**Figure 1:**
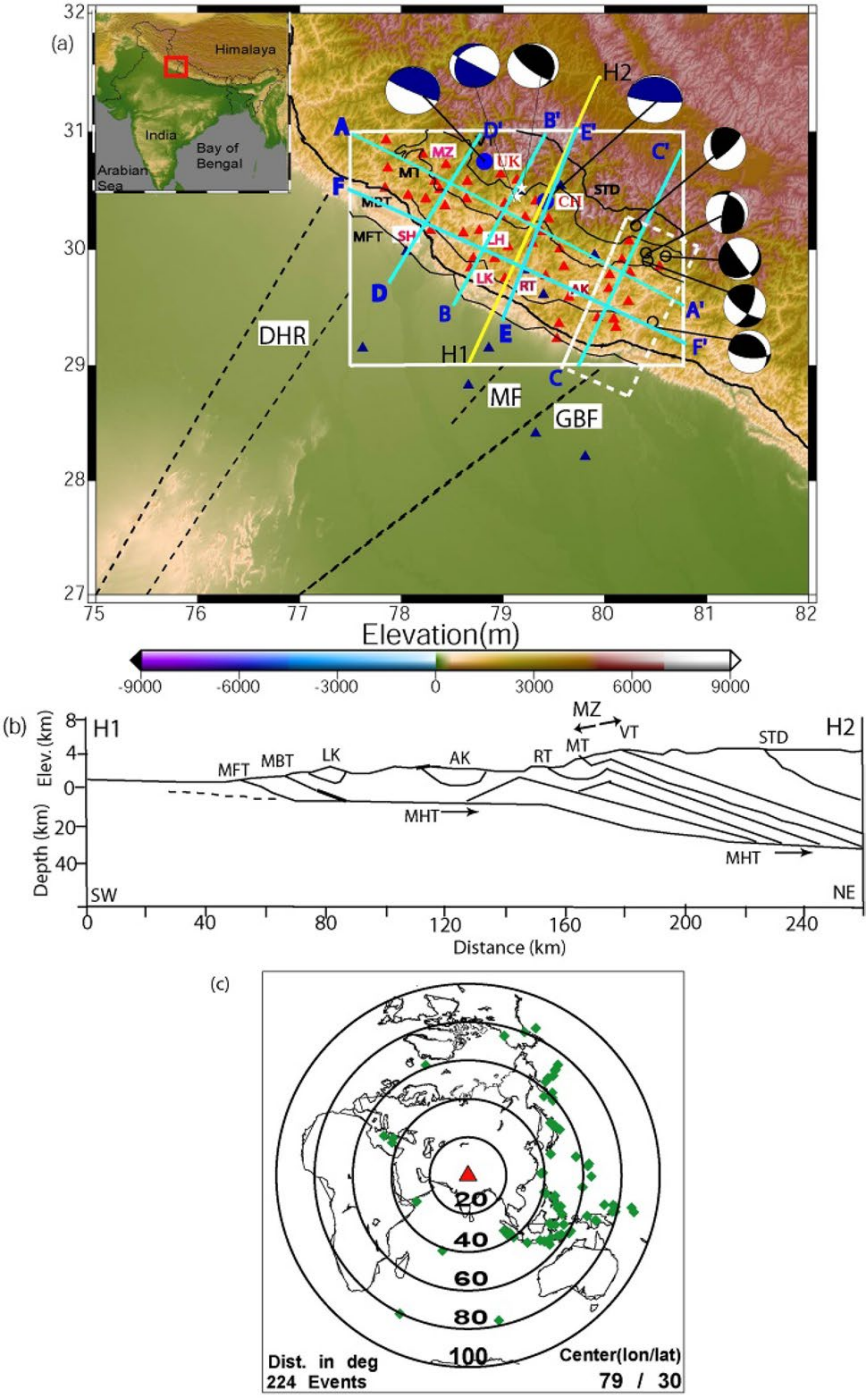
(**a**) Station location map of the Kumaon—Garhwal (KG) Himalayan region. Filled red triangles mark the location of broadband stations while filled dark blue triangles mark the locations broadband stations, which did not provide any data for our study. Two large filled blue circles mark the epicentral locations of the 1991 Uttarkashi and 1999 Chamoli earthquakes. The solid black line represents major faults. MT: Munsiari Thrust; VT: Vaikrita Thrust; MBT: Main Boundary Thrust; MFT: Main Frontal Thrust; RT: Ramgarh Thrust; MHT: Main Himalayan Thrust. SH, LH, AK, LK and MZ mark Siwalik Himalaya, Lesser Himalaya, Almore klippe, Lansdown Klippe and MCT zone, respectively. Dotted black lines mark the inferred northeast-ward extension of Delhi-Haridwar ridge (DHR), Mordabad fault (MF) and Grean Boundary Fault (GBF), respectively. Light blue lines mark the locations of six profiles (AA’, BB’, CC’, DD’, EE’ and FF’) along which 2-D images using CCP stacking are being generated. Figure 2a is generated using the Generic Mapping Tool (GMT) software version 6^62^ (https://doi.org/10.1029/2019GC008515). Existing focal mechanisms of Uttarakhand earthquakes are also shown. A white dotted box shows the region with strike-slip mechanisms (Hajra et al., 2021). The elevation data used in generating GMT plot is obtained from the open source Digital Elevation Model (DEM) (https://asterweb.jpl.nasa.gov/gdem.asp), (**b**) Tectonic depth cross-Sect. 1 across the NE-SW H1H2 profile, whose location is shown by yellow line in Fig. 2(**a**) and (**c**) Epicentral plot of 200 teleseismic events, whose broadband data from the Uttarakhand network, are used for our P-receiver function study. A red triangle and green diamond symbols mark the center of our network (Lat. 79°, Long. 30°) and epicenters of selected teleseismic events.

CSIR-National Geophysical Research Institute (NGRI), Hyderabad, launched a dense digital seismic network in October 2017 to investigate earthquake generation in the Uttarakhand Himalaya (Fig. 1a). We used P-RFs and Rayleigh wave fundamental mode group velocity dispersion data to examine lateral changes in MHT, Moho, and lithosphere-asthenosphere (LAB) depths in the Uttarakhand Himalaya. This research presents inverted crustal shear velocity models at 45 sites, which offer the 3-D spatial distribution of MHT, Moho, and LAB depths in the Uttarakhand Himalayan region.

## Geology of the area

The Himalayan geology is defined by four major geological provinces: Siwalik Himalaya (SH), Lesser Himalaya (LH), Higher Himalaya (HH), and Indo-Tsangpo suture zone (ITSZ), while the region's tectonics is defined by four north dipping thrust fault systems: the Himalayan frontal thrust (MFT), main Boundary thrust (MBT), main Central thrust (MCT), and South Tibetan detachment (STD) (see Supplementary Fig. S1). The MFT separates the SH from the Indo-Gangetic plain. The SH is largely made up of Siwalicks from the middle Miocene to the late Pleistocene^38^. MBT defines the southern boundary of LH, while MCT limits the northern boundary of LH (Fig. 1a; see Supplementary Fig. S1). The MCT separates the HH from the LH, while the STD separates the HH from the ITSZ. Granitic, gneissic, and schistose high-grade metamorphic rocks of central crystalline complex of Higher Garhwal Himalaya are found between MCT and STD^39^, while Muth granite, Nilgiri limestone, Kanchan Shale, and Ophiolites are found between STD and ITSZ^40^. North of the ITSZ, the Indian plate subducts. The occurrence of the upper Cretaceous Dras Island complex north of the ITSZ implies the presence of an Island arc and Indian plate subduction^1^. The MCT zone is defined as the area between the Munsiari thrust (MT) and the Vaikrita thrust (VT). All of these major thrusts connect to a north-dipping low angle plane at mid-crustal depths known as the main Himalayan thrust (MHT) (Fig. 1b), where the most strain energy generated by the India plate's northward convergence accumulates. This stored strain energy is periodically released as the occurrence of earthquakes of various sizes continues. Furthermore, the Himalayan frontal arc has been segmented by many inferred NNE-extensions of transverse basement ridges in the Ganga basin (such as the Delhi-Haridwar ridge (HDR), the Faizabad ridge (FZR), and the Munger-Saharsa ridge)^2–4,12^. Several studies^2–4,12^ have concluded that the DHR has been extended below the Higher Himalaya.

## Methodology

### Earthquake data and computation of P-receiver functions

**Figure 2:**
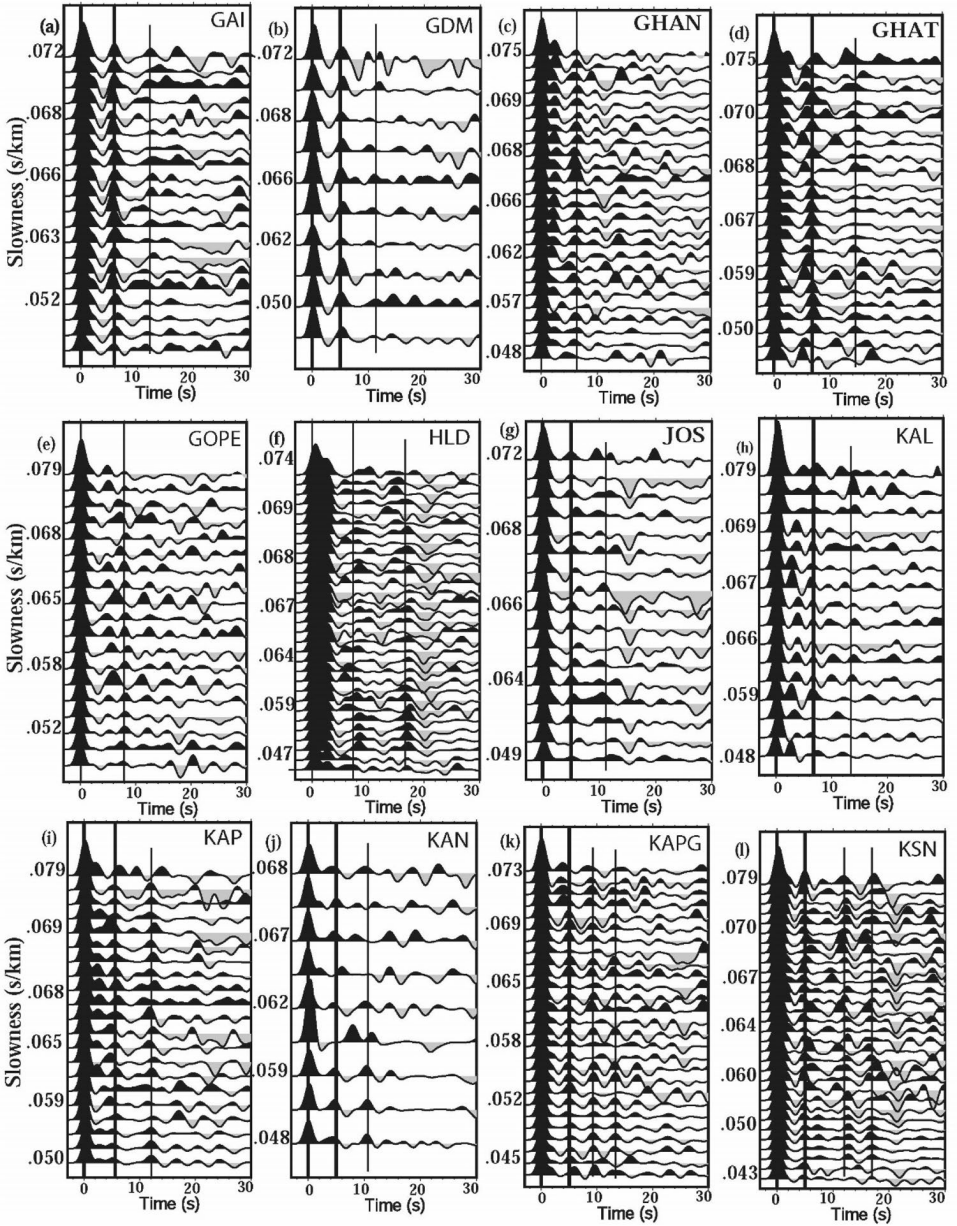
Plots of individual RFs (for a Gaussian width factor, a = 2.0) as a function of the horizontal slowness after distance moveout correction for the *Ps* phase to a reference distance of 67^◦^ and slowness 6.4 s deg^−1^, for 15 broadband sites in the KG Himalaya, (**a**) GAI, (**b**) GDM, (**c**) GHAN, (**d**) GHAT, (**e**) GOPE, (**f**) HLD, (**g**) JOS, (**h**) KAL, (**i**) KAP, (**j**) KAN, (**k**) KAPG, and (**l**) KSN. The PRFs at each station show strong azimuthal variation. The arrivals of direct P and conversions from the Moho (P_ms_) and crustal multiples (i.e. PpPs and PpPs + PpSs) are marked by solid black lines. However, for some stations, where the arrival of PpPs + PpSs crustal multiple is weak, are not marked. Here, we have used IASP91 velocity model as the reference model for predictions.

The CSIR-National Geophysical Research Institute, Hyderabad, has been operating a dense broadband digital seismic network of 56 three-component seismographs (Fig. 1a) in the Uttarakhand Himalaya since 2017, with an average interstation spacing of 19 ± 8 km. Here, we utilized above network’s waveform data of 224 good teleseismic events of m_b_ ≥ 5.5 (with back azimuth between 38° and 309°, and ray parameters ranging from 0.047 to 0.077 s/km; as shown in Fig. 1c), to compute radial and transverse P-receiver functions (PRFs) through the Ligorria and Ammon^41^’s time domain deconvolution with 200 iterations. Three frequency bands corresponding to Gaussian width factors, a = 1.0 (f < 0.5 Hz), a = 1.5 (f < 0.75 Hz) and a = 2.0 (f < 1.0 Hz), are considered to compute PRFs with an objective to detect gradational changes in seismic velocities^41,42^. Here, we used those PRFs for which time-domain deconvolutions reproduced ≥ 90% of the signal energy on the radial component (when convolved back with the vertical trace). Further, we selected only those radial PRFs whose transverse PRFs show minimum amplitudes. Following the above criteria, we obtain a total of 1700 good individual radial PRFs (with minimum amplitude on the transverse PRFs), using 3000 three-component waveforms from 45 out of 56 broadband stations. These individual radial P-RFs at 45 broadband stations suggest at least three prominent detectable phases corresponding to conversions from the mid-crustal MHT, Moho and LAB representing a velocity increase across the Moho and a velocity decrease across the MHT and LAB (Fig. 2a–l; see Supplementary Figs. S2a, b, S3a–o, S4a–j). Individual radial P-RFs from 45 broadband stations also show arrivals of two crustal multiple conversions PpPs and (PpPs + PpSs) (Fig. 2; see Supplementary Figs. S3, S4). We also plot stacked radial and transverse PRFs at 24 stations out of 45 stations, showing clear P-to-S conversions from MHT, Moho and LAB (see supplementary Figs. S5, S6, S7). The clear negative phase associated with the low-velocity MHT arrive at 1.2–3.0 s after the arrival of direct P. The sharp and positive conversions from the Moho (P_ms_) arrive at 3.6–7.0 s after the arrival of direct P, on the individual radial PRFs at all the forty-five stations (Figs. 2a–l; kindly also see supplementary Figs. S3, S4, S5, S6, S7). They also show a clear negative arrival at 16–22 s after the arrival of direct P, associated with the P-to-S conversion (P_ls_) from the LAB (Figs. 2a–l; kindly also see Supplementary Figs. S3, S4, S5, S6, S7). Note that the migrations of stacked PRFs with depth along three NE-SW profiles suggest that Moho depth varies from ~ 40 to 52 while LAB ranges from ~ 130–200 km (see Supplementary Figs. S8a–d).

### Joint inversion of PRFs and Surface wave group velocity dispersion

**Figure 3:**
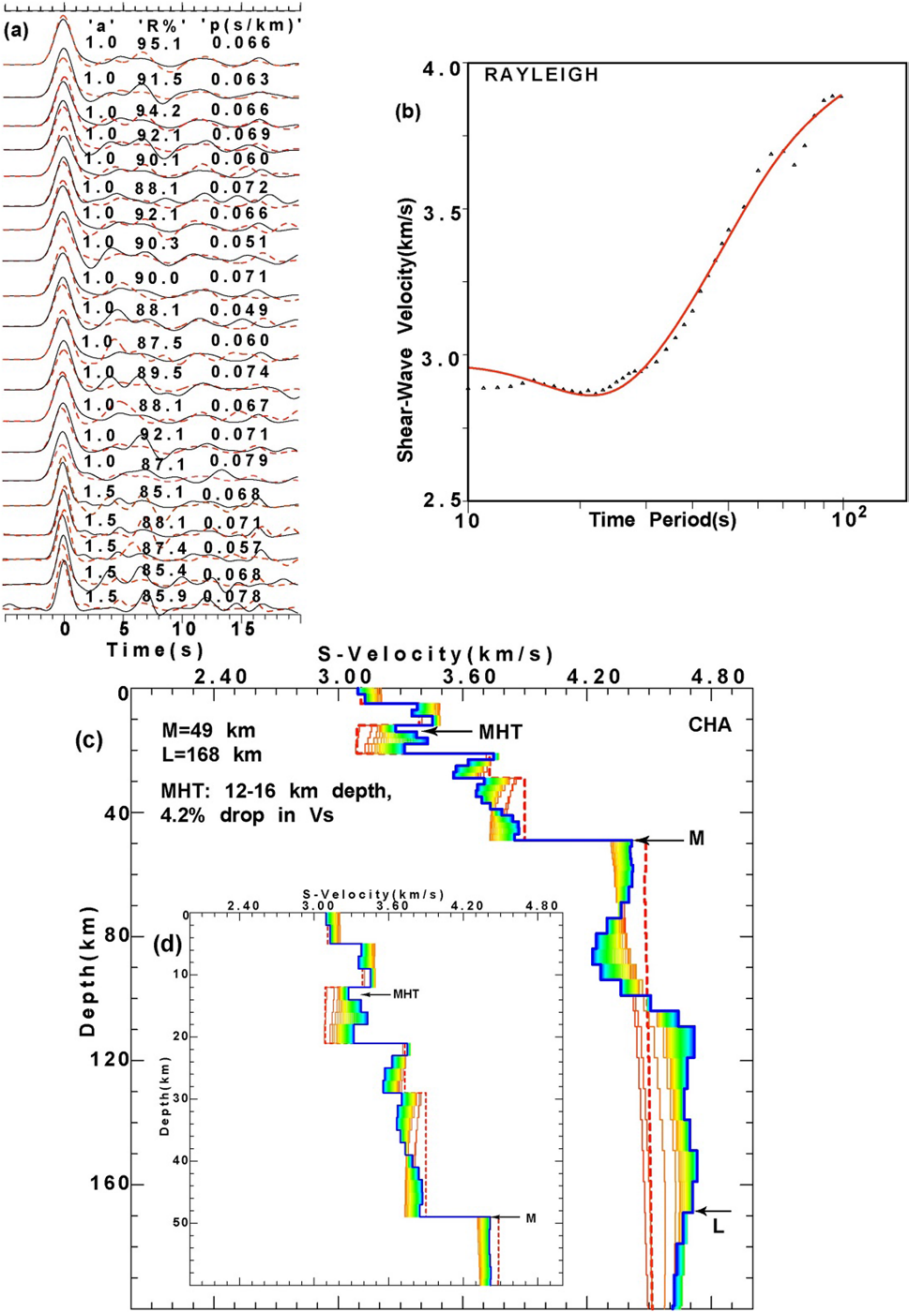
Results of joint inversion of P-RFs and fundamental mode Rayleigh wave group velocity dispersion data at CHA station, (**a**) showing good agreement between observed (black line) and inverted (red line) radial RFs with a = 1.0 and 1.5, for different horizontal slowness (S, in s/km). Here, “a” and “R%” represent Gaussian width factor (used for estimating RF) and agreement (in %) between observed and inverted RFs, respectively. (**b**) Correlation between observed and inverted dispersion curve of Rayleigh waves. (**c**) Inverted shear velocity models showing Moho (M) and LAB (L) depth estimates. Different colors represent different Versus models used for the joint inversion. The initial shear velocity model is shown by a thick red dotted line, while the final shear velocity model is shown by a thick blue line, and (**d**) zoomed portion of figure (**c**) showing only crustal Versus model. Furthermore, MHT, M, and L mark the thickness of the Main Himalayan Thrust in km, Moho depths in km and lithospheric thickness in km, respectively.

**Figure 4:**
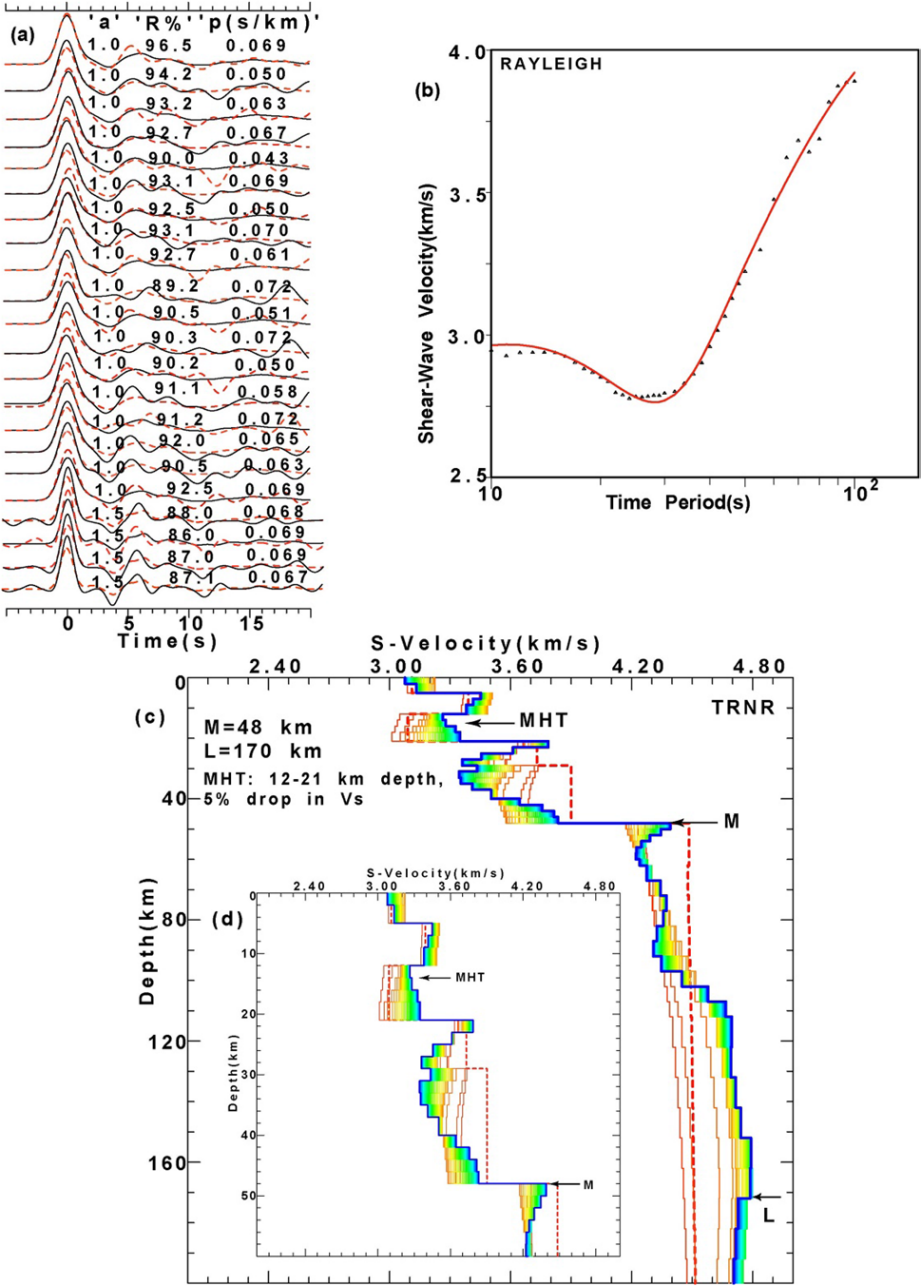
Results of joint inversion of P-RFs and fundamental mode Rayleigh wave group velocity dispersion data at TRNR station, (**a**) showing good agreement between observed (black line) and inverted (red line) radial RFs with a = 1.0 and 1.5, for different horizontal slowness (S, in s/km). Here, “a” and “R%” represent Gaussian width factor (used for estimating RF) and agreement (in %) between observed and inverted RFs, respectively. (**b**) Correlation between observed and inverted dispersion curve of Rayleigh waves. (**c**) Inverted shear velocity models showing Moho (M) and LAB (L) depth estimates. Different colors represent different Versus models used for the joint inversion. The initial shear velocity model is shown by a thick red dotted line, while the final shear velocity model is shown by a thick blue line, and (**d**) zoomed portion of figure (**c**) showing only crustal Versus model. Furthermore, MHT, M, and L mark the thickness of the Main Himalayan Thrust in km, Moho depths in km and lithospheric thickness in km, respectively.

In this study, a minimum of 21 and a maximum of 42 individual radial PRFs at 45 different stations are used to estimate the MHT thickness, reduction in vs at MHT, Moho and lithosphere-asthenosphere boundary (LAB) depths, through the joint inversion^43^. At each station, we use radial PRFs estimated for different back azimuths and fundamental mode group velocity dispersion data of Rayleigh waves, for our joint inversion study^43^. Here, we utilize the dispersion measurements done by Saha et al.^44^ for their Rayleigh wave tomographic study of Peninsular India. Saha et al.^44^ have used a total of 21,600 paths by combining noise and earthquake group velocities for their Rayleigh wave group velocity measurements utilizing 3-component broadband digital waveforms of 417 events from 209 seismic stations. Tomographic images were constructed by them at 10–100 s periods using path averaged group velocity measurements at 1◦ × 1◦ grid cells (see Supplementary Figs. S9, S10). We extracted surface wave group velocity dispersion data at 10–100 s periods for each of our station from the above discussed tomograms of Saha et al.^44^ (Figs. 3, 4; also see Supplementary Figs. S11–S53). The use of Rayleigh wave group velocity dispersion data up to 100 s has enabled us to delineate the V_s_-structure up to a depth of 200 km^45^. Here, the crustal part of initial 1-D velocity models are constrained by the final Vp and Vs models as obtained from the simultaneous inversion of P- and S- arrival times^46^ while the deeper part of initial velocity models are constrained by the IASP91 model of Kennett and Engdahl^47^. The Moho depths for the initial models are varying from 28.3 km (at BTL) to 52.9 km (at PAUR)^5^.

**Figure 5:**
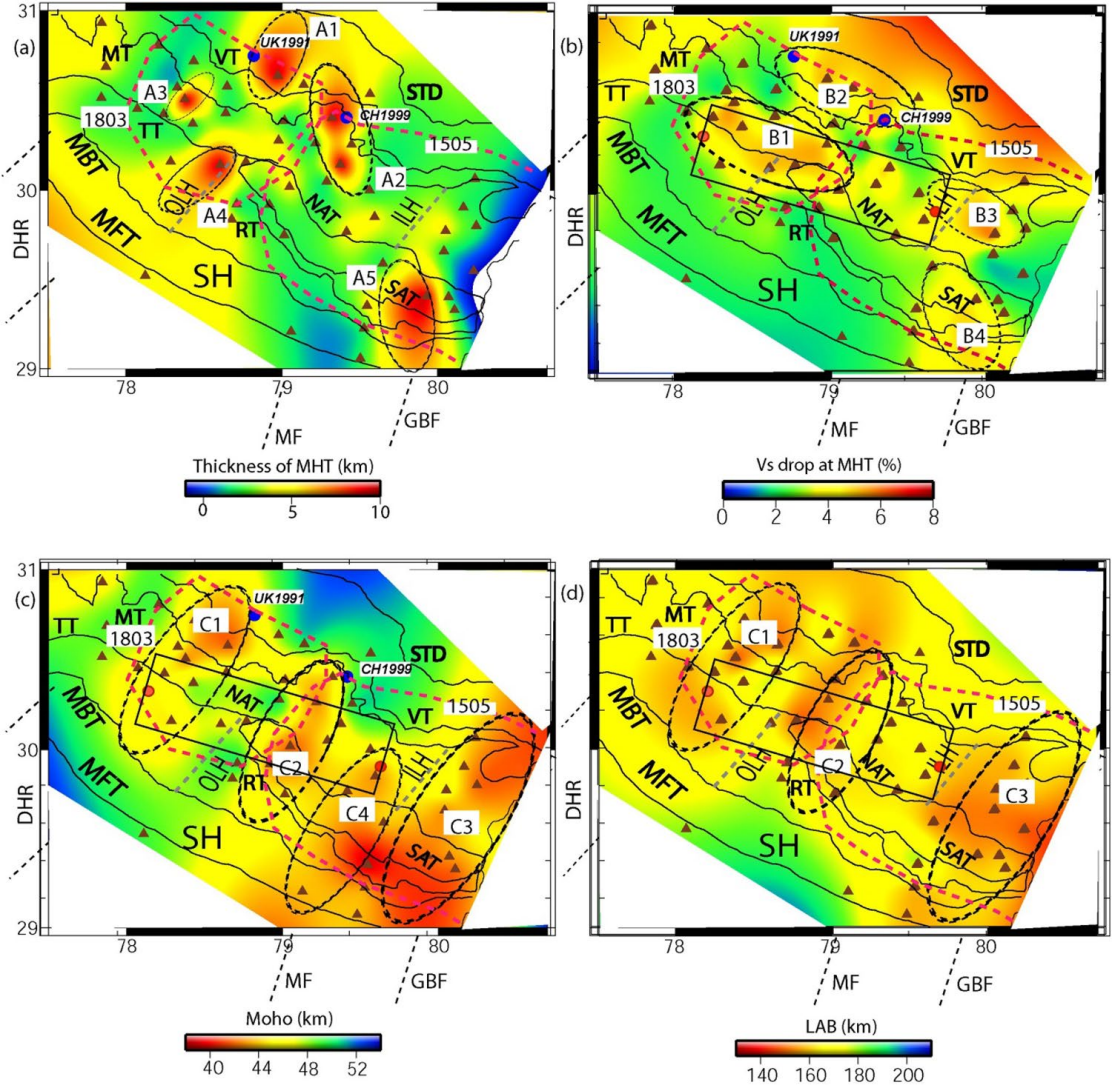
Contour plots of modelled (**a**) MHT thickness (in km), (**b**) Drop in V_s_ at the MHT (in %), (**c**) Moho depths (in km) and (**d**) lithospheric thickness (in km), through the joint inversion of PRFs and surface wave dispersion data, with major geological formations in the Singhbhum Craton. The locations of the 1991 Uttarkashi (M_w_6.6) and 1999 Chamoli (M_w_6.4) earthquakes are shown by large filled blue circles in Fig. 5(**a**–**c**). DHR marks the inferred NE extension of the Delhi-Haridwar basement ridge while MF represents a NE striking inferred Moradabad fault^23^. And, GBF marks the NE extension of the Great Boundary Fault separating the ADFB from the VB further south (Fig. 1). Black dotted elliptical zones (A_1_, A_2_, A_3_, and A_4_) in Fig. 5(**a**) represent zones of large MHT thicknesses while they (B_1_, B_2_, B_3_, and B_4_) mark the zones of Versus drop at MHT in Fig. 5(**b**). But, black dotted elliptical zones (C_1_, C_2_, C_3_, C_4_) mark the mapped NE trending crustal transverse features as shown in Fig.  5(**c**) while they (C_1_, C_2_, and C_3_) represent mapped NE trending lithospheric transverse features as shown in Fig.  5(**d**). The inferred rupture zones of the 1803 and 1505 paleo earthquakes are marked by dotted pink lines (after Bilham (2019). Major thrusts (shown by black lines): VT: Vaikrita Thrust; MT: Munsiari Thrust; TT: Ton Thrust; RT: Ramgarh Thrust; NAT: North Almora Thrust; SAT: South Almora Thrust; MBT: Main Boundary Thrust; MFT: Main Frontal Thrust. SH marks the Siwalik Himalaya. ILH marks the inner lesser Himalaya while OLH marks the outer lesser Himalaya. A black rectangular area in Fig.  5(**b**–**d**) represents the location of the conductor as inferred by numerical modelling of the magnetometer array data of the UK Himalaya^53^.

Here, we use the joint96 seismological code of Herrmann^48^ that utilizes the Julia et al.^43^’s joint inversion technique to delineate 1-D shear velocity structure. This inversion scheme iteratively inverts for the S-wave velocity and then updates the P-velocity using the V_p_/V_s_ ratio of the initial model. Subsequently, the relation of Berteusen^49^ is used to compute density values for new V_p_ values. Note that joint inversions are repeated each iteration, to find joint models that fit the receiver functions and group velocity dispersion data of Rayleigh waves. The inversion scheme provided a stable solution for different stations after 40 iterations, with damping of 1.0 and an influence parameter of 0.3 (see supplementary Table S1). The weight (or priority) given to PRF and Dispersion data for the joint inversion is determined by the Influence parameter. For the joint inversion, influence parameter = 0.3 means that 70% of the weights is given to the PRF and 30% to the dispersion data. The joint inversion is stopped only after obtaining the best fit Vs model showing good correlation (≥ 85%) between the all available observed and inverted P-RFs (over the available range of horizontal slowness and back-azimuths at one station) and fundamental mode group velocity dispersion data of Rayleigh waves. The same procedure of joint inversion is performed to estimate the best-fit 1-D shear velocity model for all 45 broadband stations (down to a depth of 200 km) in the Uttarakhand Himalayan region (Figs. 3, 4; also see Supplementary Figs. S11–S53). The estimated MHT thickness, reduction in V_s_ at MHT, Moho depths (M), and lithospheric thicknesses (L) are listed in supplementary Table S1, and, their contour plots are shown in Fig. 5a–d.

### Common conversion point (CCP) stacking of PRFs

**Figure 6:**
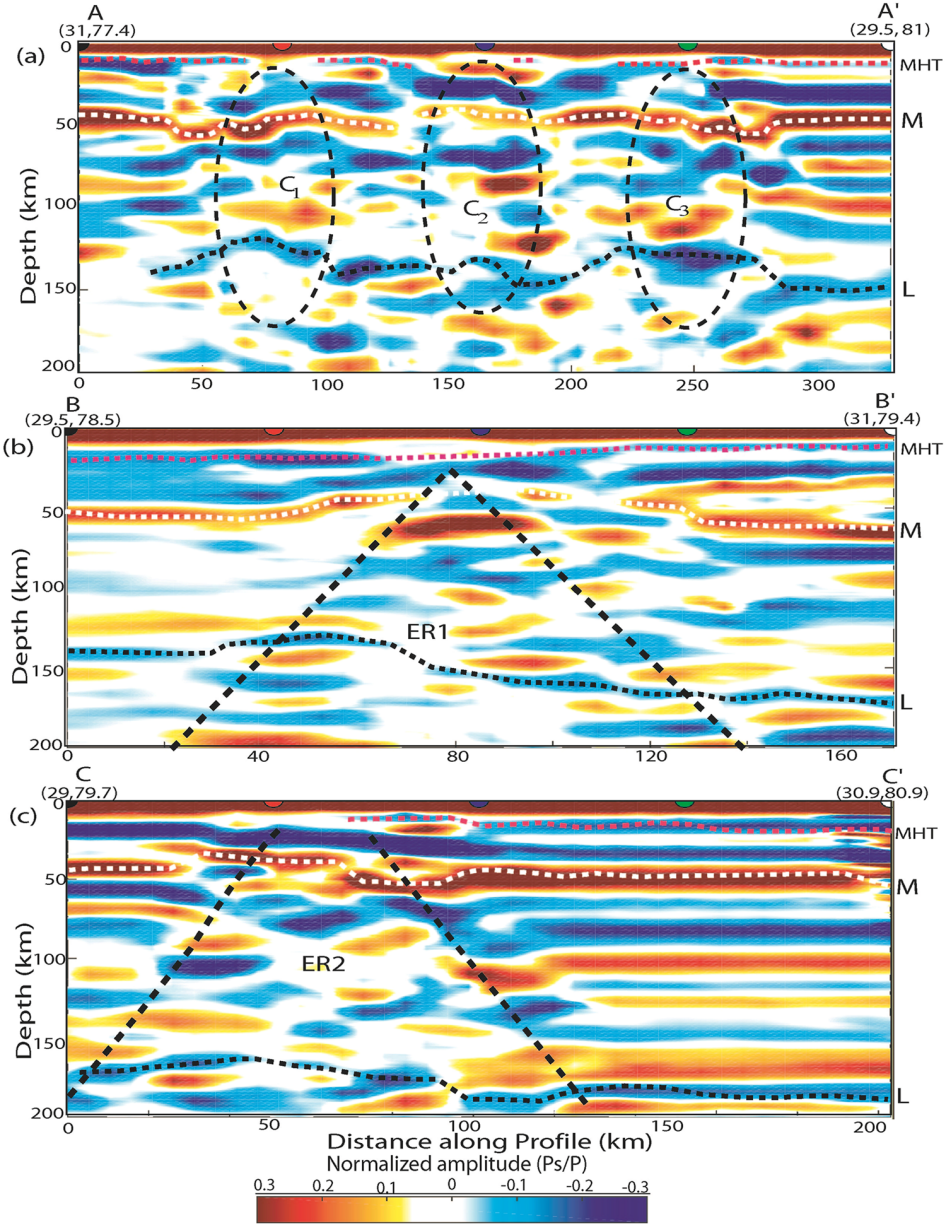
CCP stacking of PRFs using 1-D IASP91 velocity model along three profiles, whose locations are shown in Fig. 1a. Dotted lines show north dipping Main Himalayan Thrust (MHT), Moho and Lithosphere-Asthenosphere Boundary (LAB) below the Lesser Himalaya in the Uttarakhand Himalaya (**a**) AA’ profile, (**b**) BB’ profile and (**c**) CC’ profile. The locations of these profiles are shown in Fig. 1a. Black dotted elliptical areas mark the locations of mapped three lithospheric structures (C_1_, C_2_, and C_3_), which are spatially correlating with the northward extension of the DHR, inferred tectonic boundary and GBF. Thick pink, white and black dotted lines mark the north dipping MHT, Moho and lithosphere. While inverted triangle shape areas (marked by very thick black dotted lines) represent the highly deformed and segmented crustal and lithospheric areas (ER1 and ER2) within the northward extension of the inferred tectonic boundary and GBF, respectively, which are transverse to the Himalayan collisional boundary.

**Figure 7:**
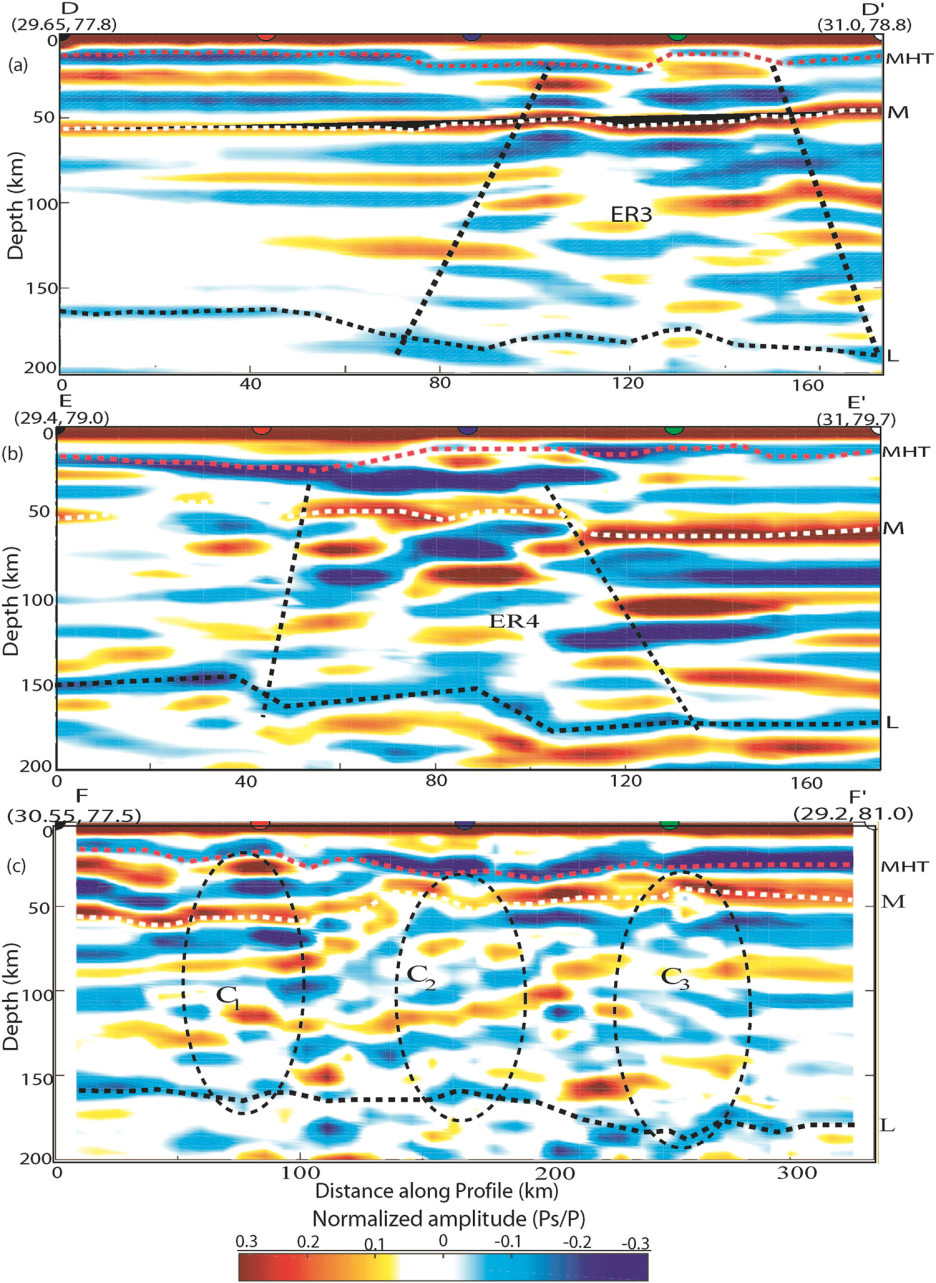
Same as Fig. 6a–c along profiles (**a**) DD’, (**b**) EE’ and (**c**) FF’. The locations of these profiles are shown in Fig. 1a. Thick pink, white and black dotted lines mark the north dipping MHT, Moho and lithosphere. Black dotted elliptical areas mark the locations of mapped three lithospheric structures (C_1_, C_2_, and C_3_), which are spatially correlating with the northward extension of the DHR, inferred tectonic boundary and GBF. While inverted triangle shape areas (marked by very thick black dotted lines) represent the highly deformed and segmented crustal and lithospheric area (ER3 and ER4) within the northward extension of the Delhi-Haridwar basement ridge and the inferred tectonic boundary coinciding with the intersecting area of the ruptures zones of the 1505 and 1803 paleo earthquakes.

Here, we use the Funclab software^50^ to perform CCP imaging of radial PRFs, using Dueker and Sheehan^51^’s methodology to coherently stack p-to-s phase conversions for generating a 2-D image of impedance contrast at depth. The details of CCP imaging methodology have been discussed in Cladwell et al.^28^ and the manual of Funclab^50^. Here, we used the 1-D IASP91 velocity model for the CCP imaging^47^. We have performed CCP imaging along six profiles (two along and four across the Himalayan collisional front), whose locations are shown in Fig. 1a. The results of CCP stacking of radial PRFs are shown in Figs. 6a–c and 7a–c, which show lateral variations in the modelled MHT, Moho and LAB below the Uttarakhand Himalaya.

We compute PRFs using three frequency bands corresponding to Gaussian width factors, a = 1.0 (f < 0.5 Hz), a = 1.5 (f < 0.75 Hz) and a = 2.0 (f < 1.0 Hz). Thus, the wavelegth would be 8, 5.3, 4 km for f = 0.5, 0.75 and 1.0, respectively, for an average crustal shear velocity of 4.0 km/s. Thus, the calculated PRFs with a = 1.0, 1.5 and 2.0 can resolve layers with thickness of 4, 2.65 and 2 km, respecively. The nominal vertical resolution at the Moho could be 4 km for the 1-D PRFs and 1.7 km for 2-D CCP stack^28^. While the horizontal resolution depends on the frensel zone. The fresnel zone width for Ps at 40 km is ~ 40 km. The station spacing for our stations varies from 10 to 20 km. So our station spacing of ~ 20 km could yield 50% overlap at 40 km depth, which is found to be sufficient to image the Moho well^30^. At 10 km depth, the frensel zone width is 20 km, which could be imaged with 50% overlap for our stations with a 10 km spacing. But, our station spacing could not provide better resolution for images at depths less than 10 km.

## Results and discussions

We estimate MHT, Moho, and lithosphere thicknesses at 45 three-component broadband stations across the Uttarakhand Himalaya by jointly inverting radial PRFs and fundamental mode group velocity dispersion data of Rayleigh waves (Figs. 3, 4; see Supplementary Figs. S11–S53). MHT thicknesses and Vs at MHT depths are estimated to be 2–9 km and 2–6%, respectively (Figs. 3, 4; see Supplementary Figs. S11–S53 and Table S1). Moho depths range from 39 kms (at BTL) to 50 km (at SAT, RPG, RANS, and GHAT), and lithospheric thicknesses range from 136 km (at DHRL) to 176 km (at GHAN) (Figs. 3, 4; see Supplementary Figs. S11–S53 and Table S1). Our modelled Moho and LAB depth estimates accord well with our depth migration predictions from the stacked PRFs (see Supplementary Fig. S8a–d). At Moho depths, V_s_ increases from 0.5 km/s (at ALM and PATI stations) to 0.7 km/s (at TEH station), whereas V_s_ decreases from 1% (at DHRL, KAL, LAN, and MUS stations) to 3% (at GAI and POKH stations) (Figs. 3, 4; see Supplementary Figs. S11–S53 and Table S1). With a thickness of 39 km, BTL in the Kumaon Himalaya has the thinnest crust. The Garhwal Himalaya has the thickest crust of 50 km at SAT, RPG, RANS, and GHAT (Figs. 3, 4; see Supplementary Figs. S11–S53 and Table S1), which falls in a zone of thicker crust (Fig. 5c), with an average crustal thickness of 47.3 km (i.e. mean of the modelled Moho depths at GAI, GHAN, GOPE, KAPG, NBR, PAUR (Figs. 3, 4, see Supplementary Figs. S11–S53 and Table S1). Other seismological and magnetotelluric studies in the Himalayas of Uttarakhand^5,20,21,30^ support our Moho depth estimations.

A recent magneto-telluric (MT) study along a NNW-SSE profile north of Delhi mapped the Indian plate crust below a sedimentary layer as a collage of resistive and conductive blocks separated by nearly vertical contacts, coinciding with the Delhi-Haridwar ridge (DHR) and great boundary fault (GBF) traces. Several other vertical contacts have been mapped below DHR and GBF, which may indicate faults or transverse geological structures. The Garhwal Himalaya may be underlain by the DHR, the western part of the Aravalli-Delhi fold belt (ADFB)^2,11,13,52^. An earlier MT study along the Roorkee profile suggested a highly resistive crustal block coinciding with the DHR^53^ that correlates well with the mapped R1 transverse structure with marked thinning of mafic crust^5^, which might be representing a rigid, mafic resistive crustal block. Another MT study^21^ along a profile 70 km east of the Roorkee profile suggested a conductive crustal block, which spatially matches a marked thickening of felsic (relatively conductive^52^) crust between R_1_ and R_2_ transverse resistive mafic blocks as delineated by Mandal et al.^5^. These MT studies also show that the lesser Himalaya has resistive and conductive blocks^53,54^. Manglik et al.^54^ concluded from their modelling that the ADFB's resistive and conductive blocks may continue beneath the Kumaun-Garhwal Himalaya (KGH). Thus, one can further infer that the Indian plate crust in the lesser Himalaya is composed of a college of resistive and conductive blocks separated by vertical contacts coinciding with NNW-SSE trending extension of major faults like DHR/MDF, GBF, etc., resulting in a spatially highly heterogeneous crust below the MHT^54^. The H–K stacking of radial PRFs^5^ found three NS-to-NNE trending transverse structures (viz., R_1_, R_2_ and R_3_) beneath the KGH with significant upwarping of mafic crust (V_p_/V_s_ (~ 1.85–2.13)). The R_1_, R_2_ and R_3_ coincide with the NNE trending traces of DHR, MF and GBF, respectively^5^.

Our joint inversion of PRFs and group velocity dispersion data of Rayleigh waves reveals four significant NNE trending transverse structures, C_1_, C_2_, C_3_, and C_4_, with marked mafic crustal thinning on the study region's western, middle, and eastern ends (Fig. 5c). C_2_ is strongly related to an inferred tectonic boundary that intersects the rupture zones of the 1803 M_w_7.8 Garhwal and 1505 M_w_8.2 central Himalaya paleo-earthquakes^17^. Our mapped transverse structures C_1_, C_3_, and C_4_, spatially correlate well with the NNE extension of the DHR, MF, and GBF, which may be the same as the R_1_, R_2_, and R_3_ transverse structures as delineated by the H–K stacking study of radial PRFs^5^. These mapped inherited transverse structures must have intruded into the Indian crust, which has since submerged beneath the Eurasian plate's upper crust. As a result, these data could be interpreted as a lower crustal intrusion layer near the crust's base (perhaps induced by the northward extension of DHR/MF/GBF)^5^. C_1_ and C_2_ bend northward below the MCT zone and the region close north of it, most likely indicating the subducted Indian plate from the north (Fig. 5c; also see Supplementary Fig. S54b). Furthermore, our modelling identifies a zone of greatest change in Moho depths (marked by a red elliptical zone in Supplementary Fig. S54b). Within the MHT, this zone is likewise found to have a high pore-fluid pressure (marked by elliptical areas shown by black dotted lines in Fig. 5a, b). Surprisingly, the vertical downward projections of the hypocenters of the Uttarkashi (UK) earthquake in 1991 and the Chamoli (CH) earthquake in 1999 are both located within this zone. Thus, the 1991 M_w_6.6 UK and 1999 M_w_6.4 CH MHT thrust earthquakes could be linked to increased pore-fluid pressure due to the presence of metamorphic fluids at mid-crustal depths^5,33,46^. Furthermore, the major variations in Moho depths (Fig. 5c; also see Supplementary Fig. S54b) and the persistent northward under-thrusting of the Indian plate at a rate of 14 mm/year^17,22^ could have played a key role in generating the two moderate-sized earthquakes outlined above. Our 3-D structural model identifies a definite north-dipping surface with significant thinning of the Indian crust and lithosphere, which we believe is the subducted Indian plate.

Lateral changes in modelled lithosphere-asthenosphere boundary (LAB) depths reveal three NNE trending transverse zones of marked lithospheric thinning (e.g., C_1_, C_2_, and C_3_), which spatially match well with our mapped three transverse zones of marked crustal thinning (e.g., C_1_, C_2_, and C_3_) (Fig. 5c; also see Supplementary Fig. S54b). Modelled lithospheric thicknesses, on the other hand, do not map the C_4_ transverse zone, as illustrated in Fig. 5c by the contour plot of our Moho depth estimates. As a result, the sub-crustal depths are not reached by our mapped C_4_ transverse structure. Thus, we can conclude that our three mapped transverse zones (C_1_, C_2_, and C_3_) extend from the bottom of the MHT to the LAB depth, dividing the underthrusted Indian lithosphere into three different NNE trending transverse zones beneath the Uttarakhand Himalaya (Fig. 5c, d; also see Supplementary Fig. S54b, c). The mapped C_1_ and C_3_ zones exhibit a high spatial association with the northward extensions of DHR and GBF (Fig. 5c, d; also see Supplementary Fig. S54b, c). Prior to the Himalayan collision at 50 Ma^2,4,12^, the DHR and GBF could have been transmitted down from multiple important tectonic events in Peninsular India. With the Indian lithosphere, these inherited transverse characteristics could have been subducted beneath the Eurasian plate^2^. As earlier studies^4,6,7,9–11,25^ have suggested, these mapping imprints of the ancient fabric of Indian plate crust/lithosphere structures may have played a crucial role in establishing along-arc differences in deformation, seismicity, and mountain building processes.

It is worth noting that the strongest factor in the Himalaya^55^ is the northward compression generated by the collision of the Indian and Eurasian plates. The Indian plate, on the other hand, has been rotated anti-clockwise^56^. The dominant compressional stress regime may have changed to an extensional stress regime as a result of the above-mentioned rotation, resulting in fracturing of the Indian shield during the collision with the Eurasian plate, allowing upward intrusion of magma from the asthenosphere^52^, leading to the formation of a number of transverse faults and fracture zones dissecting the lesser Himalayas rather extensively, as suggested by Valdiya^57^. These transverse faults/fractures zones have been widely connected with the subsurface ridges/faults of the north Indian plains, including the Delhi-Haridwar ridge (DHR), the Moradabad fault (MF), and the Great Boundary Fault (GBF)^4,57–59^. Furthermore, from computational modelling of magnetometer array data from the Uttarakhand Himalaya^52,58^, a 45 km-wide conductive ridge (between the DHR and the MF in the Lesser Himalayas) has been estimated. This mapped conductive zone spatially correlates well with the high seismicity zone^57^, implying that earthquake-related stresses reactivated subsurface structure beneath the region, which could have facilitated upward movement of mafic material into the crust and can be seen in Fig. 5c,d as C_1_, C_2_, C_3_, and C_4_.

Our modelling also suggests that the Himalayan arc segment between the DHR and the FR, traditionally assumed to be a single block with a shallow subduction angle^11^, has most likely been segmented into four tectonic blocks: C_1_, C_2_, C_3_, and the western Nepal block. C_1_ and C_3_ have a good spatial association with the DHR and GBF's northward extension (see Supplementary Fig. S1), whereas C_2_ has a strong spatial correlation with the intersection zone of the rupture zones of the 1803 M_w_7.8 Garhwal and 1505 M_w_8.2 central Himalaya paleo-earthquakes^17^ (Fig. 5a–d). This junction zone may have been a tectonic boundary, meaning that it served as a tectonic barrier, limiting the propagation of the rupture fronts of the 1505 and 1803 earthquakes. The DHR basement ridge extends beneath the Himalayas and is associated to the Kaurik-Chango rift^2^, whereas the GBF is thought to persist beneath the Dharchula region, resulting in strike-slip earthquakes^59^. It is worth noting that Arora and Mahashabde^52^ discovered a 45-km-wide conductive ridge (between Chamba and Chutukia, depicted by a black rectangular area in Fig. 5b–d) rising from asthenospheric depths to a depth of 15 km, which corresponds well with our modelled transverse features C_1_, C_2_, and C_4_ between the DHR and the MF. While mapped C_3_ transverse structure model spatially corresponds well with the GBF, which could also represent similar transverse structure^5^. Based on the concurrence of the conductor and upper crustal seismic zones in the region, Arora and Mahashabde^52^ postulated that pressures associated with under-thrusting of the Indian plate may produce crack in the Indian shield, leading in the upflow of asthenospheric material in the form of ridge. This model supports the idea that our transverse features could reach all the way to the lithosphere-asthenosphere boundary. As a result, our transverse structural features (C_1_, C_2_, and C_3_) can continue down to the LAB below the Uttarakhand Himalaya (Fig. 5c, d). We hypothesise that these transverse structural features were carried down through the Indian lithosphere by important tectonic occurrences in Peninsular India. These features, as well as the Indian plate, were later subducted to the north (see Supplementary Fig. S54b, c).

Other collisional mountain belts around the world, such as the Appalachians and Alps, have also demonstrated cross/transverse segmentation^60^. The segmentation of the MHT in the western Himalayas has already been described using low temperature thermochronometry data^61^ and mapped geometry of duplex/ramp structures^19,62,63^. It has been claimed that the Indian craton's basement structures impacted the placement and development of the Himalayan cross structures^1,2^. However, the depth extents of these cross formations within the Himalaya are unknown. The presence of strike-slip earthquakes at depths of 50–60 km demonstrates the depth extent of the Himalaya's cross or transverse structures^59^. This type of cross construction was found to limit the lateral propagation of the 2015 Nepal earthquake rupture^64^. Now, whether slip on these cross faults affects the MHT hazard or not is a significant question that must be answered^65^. Slips on cross faults connected to the subducting plate, on the other hand, have been detected in the Juan de Fuca, NW Sumatra, and Andeas subduction zones^66–68^. As a result, 3-D mapping of these cross faults is crucial for a better understanding of earthquake hazard in the Himalayas and other collisional mountain belts worldwide.

Our CCP stacking of radial PRFs along six profiles (two along (AA' and FF') and four across the Himalayan collisional boundary (BB', CC', DD', and EE')) clearly revealed the lateral changes in MHT, Moho, and LAB depths throughout the Uttarakhand Himalaya (Figs. 6a–c, 7a–c). The two-dimensional images along the WSW-ENE trending AA' and FF' profiles clearly delineate three elliptical regions with marked crustal and lithospheric thinning, which correlate well with our mapped three transverse features (C_1_, C_2_, and C_3_), implying variations in MHT, Moho, and LAB depths along the Himalayan collisional front's strike. Two-dimensional PRF-images along four profiles over the Himalayan front (BB', CC', DD', and EE') have clearly revealed north dipping MHT, Moho, and lithosphere, exposing footprints of Indian plate subduction beneath the Eurasian plate. MHT depths vary across strikes along the BB', CC', DD', and EE' profiles, indicating a northward rise in MHT, Moho, and LAB depths, supporting the notion of Indian plate subduction (Figs. 6b, c, 7a, c). Images along the BB', CC', DD', and EE' profiles clearly indicate four triangular zones of highly deformed and segmented crustal and lithospheric structures beneath the MHT (Figs. 6b, c, 7a, c), which could be generated by the presence of four transverse structures C_1_, C_2_, C_3_, and C_4_. The prevalence of strike-slip crustal earthquakes along the BB' and CC' profiles (Fig. 1a) supports the hypothesis of transverse structures like GBF expanding northward and the presence of a tectonic boundary. Our 2-D PRF images along the DD' and EE' profiles (Fig. 7a–c) demonstrate a minor bend of MHT near the hypocentral locations of the M_w_6.6 Uttarkashi and M_w_6.4 Chamoli events in 1991 and 1999, respectively (Fig. 1a). Existing focal mechanisms and moment tensor solutions for small to moderate earthquakes^57^ show that strike slip mechanisms along the GBF (i.e., C_3_) and the inferred tectonic boundary (i.e., C_2_) are dominant (Fig. 1a). These findings support the presence of transverse Indian basement ridges/faults beneath the MHT in Uttarakhand's Lesser Himalaya. The presence of detectable zones of highly deformed and segmented crustal and lithospheric structures beneath the MHT associated with transverse structures in the lesser Himalaya suggests that these zones do not have unbroken long fault lengths capable of generating large/great earthquakes in the Uttarakhand Himalaya in the future.

As illustrated in Fig. 1a, we additionally model the MHT and Moho in greater detail by CCP stacking radial PRFs at 0–100 km depth along three NE-SW profiles, DD', BB', and EE' (see Supplementary Fig. S55a–c). Our modelling finds a definite shallow NE dipping plane (with large negative Ps/P amplitudes) between 10 and 15 km deep across all three profiles due to the presence of metamorphic fluids, which could represent the low-velocity Main Himalayan Thrust (MHT) (shown by white dotted line in the Supplementary Fig. S55a–c). In all three profiles, we also find a NE dipping crust-mantle boundary (with large positive Ps/P amplitudes) at depths of 35–55 km (shown by white dotted line in the Supplementary Fig. S55a–c). Seismic imaging of western Nepal^33^ shows a mid-crustal low-velocity zone at 15 km deep, which has been linked to fluids ejected by rocks descending in the footwall of the Main Himalayan Thrust. Most notably, our modelling detects a double Moho structure beneath the epicentral zone of the 1999 Chamoli earthquake along the BB' profile, which, together with the Indian plate's continued northward convergence, may have provided the large stress concentration in the rupture zone on the MHT to generate the 1999 Chamoli earthquake, which may have been triggered by metamorphic fluids within the MHT (see Supplementary Fig. S55b). It should be noted that Li et al.^69^ modelled a similar Moho doublet structure in Lhasa, Tibet, using CCP stacking of radial PRFs, and that a similar Moho doublet structure has also been modelled in terms of velocity jumps in the 1-D velocity model along the HIMNET (i.e. the Himalaya Nepal Tibet Seismic Experiment) beneath the high Himalaya and Tibet^70^. A double Moho structure beneath Tibet^71,72^ has also been discovered using wide angle data modelling. As a result, we may conclude that our MHT, Moho, and LAB depth estimates from three separate investigations (joint inversion of PRFs and Rayleigh wave group velocity dispersion, CCP stacking of radial PRFs, and migration of stacked PRFs with depth) are very close, implying that our estimates are robust.

## Conclusions

Segmenting the seismically active Himalayan continent–continent collisional zone reduces rupture lengths for future earthquakes, reducing seismic hazard. Thus, earthquake magnitude will depend on Himalayan arc rupture length. In addition to convergence rates, lateral differences in crustal/lithospheric structure along the Himalayan arc will be needed to anticipate future destructive earthquakes. We estimate the lateral changes in MHT thickness, Moho depths, and lithospheric thicknesses at 45 three-component broadband stations in the Uttarakhand Himalaya by jointly inverting radial PRFs and Rayleigh wave fundamental mode group velocity dispersion data. Our modelling identified three NNE-trending transverse zones or cross structures, C_1_, C_2_, and C_3_, with considerable crustal and lithospheric weakening. Thickening crust and lithosphere divide these transverse zones. C_1_ and C_3_ strongly correlate with the northward extensions of the DHR and GBF, respectively, whereas C_2_ strongly correlates with the intersection zone between the rupture zones of the 1803 M_w_7.8 Garhwal and 1505 M_w_8.2 central Himalaya paleo-earthquakes. We map another NNE-trending C_4_ transverse feature that matches the MF's northward extension, which does not reach sub-crustal depths. Thus, our study suggests that genetic linkages exist between our transverse structures and Peninsular India's major basement ridges/faults' northward extension. Our CCP stacking of radial PRFs shows significantly deformed and segmented crustal and lithospheric structures (below the MHT) corresponding with the mapped transverse lithospheric features C1, C2, and C3. This supports this concept. These transverse structures have segmented crustal and lithospheric faults, reducing earthquake risk in Uttarakhand Himalaya. Our modelling shows four tectonic blocks: C1, C2, C3, and western Nepal between the epicentres of the 1803 and 1505 prehistoric events. Thus, the rupture length between the epicentral locations of the two earthquakes mentioned above has been segmented into four parts, reducing the rupture lengths available for generating future potential earthquakes in this region and lowering the likelihood of devastating earthquakes in Uttarakhand. Our findings reduce the risk of M_w_ ≥ 7.5 earthquakes in the Uttarkhand Himalaya in the future.

## Supplementary Information

Supplementary Information.

## Data Availability

Data and material for this paper have been obtained from published sources, and relevant references to the sources are provided. The link for data is mentioned below for your kind consideration: https://ngri.org.in/86578/CGcode_prf.zip**.** The raw waveforms for some selected events could also be obtained from the Director, CSIR-NGRI, Hyderabad, through e-request (director@ngri.res.in).

## Code availability

No commercial code or software has been used in the research presented in the paper. Seismological codes of Hermann^48^ for the joint inversion of radial PRFs and fundamental mode group velocity dispersion data of Rayleigh waves can be obtained from the Saint-Louis University’s website. While the Funclab software (Eagar^50^) can be obtained from the Funclab website. These softwares are available in the user domain.
