# Author Correction: Evidence of structural segmentation of the Uttarakhand Himalaya and its implications for earthquake hazard

**DOI:** 10.1038/s41598-023-30481-7

**Published:** 2023-03-02

**Authors:** Prantik Mandal, Raju Prathigadapa, D. Srinivas, Satish Saha, Gokul Saha

**Affiliations:** 1 CSIR-National Geophysical Research Institute, Uppal Road, Hyderabad, India; 2 IISER, Pune, Maharashtra India

Correction to: *Scientific Reports* 10.1038/s41598-023-29432-z, published online 06 February 2023

The original version of this Article contained an error in the Code availability section.

“No code or software has been used in the research presented in the paper. Seismological codes of Hermann^48^ for the joint inversion of radial PRFs and fundamental mode group velocity dispersion data of Rayleigh waves can be obtained from the Saint-Louis University’s website. While the Funclab software (Eagar^50^) can be obtained from the Funclab website. These softwares are available in the user domain.”

now reads:

“No commercial code or software has been used in the research presented in the paper. Seismological codes of Hermann^48^ for the joint inversion of radial PRFs and fundamental mode group velocity dispersion data of Rayleigh waves can be obtained from the Saint-Louis University’s website. While the Funclab software (Eagar^50^) can be obtained from the Funclab website. These softwares are available in the user domain.”

The original Article and accompanying Supplementary Information file have been corrected.

