# Supplementary material for: Evidence of structural segmentation of the Uttarakhand Himalaya and its implications for earthquake hazard
Source: Sci Rep. 2023 Feb 6;13:2079. doi: 10.1038/s41598-023-29432-z (PMC9902400; doi:10.1038/s41598-023-29432-z)
Supplement: Supplementary file 1 — Supplementary Information. [file 41598_2023_29432_MOESM1_ESM.pdf]

# Supplementary materials for

# Evidence of structural segmentation of the Uttarakhand Himalaya and its implications for earthquake hazard

Prantik Mandal*, Raju Prathigadapa, D. Srinivas, Satish Saha and Gokul Saha

*Corresponding author. Email: prantikmandal62@gmail.com

This material includes a description of data and algorithms. It also presents the results of the Joint inversion of PRFS and fundamental mode group velocity dispersion data of Rayleigh waves. It includes 55 figures showing radial PRFs and results of the joint inversion of radial PRFs and surface wave dispersion of Rayleigh waves. The results of the joint inversion at 45 broadband stations are listed in the Table S1.

## Data and Seismic Network

In 2017, a seismic network consisting of 56 three-component broadband seismographs was installed by CSIR-NGRI, Hyderabad, in the Uttarakhand Himalayan region (See Supplementary Fig. S1; Fig.1a). Each station is equipped with a 24-bit Reftek-130 recorder, 120s Reftek 3-component broadband sensor and a GPS clock for time tagging. This network has recorded several hundred good regional and teleseismic events during 2017-2021. Here, we utilized the dataset from the above network to jointly invert the radial PRFs and fundamental mode group velocity dispersion data of Rayleigh waves to estimate thicknesses of MHT, crust and lithosphere in the Uttarakhand Himalaya. A high resolution 24-bit Reftek-130 recorder and a Reftek broadband sensor are used at each station, with a GPS time tagging. The continuous broadband data at each station is recorded at 50 samples per second. The seismographs were located on hard rock sites for achieving higher signal to noise ratio. During February 2017 - November 2021, 200 good teleseismic earthquakes were recorded by 45 out of 56 seismographs from the above network (Figs. 1a-c). To estimate PRFs, first a wavefrom from -5 to 60 s is considered from the 3-component broaband data, which is corrected from the instrumental correction. Then, all the windowed waveforms are filtered using a high pass filter with a corner frequency of 0.03 Hz before the computation of PRFs.

For the present study, we used the above-mentioned dataset to compute radial and transverse PRFs through time domain deconvolution method of Ligorria and Ammon (1) (see supplementary Figs. S2-S7). The depth migration of PRFs has also been performed to estimate depths of the Moho and lithosphere-asthenosphere boundary (see Supplementary Fig. S8)

## Joint Inversion of radial PRFs and group velocity dispersion data

Here, we use 1700 radial P-receiver functions showing clear P-to-s conversions associated with the Moho and other crustal multiples (Fig. S2-S7), which are estimated through the time domain deconvolution (with a Gaussian width of 1.0, 1.5 and 2.0) procedure of Ligorria and Ammon (1) with 200 iterations, from 45 out of 56 broadband stations in the Uttarakhand region (Fig.1a), to conduct the joint inversion[2] study using radial P-RFs and fundamental mode group velocity dispersion data of Rayleigh waves, to estimate MHT thickness, Moho depths and lithosphere thicknesses in the Uttrakhand Himalayan region (see supplementary Table S1; Figs. 2-3, see Supplementary Figs. S11-S53). Due to the uneven azimuthal distribution of teleseismic events (Fig. 1c), reliable anisotropic structures are difficult to retrieve with this dataset, and so we invert only for isotropic crustal structure. For surface wave tomography, Saha et al.[3] have used interpolation based on the three closest neighbours, and nodes are separated at nearly constant distances from one another. This is done by constructing Delaunay triangles from the set of nodes on the sphere. Each triangle formed by the three nodes at the plane's vertices, which is almost the tangent plane to the sphere. Three-point linear interpolation is used to evaluate the model within each Delaunay triangle. Rayleigh wave dispersion data are extracted from previous tomographic study[45] that

provides group velocity data in the period of 10s to 100s at 0.5° interval. The group velocity map was generated using ambient noise and earthquake data recorded over a network of 683 board band seismic stations in India, Tibet and surrounding regions. A representative group velocity map of the region at 10s, 50s, and 100s time period is presented in the supplementary Fig.S9. Also, we provide results of a checkerboard resolution test for periods 10s and 100s in the supplementary Fig.S10. Saha et al.[3] generated resolution test with square cells of size varying from 0.5° to 2° and found 1°×1° to be the optimum. Tomographic images were constructed by them at 10-100 s periods using path averaged group velocity measurements at 1◦ × 1◦ grid cells. We extracted surface wave group velocity dispersion data at 10 -100s periods for each of our station from the above discussed tomograms of Saha et al.[3]. For joint inversion study, three different influence parameters viz., 0.3, 0.4 and 0.5 have been used to perform the joint inversion for selecting the correct influence parameter. After this examination, an influencing parameter of 0.3 is chosen for the joint inversion study. Next, at each station the joint inversion is performed for three different damping parameters, i.e., 0.4, 0.6 and 1.0, for examining the performance of each value of the influence parameter. For a damping parameter of 1, the RMS velocity model perturbation and estimated data standard deviation are found to be minimum. The inversion scheme provided a stable solution for different stations after 40 iterations, with damping of 1.0 and an influence parameter of 0.3 (see supplementary Table S1). Thus, our final model is more influenced by PRFs than the regional surface wave group velocity dispersion data, which have been constructed from the regional tomograms[3]. Here, we perform 40 iterations of inversion for different stations to obtain the final model at 45 broadband stations (Figs. 2-3; also see Supplementary Figs. S11–S53). When we ran the joint inversion, we could fit the surface waves without significantly degrading the fit to the receiver functions. The joint inversion is stopped only after obtaining the best fit Vs model showing good correlation (≥ 85%) between the all available observed

and inverted P-RFs (over the available range of horizontal slowness and back-azimuths at one station) and fundamental mode group velocity dispersion data of Rayleigh waves. The same procedure of joint inversion is performed to estimate the best-fit 1-D shear velocity model for all 45 broadband stations (down to a depth of 200 km) in the Uttarakhand Himalayan region (Figs. 2-3; also see Supplementary Figs. S11–S53). Results from the joint inversion at 45 stations are shown in Figs. 2-3 and supplementary Figs. S11-S53. The 2-D CCP images along three NE-SW profiles are shown in supplementary Figs. S54a-c. The 3-D structural map showing elevation (m), Moho depths (in km) and LAB (in km) is shown in supplementary Figs. S55a-c.

References:

1. J.P. Ligorria, C.J. Ammon, Iterative deconvolution and receiver-function estimation. *Bull. Seismol. Soc. Am.* 89(5), 1395–1400 (1999).
2. J. Julia, C.J. Ammon, R.B. Herrmann, A.M. Correig, Joint inversion of receiver function and surface-wave dispersion observations. *Geophys. J. Int.* 143, 99–112, doi 10.1046/j.1365-246x.2000.00217.x (2000).
3. G.K. Saha, K.S. Prakasam, S.S. Rai, Diversity in the peninsular Indian lithosphere revealed from ambient noise and earthquake tomography. *Phys, Earth and Planet. Interiors* 306, 106523, 1–17 (2020).

Supplementary materials of 55 figures and 1 table:

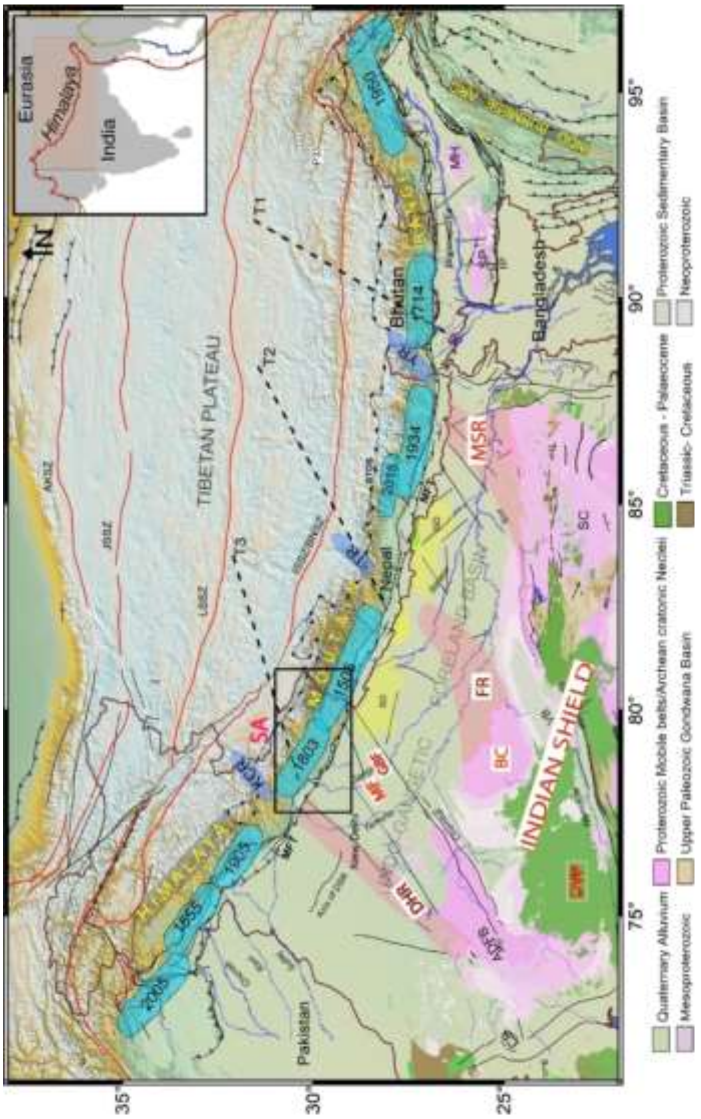


Figure S1: (a) A map showing geology and tectonics of the Himalaya, Indo-Gangetic plain and surrounding regions on the elevation map from SRTM data ([https://dwtkns.com/srtm30m/](https://dwtkns.com/srtm30m/)). This figure is generated using GIS (https://www.caliper.com/maptitude/gis_software/default.htm) and Surfer Software (https://surfer-9.software.informer.com/download/). The rupture zones of the significant and great Himalayan earthquakes along the arc are also shown by blue filled rectangular zones. T1, T2 and T3 are block boundaries marked by Li and Song[9]. Black rectangular area marks our study area. Abbreviated names shown on the map are: ADFB: Aravalli-Delhi fold belt, DVP: Deccan Volcanic Province, VB: Vindhyan basin, BC: Bundelkhand craton, SC: Singhbhum craton, CB: Cambay basin, SP: Shillong plateau, MH: Mikir hills, DHR: Delhi-Haridwar ridge, DSR: Delhi-Sargodha ridge, FR: Faizabad ridge, MSR: Monghyr-Saharsa ridge, KCR: Kaurik –Chango rift, TR: Thankola rift, YR: Yadong rift, GD: Gandak depression, SD: Sharda depression, MFT: Main frontal thrust, MBT: Main boundary thrust, MCT: Main central thrust, STDS: South Tibetan Detachment system, ITSZ: Indus-Tsangpo Suture Zone, BNSZ: Bangong Nujiang Suture Zone, LSSZ: Longmu Tso Shuanghu Suture Zone, JSSZ: Jinsha Suture Zone, AKSZ: Anyemaqen Kunlun Suture Zone, DF: Dauki Fault. Inset displays a rectangular area showing the location of the map area (Modified after Manglik et al.[4]).

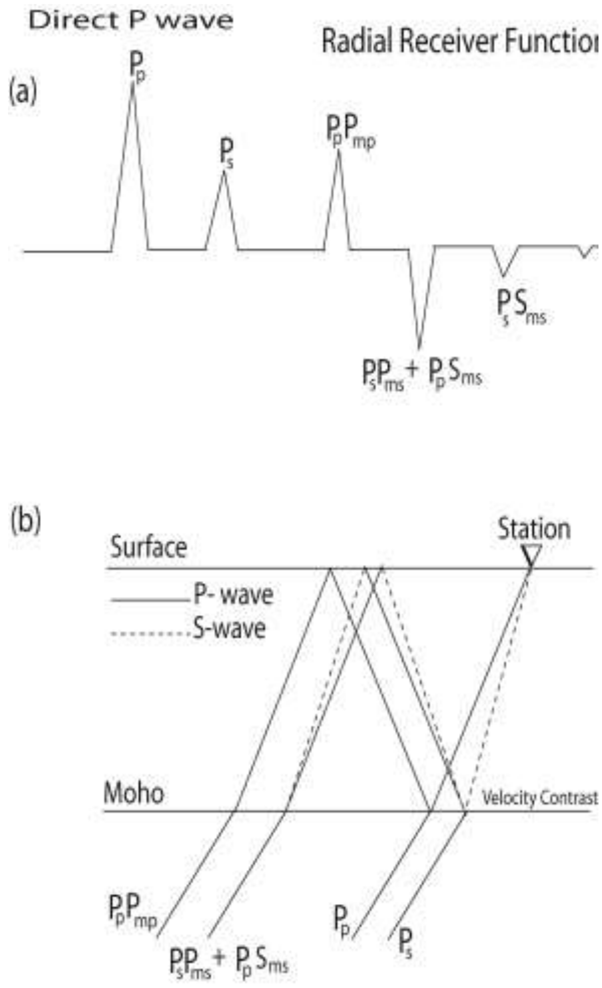


Figure S2: (a) A radial P-receiver function showing arrivals of various direct and converted phases of teleseismic P phases and (b) A plot showing raypaths of P-to-s conversions from the Moho and other crustal multiples.

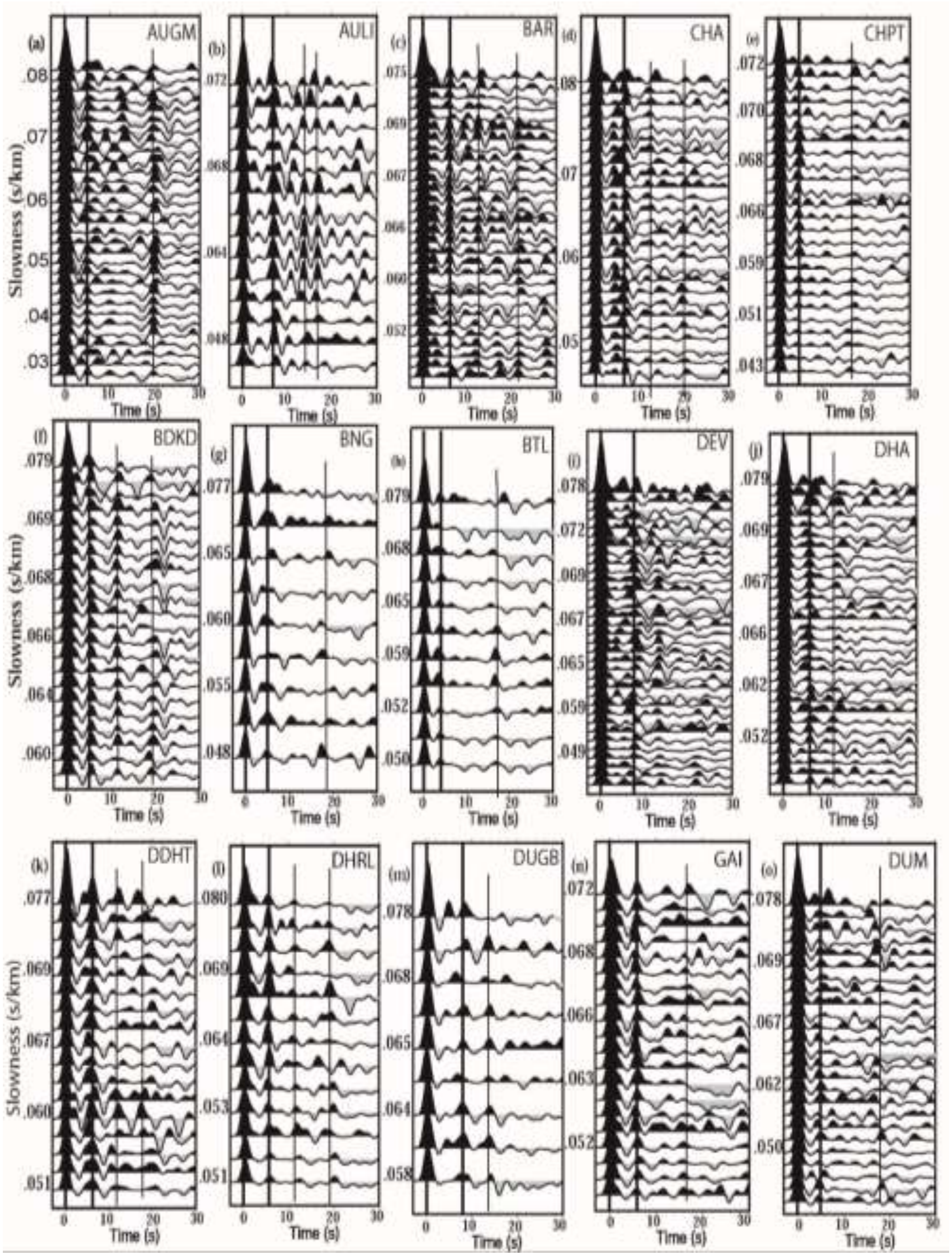


Fig S3: Plots of individual RFs as a function of the horizontal slowness after distance moveout correction for the *Ps* phase to a reference distance of 67° and slowness 6.4 s deg$^{-1}$, for 15 broadband sites in the UK Himalaya, (a) AUGM, (b) AULI, (c) BAR, (d) CHA, (e) CHPT, (f) BDKD, (g) BNG, (h) BTL, (i) DEV, (j) DHA, (k) DDHT, (l) DHRL, (m) DUGB, (n) GAI, and (o) DUM. The PRFs at each station show strong azimuthal variation. The arrivals of direct P and conversions from the Moho ($P_{ms}$) and crustal multiples (i.e. PpPs and PpPs+PpSs) are marked by solid black lines. However, for some stations, where the arrival of PpPs+PpSs crustal multiple is weak, are not marked. Here, we have used IASP91 velocity model as the reference model for predictions.

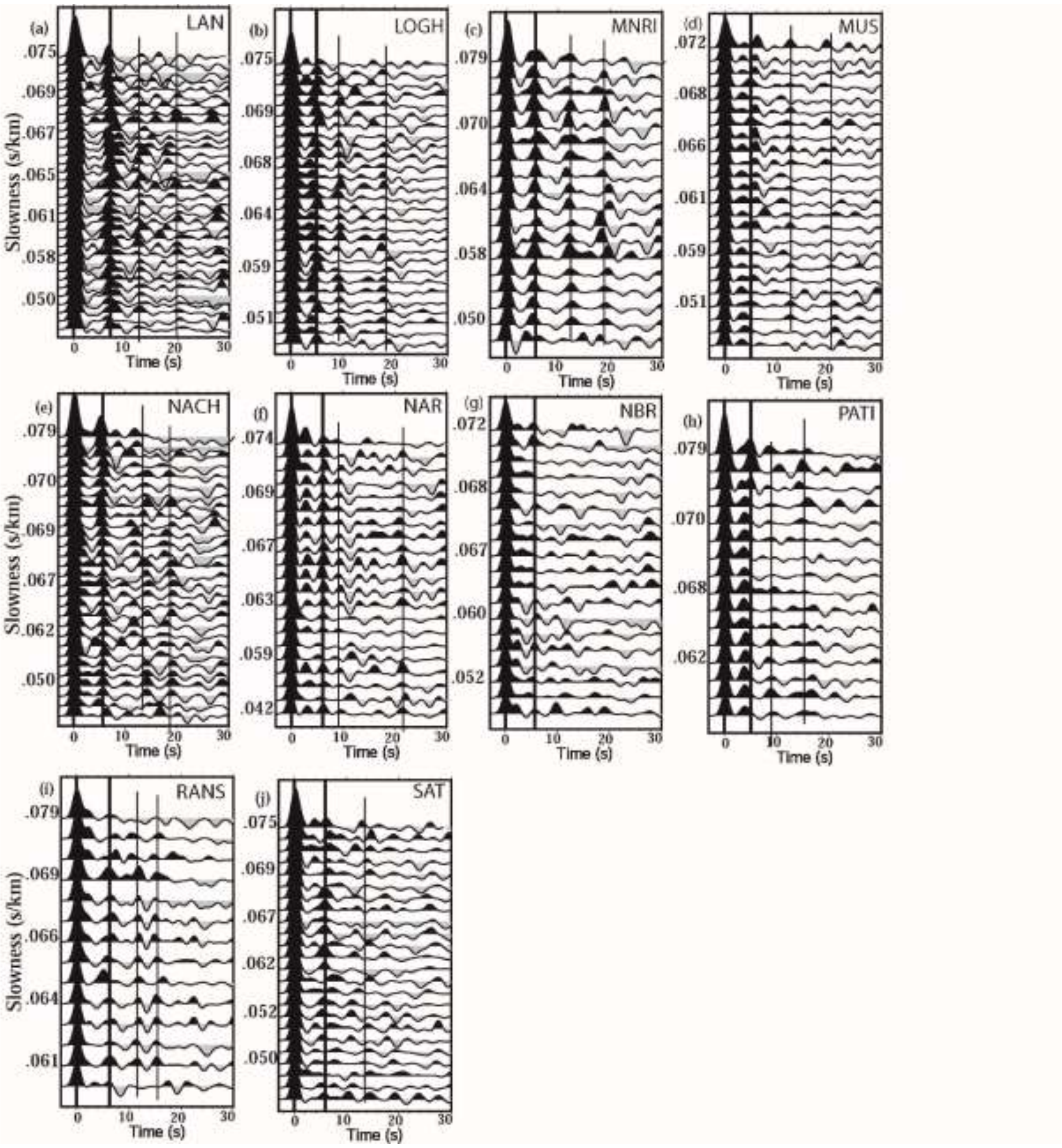


Fig S4: Plots of individual RFs as a function of the horizontal slowness after distance moveout correction for the *Ps* phase to a reference distance of 67° and slowness 6.4 s $deg^{-1}$, for 10 broadband sites in the UK Himalaya, (a) LAN, (b) LOGH, (c) MNRI, (d) MUS, (e) NACH, (f) NAR, (g) NBR, (h) PATI, (i) RANS, and (j) SAT. The PRFs at each station show strong azimuthal variation. The arrivals of direct P and conversions from the Moho ($P_{ms}$) and crustal multiples (i.e. PpPs and PpPs+PpSs) are marked by solid black lines. However, for some stations, where the arrival of PpPs+PpSs crustal multiple is weak, are not marked. Here, we have used IASP91 velocity model as the reference model for predictions.

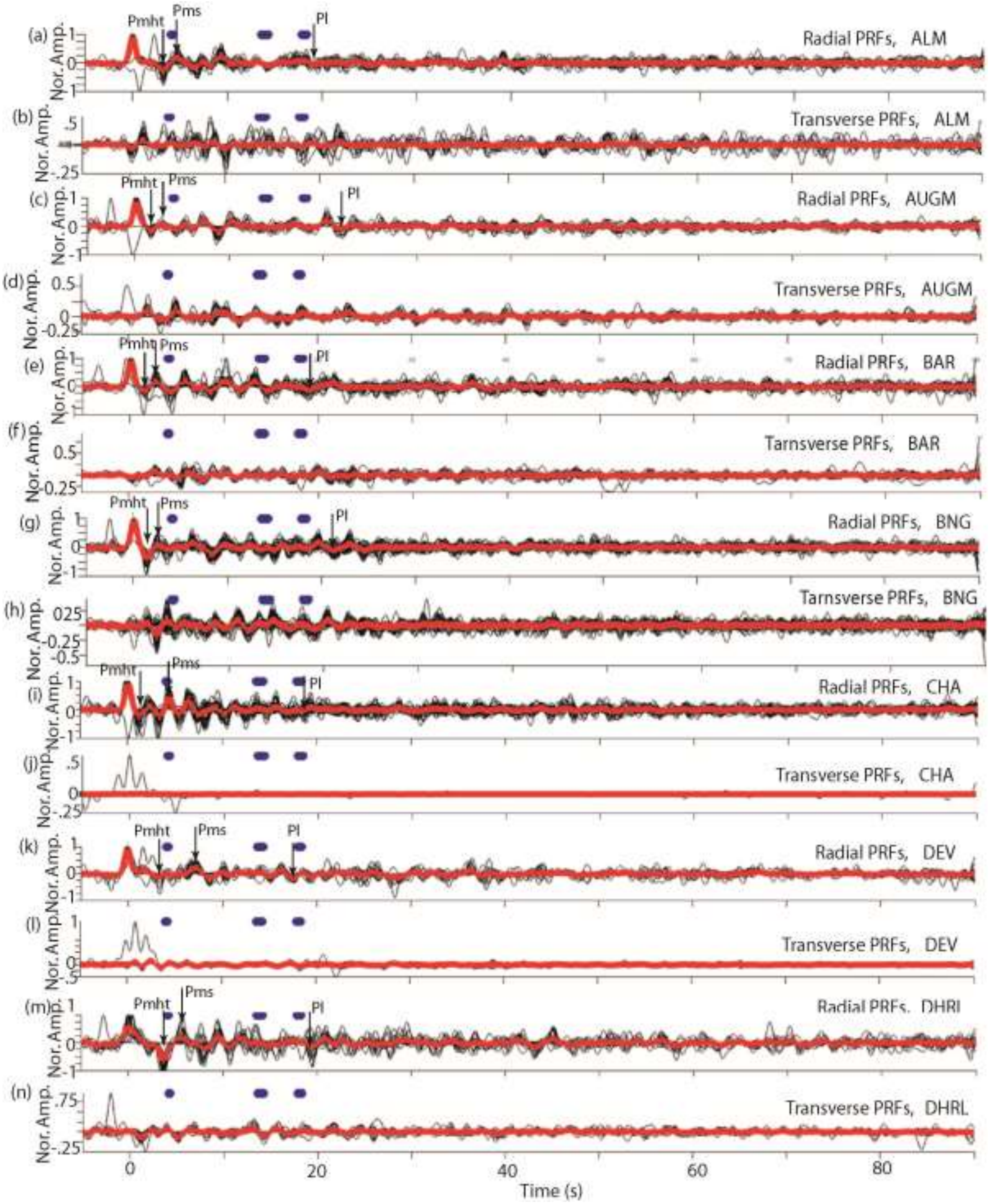


Figure S5: Stacked radial and transverse PRFs at seven broadband stations in the UK Himalaya, viz., (a-b) ALM, (c-d) AUGM, (e-f) BAR, (g-h) BNG, (i-j) CHA, (k-l) DEV, and (m-n) DHRL. Here, Pmht, Pms and Pl mark the negative conversion from MHT, positive conversion from the Moho and negative conversion from the Lithosphere-Asthenosphere boundary, respectively.

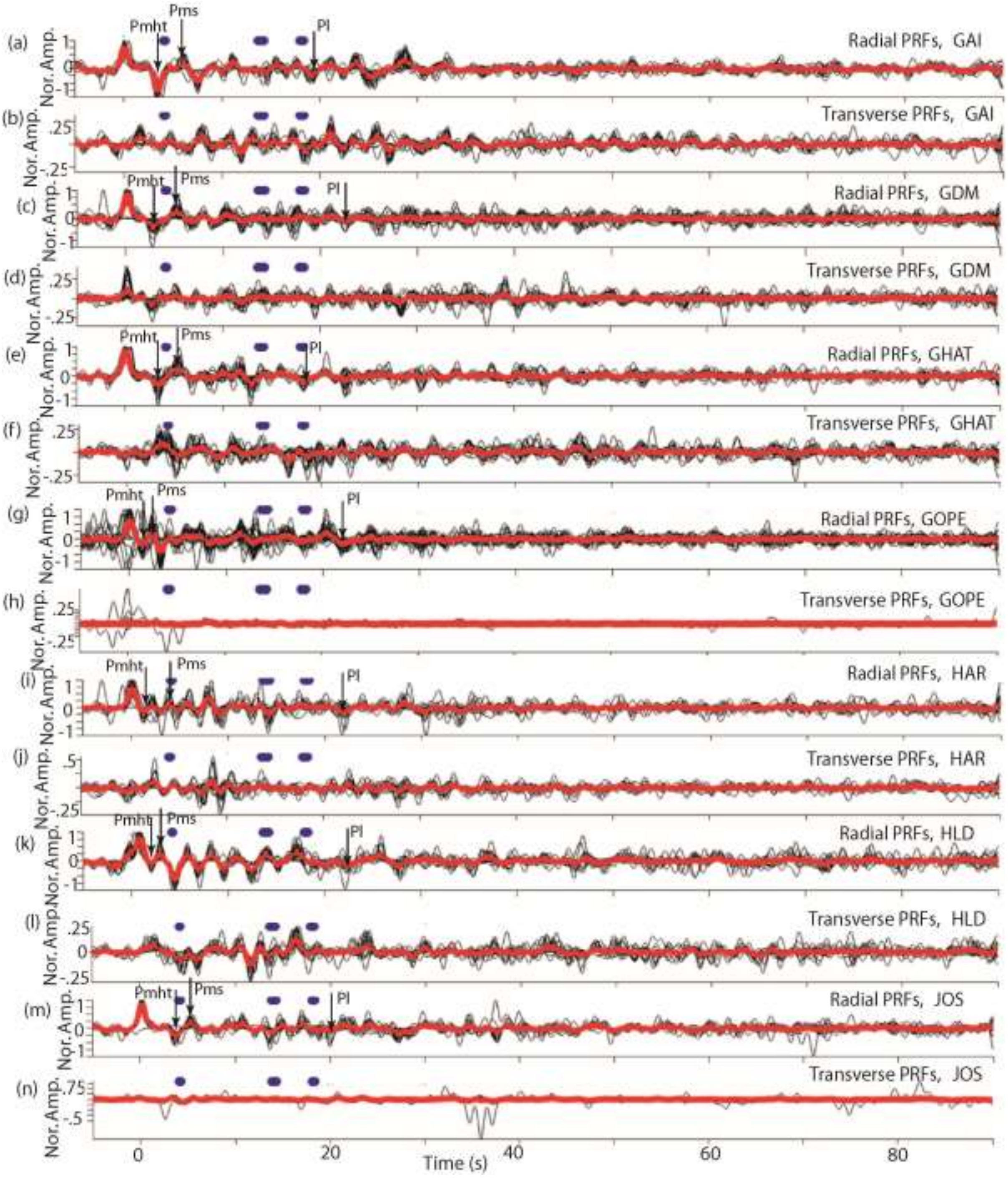


Figure S6: Stacked radial and transverse PRFs at seven broadband stations in the UK Himalaya, viz., (a-b) GAI, (c-d) GDM, (e-f) GHAT, (g-h) GOPE, (i-j) HAR, (k-l) HLD, and (m-n) JOS. Here, Pmht, Pms and Pl mark the negative conversion from MHT, positive conversion from the Moho and negative conversion from the Lithosphere-Asthenosphere boundary, respectively.

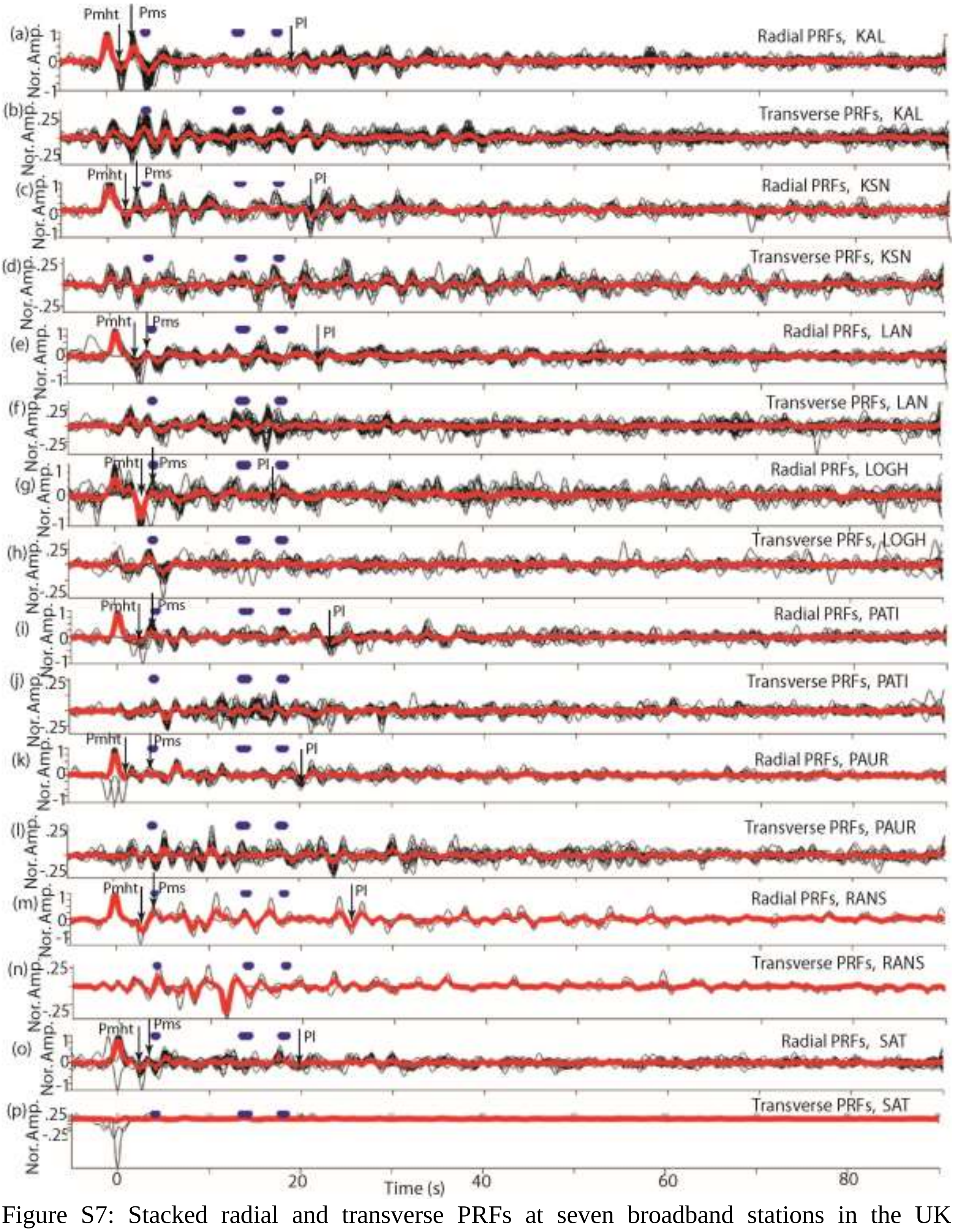


Figure S7: Stacked radial and transverse PRFs at seven broadband stations in the UK Himalaya, viz., (a-b) KAL, (c-d) KSN, (e-f) LAN, (g-h) LOGH, (i-j) PATI, (k-l) PAUR, (m-n) RANS and (o-p) SAT. Here, Pmht, Pms and Pl mark the negative conversion from MHT, positive conversion from the Moho and negative conversion from the Lithosphere-Asthenosphere boundary, respectively.

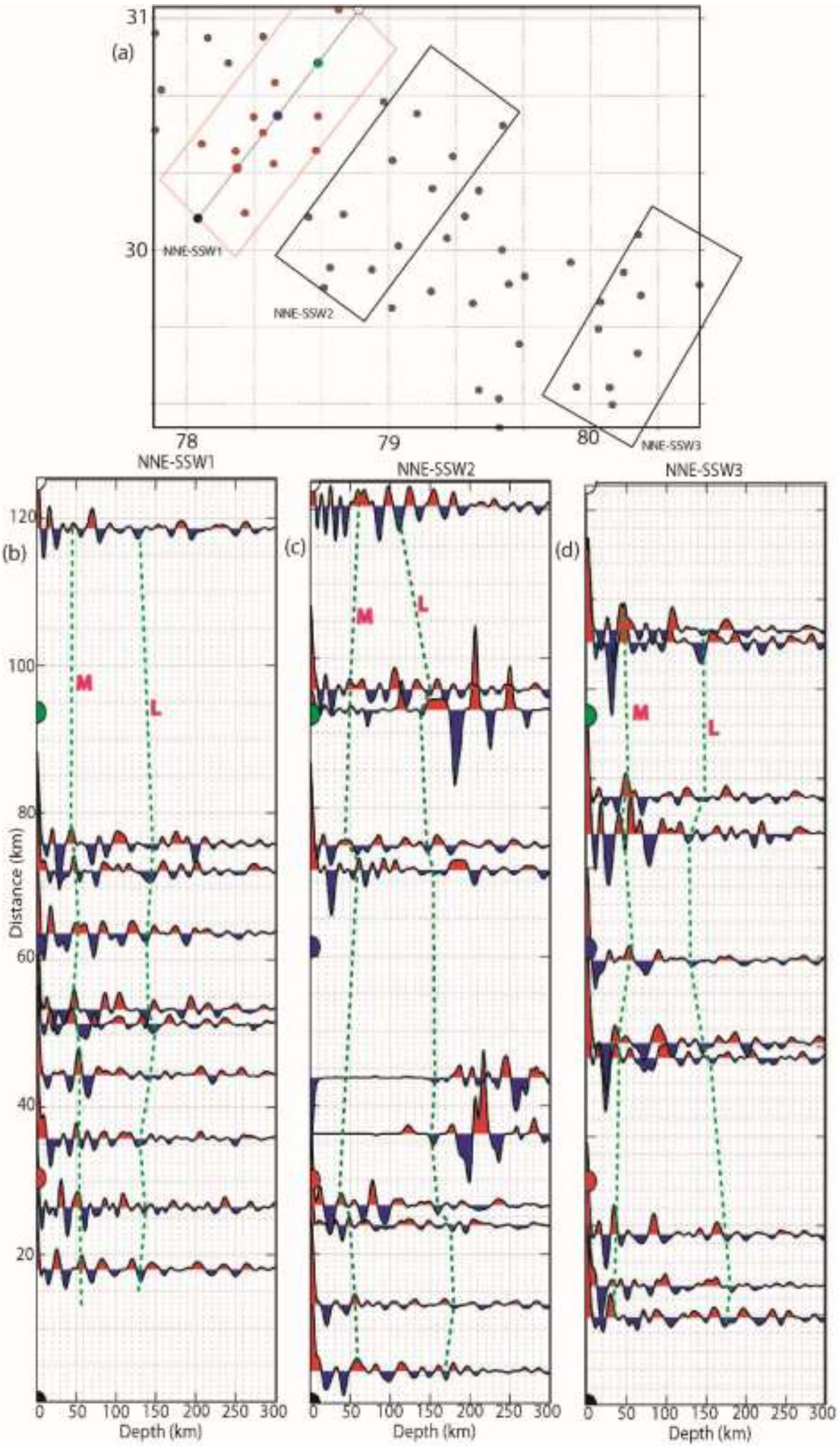


Figure S8: (a) Locations of three NNE-SSW profiles shown by three rectangular boxes of 55 km width. Migrated stacked PRFs with depth along (b) NNE-SSW1, (c) NNE-SSW2, and (d) NNE-SSW3 profiles. Green dotted lines mark variations in Moho depths (M) and lithosphere-asthenosphere boundaries (L).

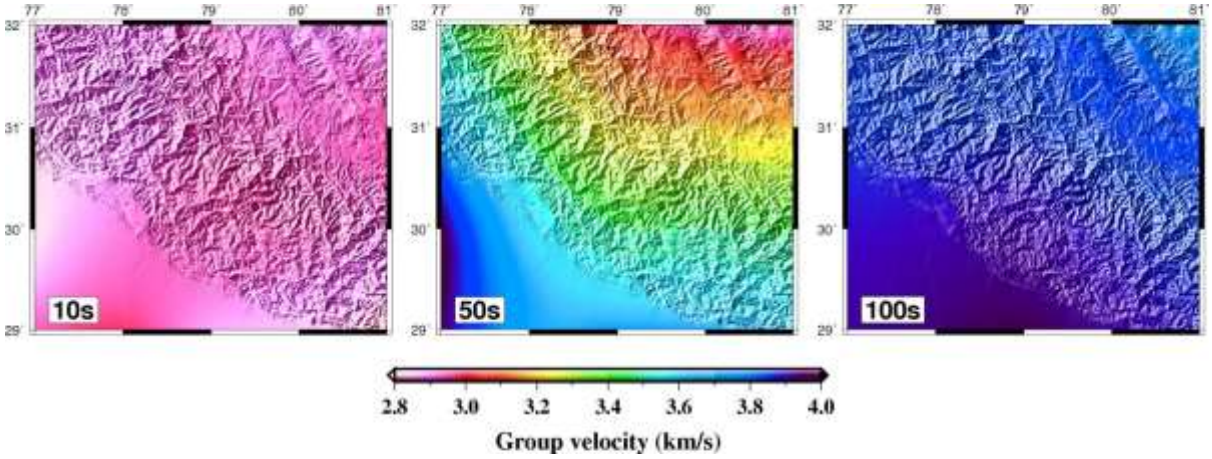


Figure S9: A representative Rayleigh wave group velocity map of the study region at 10s, 50s, and 100s time period, which are extracted from the tomograms of Saha et al.[3].

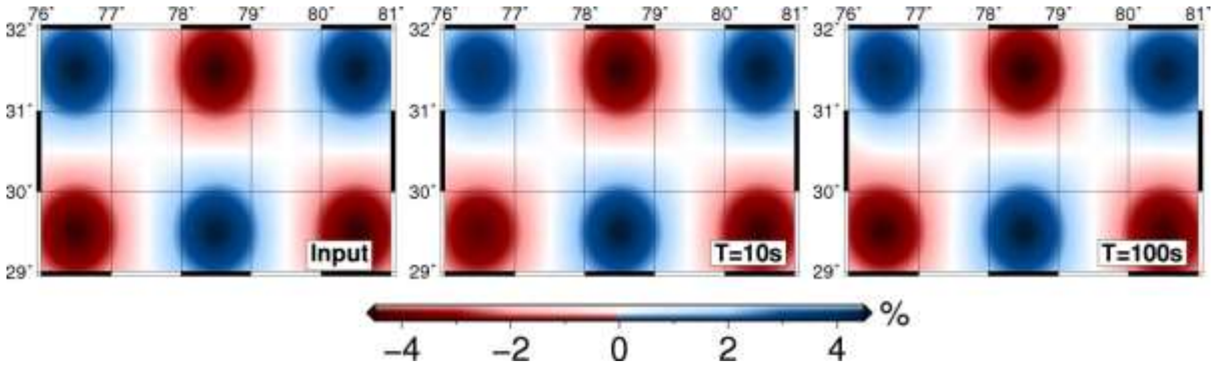


Figure S10: Results obtained from a checkerboard resolution test[3] of the Rayleigh wave tomography for the Uttarakhand region, for periods 10s and 100s.

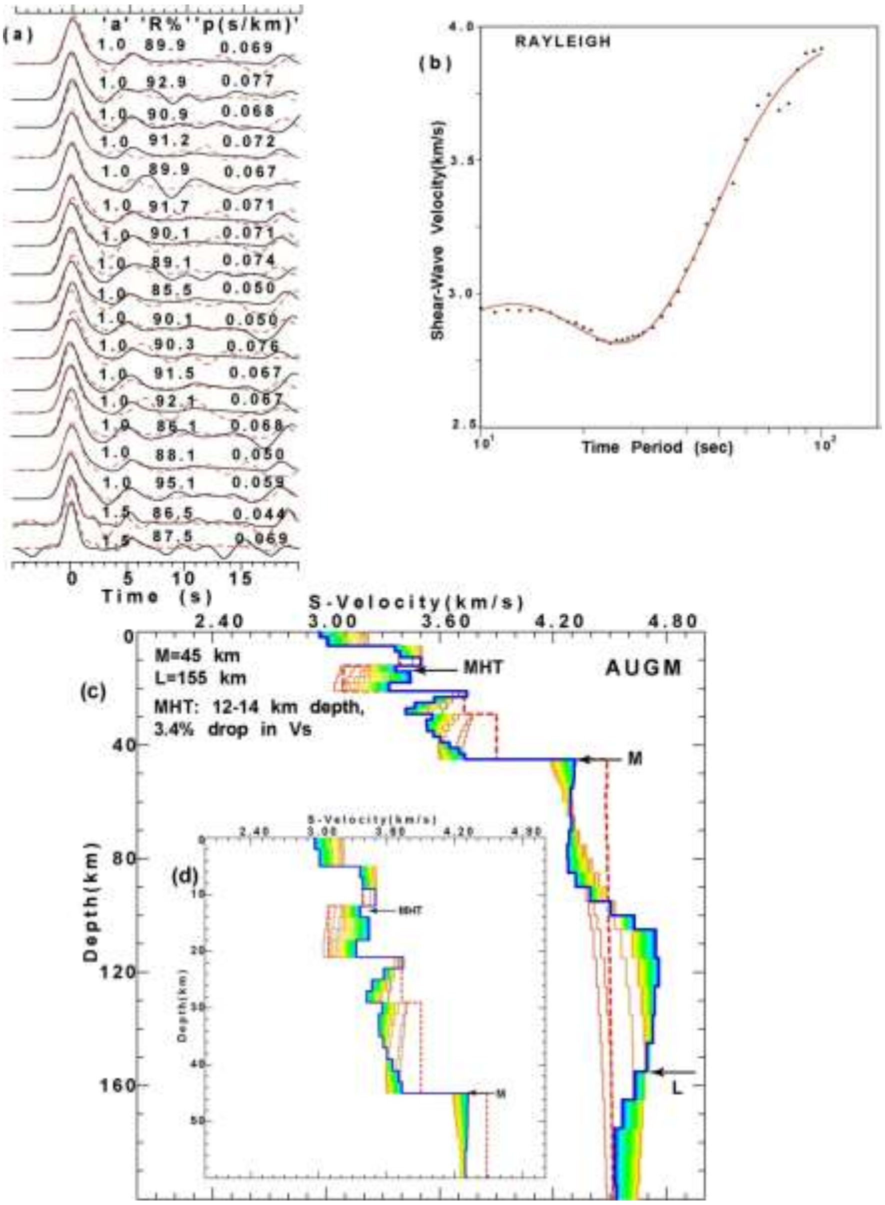


Fig S11: Results of joint inversion of P-RFs and fundamental mode surface wave group velocity dispersion (SWD) data at AUGM station, (a) showing good agreement between observed (black line) and inverted (red line) radial RFs with a=1.0, 1.5 and 2.0, for different horizontal slowness (S, in s/km). Here, "a" and "R%" represent Gaussian width factor (used for estimating RF) and agreement (in %) between observed and inverted RFs, respectively. Correlation between observed and inverted dispersion curves of (b) Rayleigh waves. (c) Inverted shear velocity models showing the main Himalayan thrust (MHT), Moho (M) and LAB (L) depth estimates in km. (d) A plot showing zoomed portion of the crustal part from the inverted Vs models. Different colours represent different Vs models used for the joint inversion. The initial shear velocity model is shown by a thick red dotted line, while the final shear velocity model is shown by a thick blue line, and (d) zoomed portion of figure (c) showing only crustal Vs model. Furthermore, MHT, M, and L mark the thickness of the Main Himalayan Thrust in km, Moho depths in km and lithospheric thickness in km, respectively.

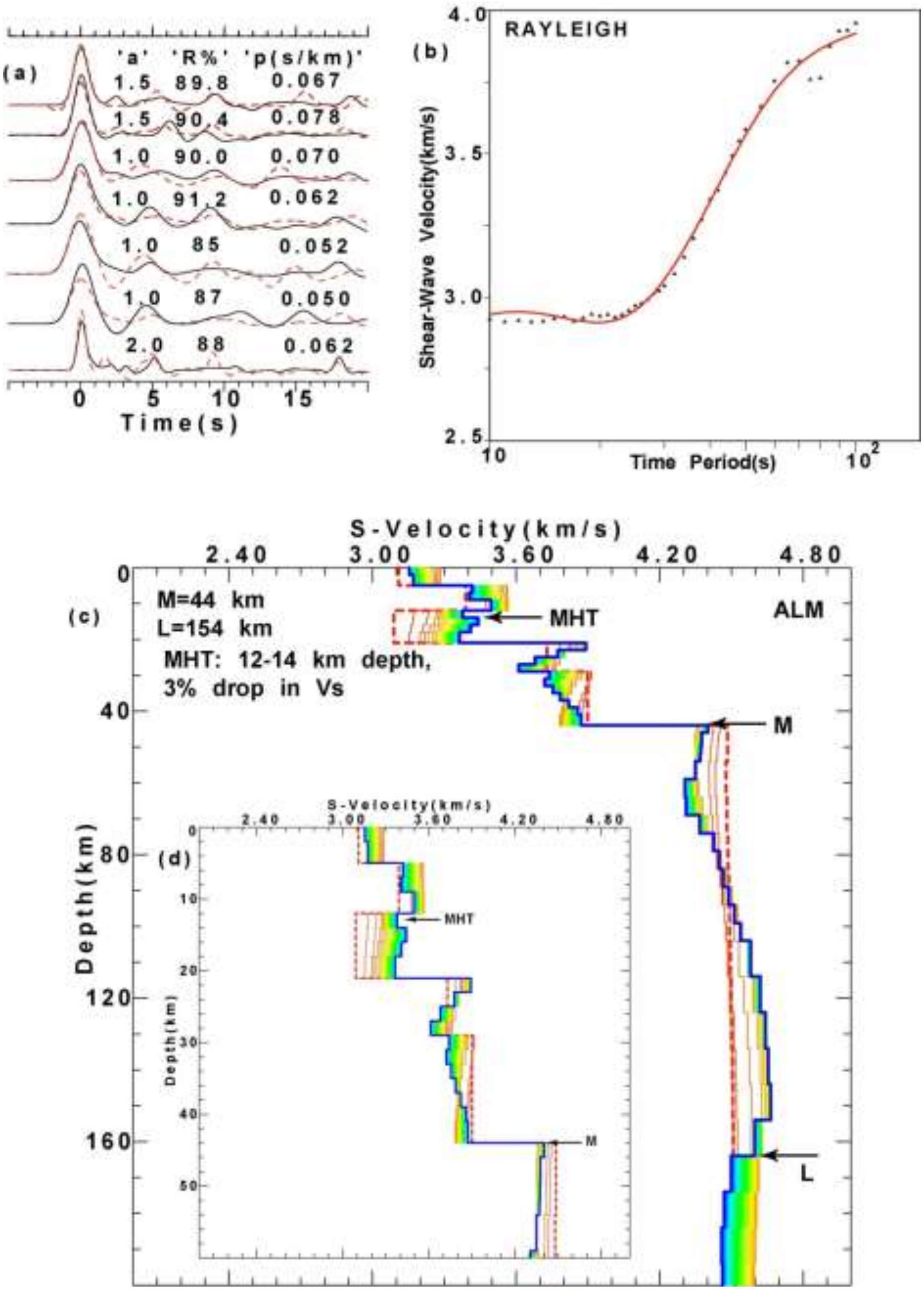


Fig S12: Results of joint inversion of P-RFs and fundamental mode surface wave group velocity dispersion (SWD) data at ALM station, (a) showing good agreement between observed (black line) and inverted (red line) radial RFs with a=1.0, 1.5 and 2.0, for different horizontal slowness (S, in s/km). Here, "a" and "R%" represent Gaussian width factor (used for estimating RF) and agreement (in %) between observed and inverted RFs, respectively. Correlation between observed and inverted dispersion curves of (b) Rayleigh waves. (c) Inverted shear velocity models showing the main Himalayan thrust (MHT), Moho (M) and LAB (L) depth estimates in km. (d) A plot showing zoomed portion of the crustal part from the inverted Vs models. Different colours represent different Vs models used for the joint inversion. The initial shear velocity model is shown by a thick red dotted line, while the final shear velocity model is shown by a thick blue line, and (d) zoomed portion of figure (c) showing only crustal Vs model. Furthermore, MHT, M, and L mark the thickness of the Main Himalayan Thrust in km, Moho depths in km and lithospheric thickness in km, respectively.

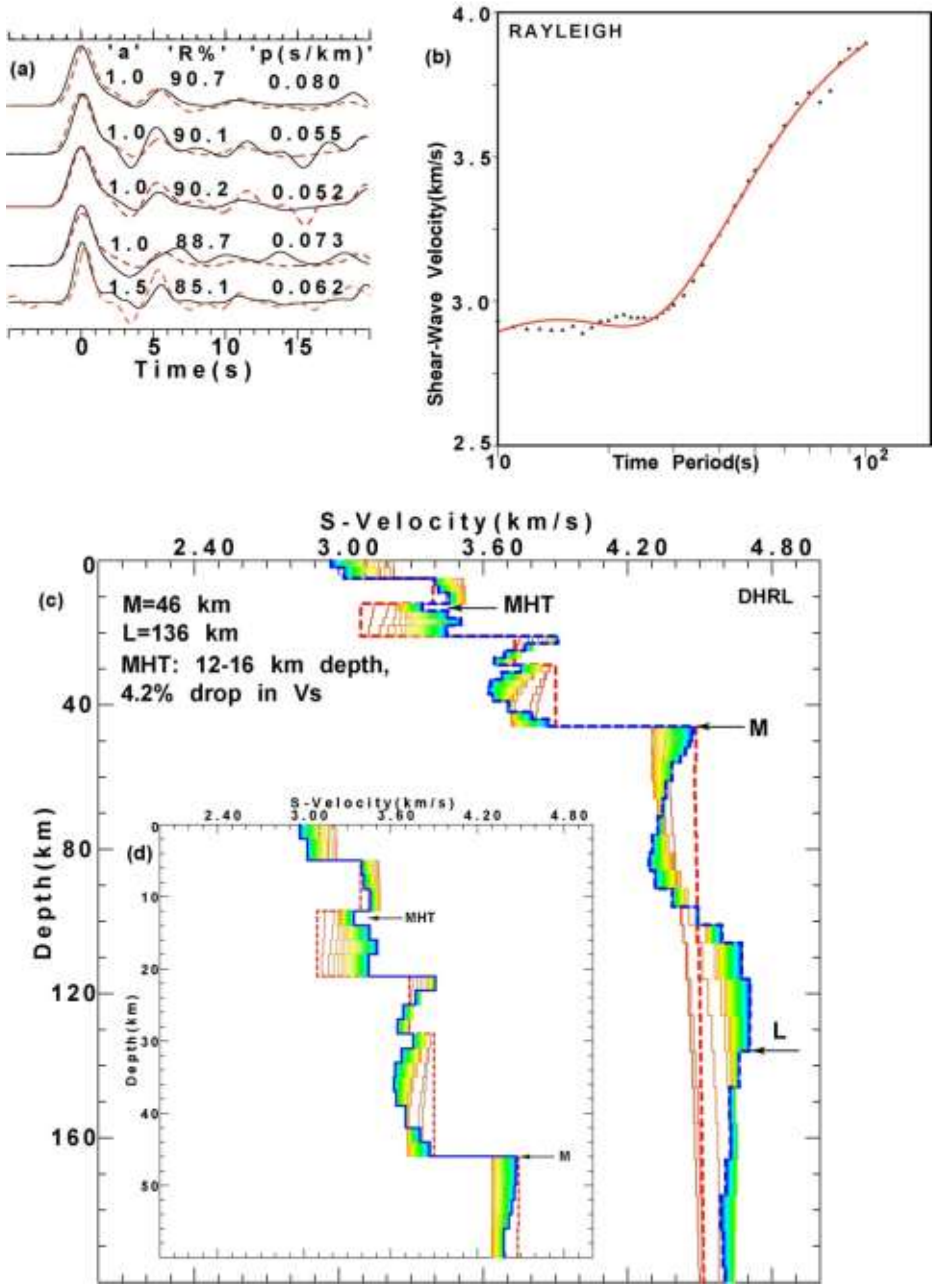


Fig S13: Results of joint inversion of P-RFs and fundamental mode surface wave group velocity dispersion (SWD) data at DHRL station, (a) showing good agreement between observed (black line) and inverted (red line) radial RFs with a=1.0, 1.5 and 2.0, for different horizontal slowness (S, in s/km). Here, "a" and "R%" represent Gaussian width factor (used for estimating RF) and agreement (in %) between observed and inverted RFs, respectively. Correlation between observed and inverted dispersion curves of (b) Rayleigh waves. (c) Inverted shear velocity models showing the main Himalayan thrust (MHT), Moho (M) and LAB (L) depth estimates in km. (d) A plot showing zoomed portion of the crustal part from the inverted Vs models. Different colours represent different Vs models used for the joint inversion. The initial shear velocity model is shown by a thick red dotted line, while the final shear velocity model is shown by a thick blue line, and (d) zoomed portion of figure (c) showing only crustal Vs model.

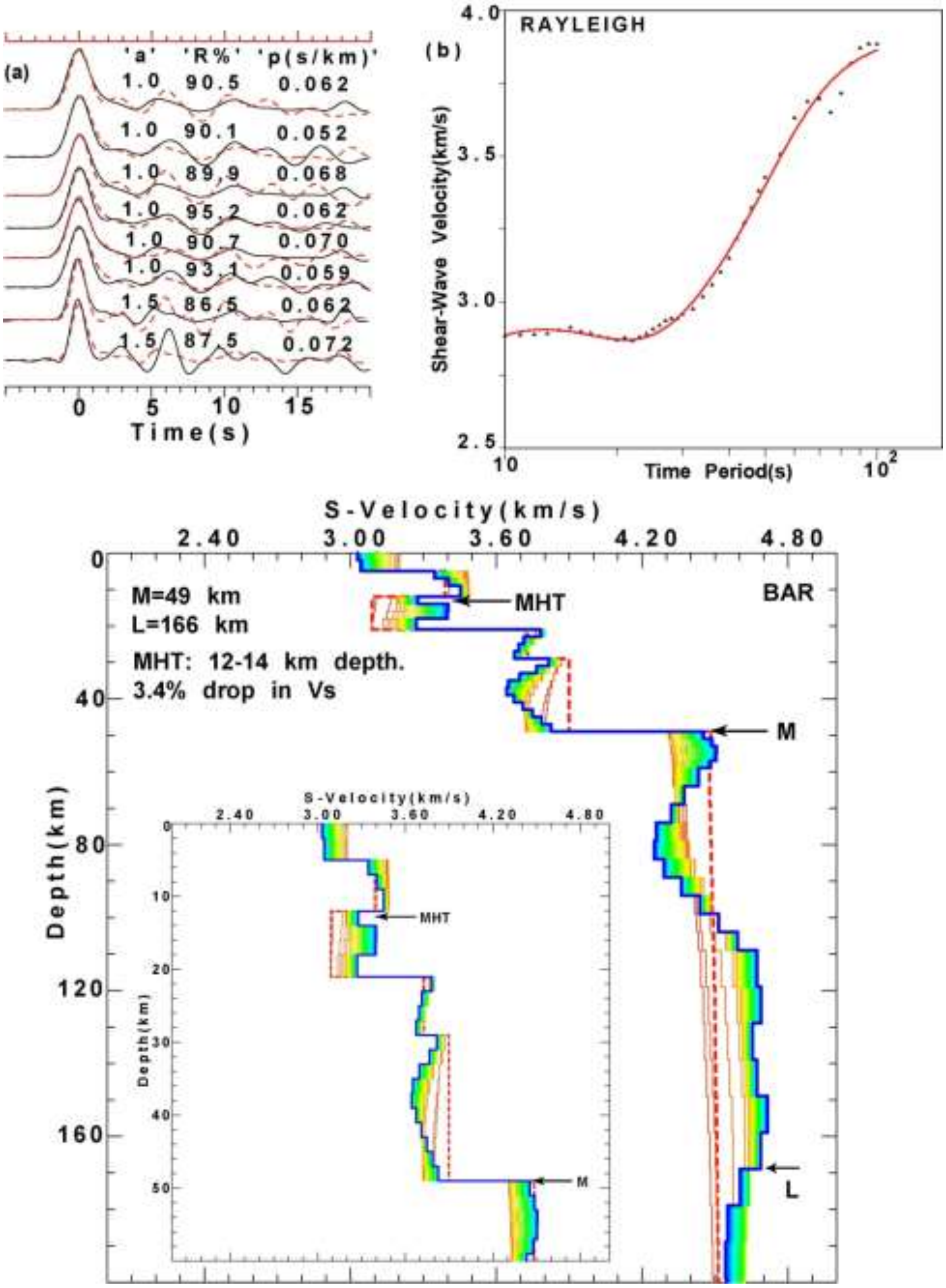


Fig S14: Results of joint inversion of P-RFs and fundamental mode surface wave group velocity dispersion (SWD) data at BAR station, (a) showing good agreement between observed (black line) and inverted (red line) radial RFs with a=1.0, 1.5 and 2.0, for different horizontal slowness (S, in s/km). Here, "a" and "R%" represent Gaussian width factor (used for estimating RF) and agreement (in %) between observed and inverted RFs, respectively. Correlation between observed and inverted dispersion curves of (b) Rayleigh waves. (c) Inverted shear velocity models showing the main Himalayan thrust (MHT), Moho (M) and LAB (L) depth estimates in km. Different colours represent different Vs models used for the joint inversion. The initial shear velocity model is shown by a thick red dotted line, while the final shear velocity model is shown by a thick blue line and (d) A plot showing zoomed portion of the crustal part from the inverted Vs models.

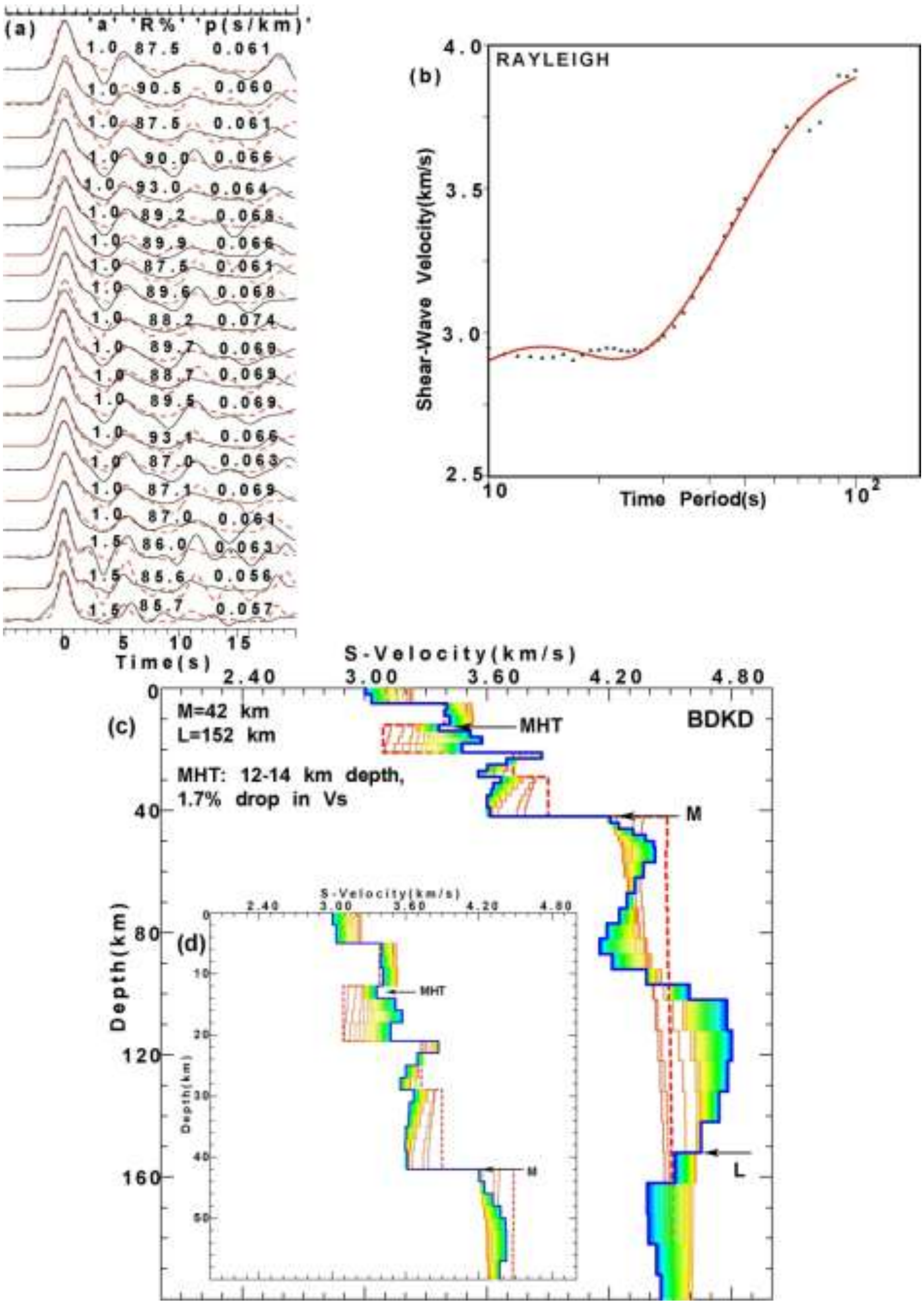


Fig S15: Results of joint inversion of P-RFs and fundamental mode surface wave group velocity dispersion (SWD) data at BDKD station, (a) showing good agreement between observed (black line) and inverted (red line) radial RFs with a=1.0, 1.5 and 2.0, for different horizontal slowness (S, in s/km). Here, "a" and "R%" represent Gaussian width factor (used for estimating RF) and agreement (in %) between observed and inverted RFs, respectively. Correlation between observed and inverted dispersion curves of (b) Rayleigh waves. (c) Inverted shear velocity models showing the main Himalayan thrust (MHT), Moho (M) and LAB (L) depth estimates in km. Different colours represent different Vs models used for the joint inversion. The initial shear velocity model is shown by a thick red dotted line, while the final shear velocity model is shown by a thick blue line and (d) A plot showing zoomed portion of the crustal part from the inverted Vs models.

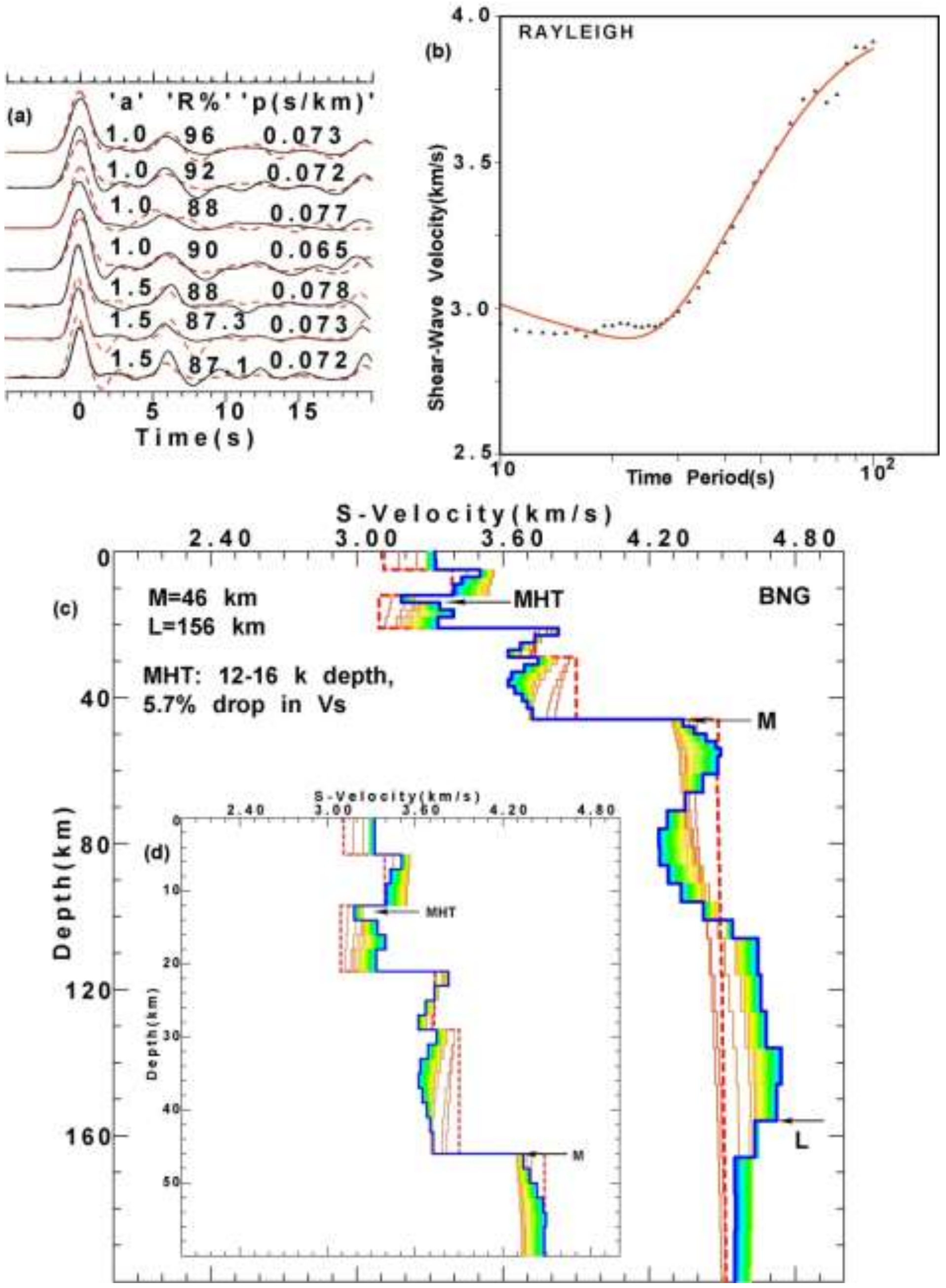


Fig S16: Results of joint inversion of P-RFs and fundamental mode surface wave group velocity dispersion (SWD) data at BNG station, (a) showing good agreement between observed (black line) and inverted (red line) radial RFs with a=1.0, 1.5 and 2.0, for different horizontal slowness (S, in s/km). Here, "a" and "R%" represent Gaussian width factor (used for estimating RF) and agreement (in %) between observed and inverted RFs, respectively. Correlation between observed and inverted dispersion curves of (b) Rayleigh waves. (c) Inverted shear velocity models showing the main Himalayan thrust (MHT), Moho (M) and LAB (L) depth estimates in km. Different colours represent different Vs models used for the joint inversion. The initial shear velocity model is shown by a thick red dotted line, while the final shear velocity model is shown by a thick blue line and (d) A plot showing zoomed portion of the crustal part from the inverted Vs models.

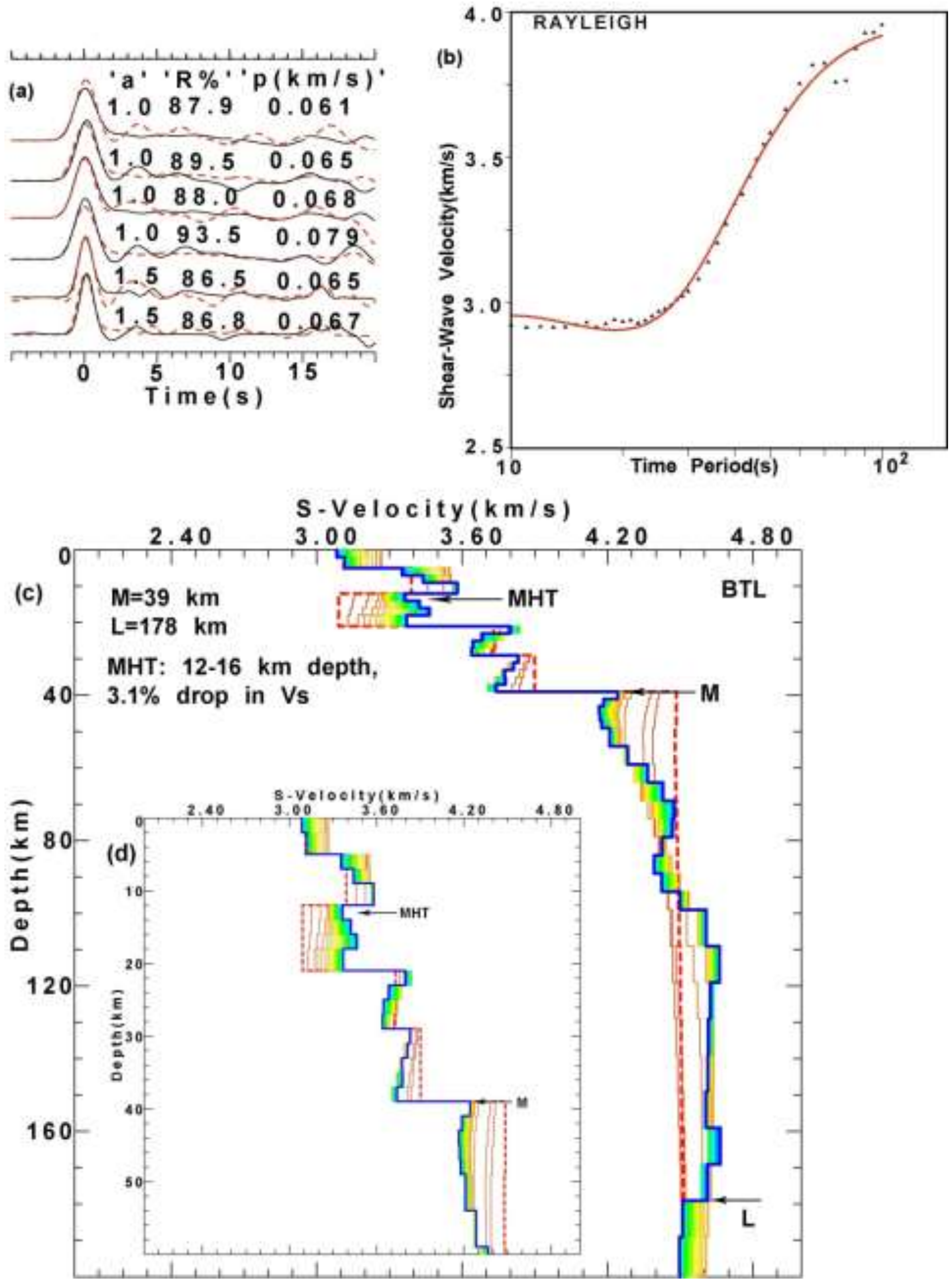


Fig S17: Results of joint inversion of P-RFs and fundamental mode surface wave group velocity dispersion (SWD) data at BTL station, (a) showing good agreement between observed (black line) and inverted (red line) radial RFs with a=1.0, 1.5 and 2.0, for different horizontal slowness (S, in s/km). Here, "a" and "R%" represent Gaussian width factor (used for estimating RF) and agreement (in %) between observed and inverted RFs, respectively. Correlation between observed and inverted dispersion curves of (b) Rayleigh waves. (c) Inverted shear velocity models showing the main Himalayan thrust (MHT), Moho (M) and LAB (L) depth estimates in km. Different colours represent different Vs models used for the joint inversion. The initial shear velocity model is shown by a thick red dotted line, while the final shear velocity model is shown by a thick blue line and (d) A plot showing zoomed portion of the crustal part from the inverted Vs models.

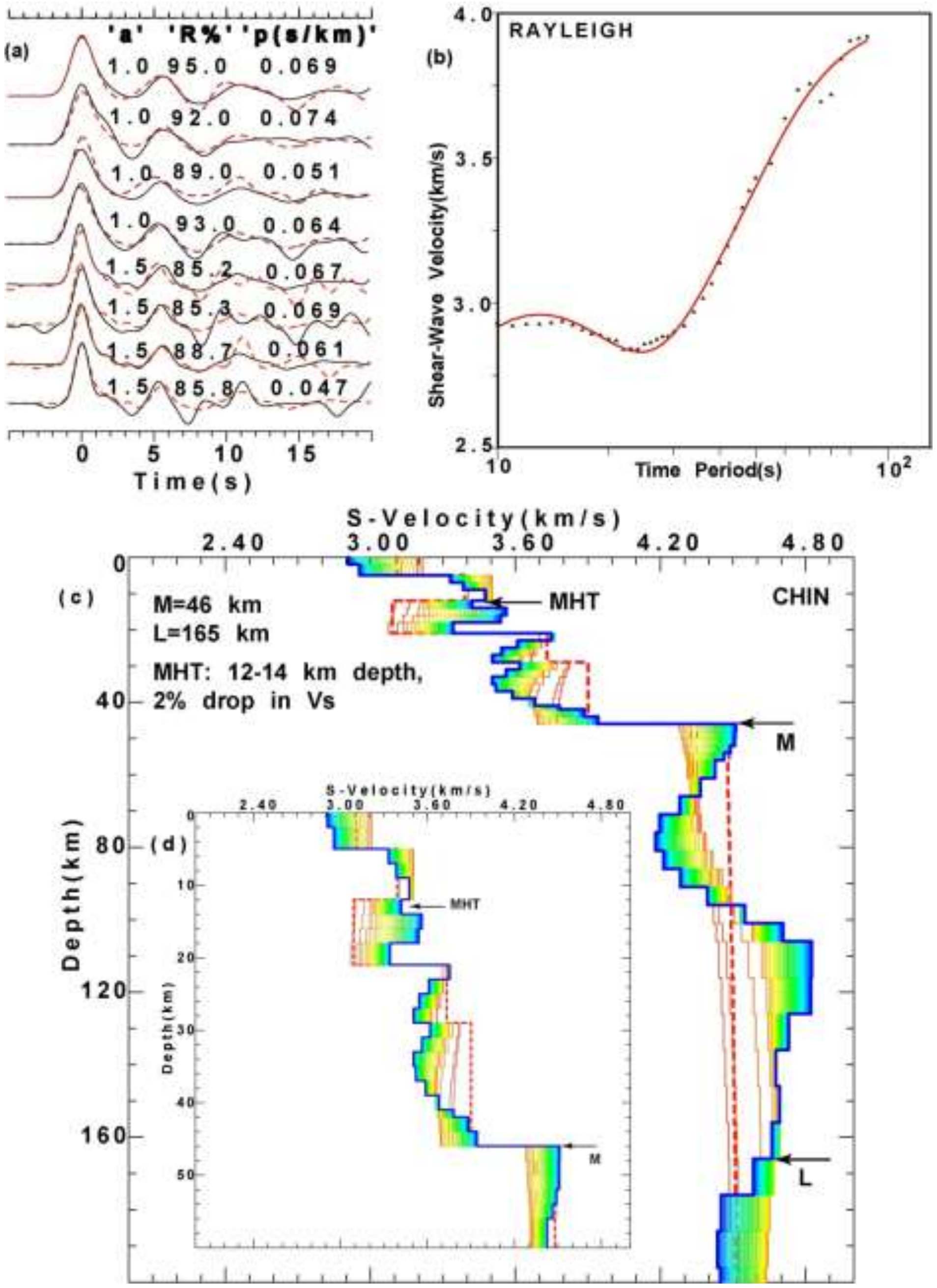


Fig S18: Results of joint inversion of P-RFs and fundamental mode surface wave group velocity dispersion (SWD) data at CHIN station, (a) showing good agreement between observed (black line) and inverted (red line) radial RFs with a=1.0, 1.5 and 2.0, for different horizontal slowness (S, in s/km). Here, "a" and "R%" represent Gaussian width factor (used for estimating RF) and agreement (in %) between observed and inverted RFs, respectively. Correlation between observed and inverted dispersion curves of (b) Rayleigh waves. (c) Inverted shear velocity models showing the main Himalayan thrust (MHT), Moho (M) and LAB (L) depth estimates in km. Different colours represent different Vs models used for the joint inversion. The initial shear velocity model is shown by a thick red dotted line, while the final shear velocity model is shown by a thick blue line and (d) A plot showing zoomed portion of the crustal part from the inverted Vs models.

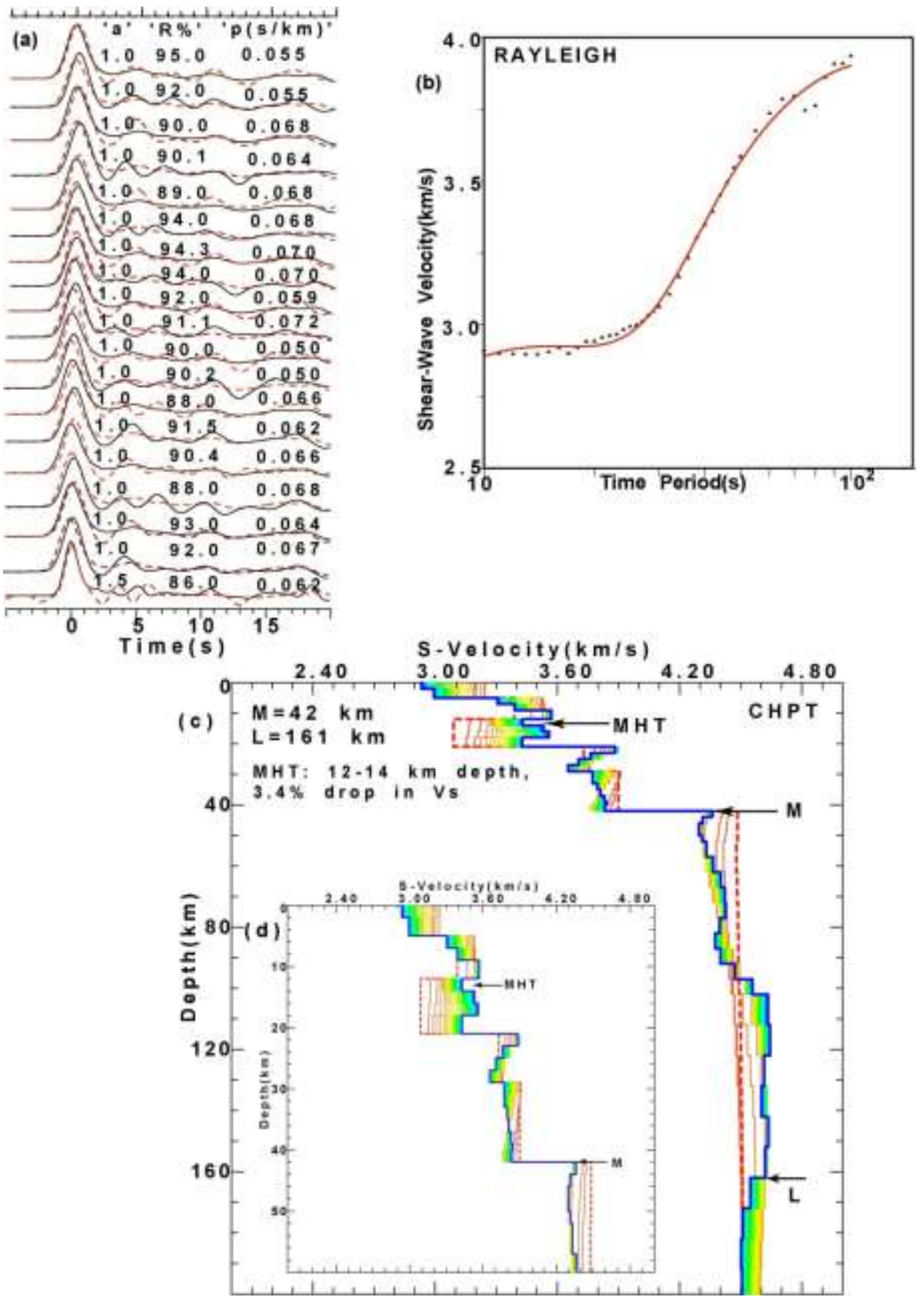


Fig S19: Results of joint inversion of P-RFs and fundamental mode surface wave group velocity dispersion (SWD) data at CHPT station, (a) showing good agreement between observed (black line) and inverted (red line) radial RFs with a=1.0, 1.5 and 2.0, for different horizontal slowness (S, in s/km). Here, "a" and "R%" represent Gaussian width factor (used for estimating RF) and agreement (in %) between observed and inverted RFs, respectively. Correlation between observed and inverted dispersion curves of (b) Rayleigh waves. (c) Inverted shear velocity models showing the main Himalayan thrust (MHT), Moho (M) and LAB (L) depth estimates in km. Different colours represent different Vs models used for the joint inversion. The initial shear velocity model is shown by a thick red dotted line, while the final shear velocity model is shown by a thick blue line and (d) A plot showing zoomed portion of the crustal part from the inverted Vs models.

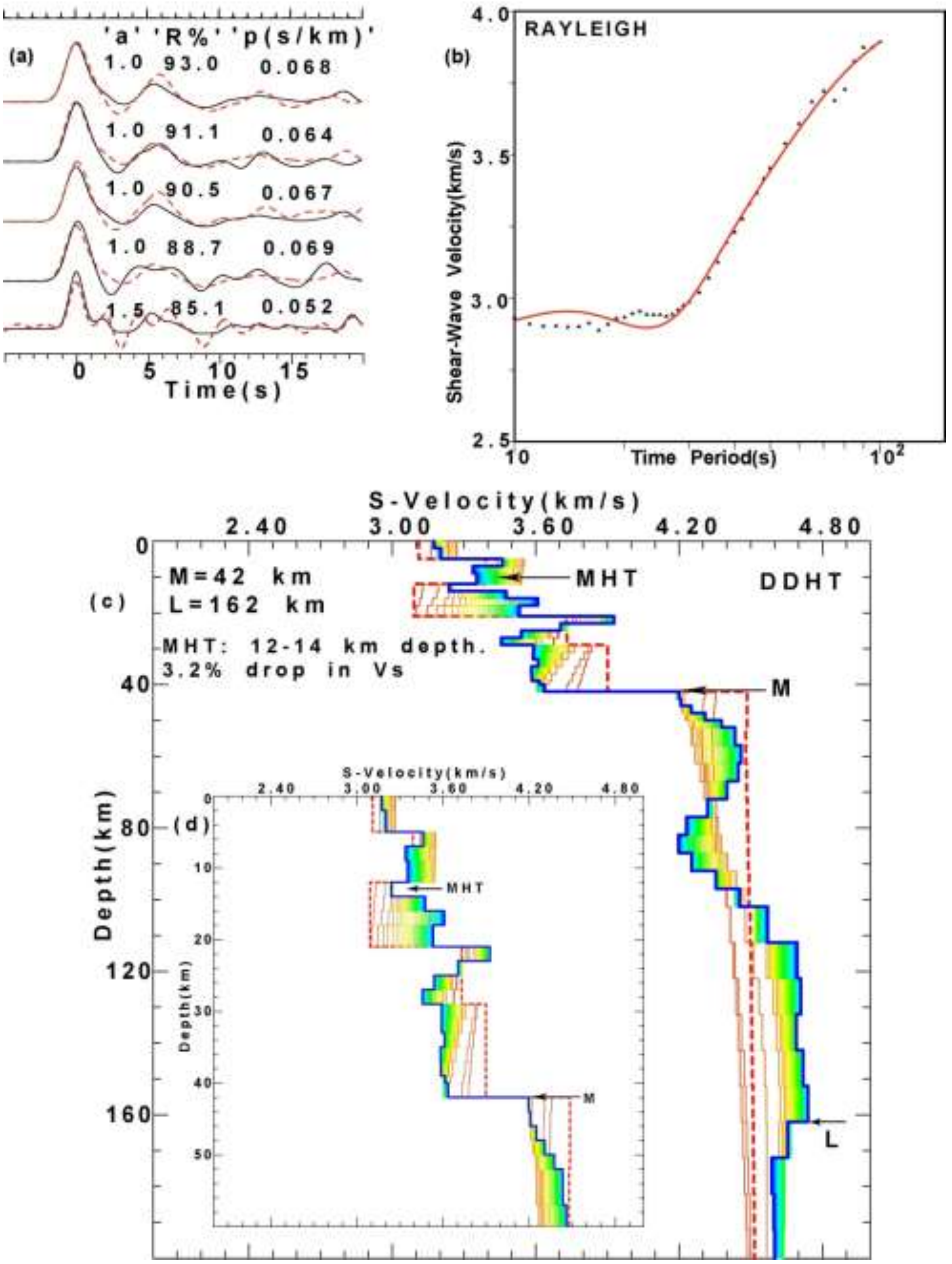


Fig S20: Results of joint inversion of P-RFs and fundamental mode surface wave group velocity dispersion (SWD) data at BDKD station, (a) showing good agreement between observed (black line) and inverted (red line) radial RFs with a=1.0, 1.5 and 2.0, for different horizontal slowness (S, in s/km). Here, "a" and "R%" represent Gaussian width factor (used for estimating RF) and agreement (in %) between observed and inverted RFs, respectively. Correlation between observed and inverted dispersion curves of (b) Rayleigh waves. (c) Inverted shear velocity models showing the main Himalayan thrust (MHT), Moho (M) and LAB (L) depth estimates in km. Different colours represent different Vs models used for the joint inversion. The initial shear velocity model is shown by a thick red dotted line, while the final shear velocity model is shown by a thick blue line and (d) A plot showing zoomed portion of the crustal part from the inverted Vs models.

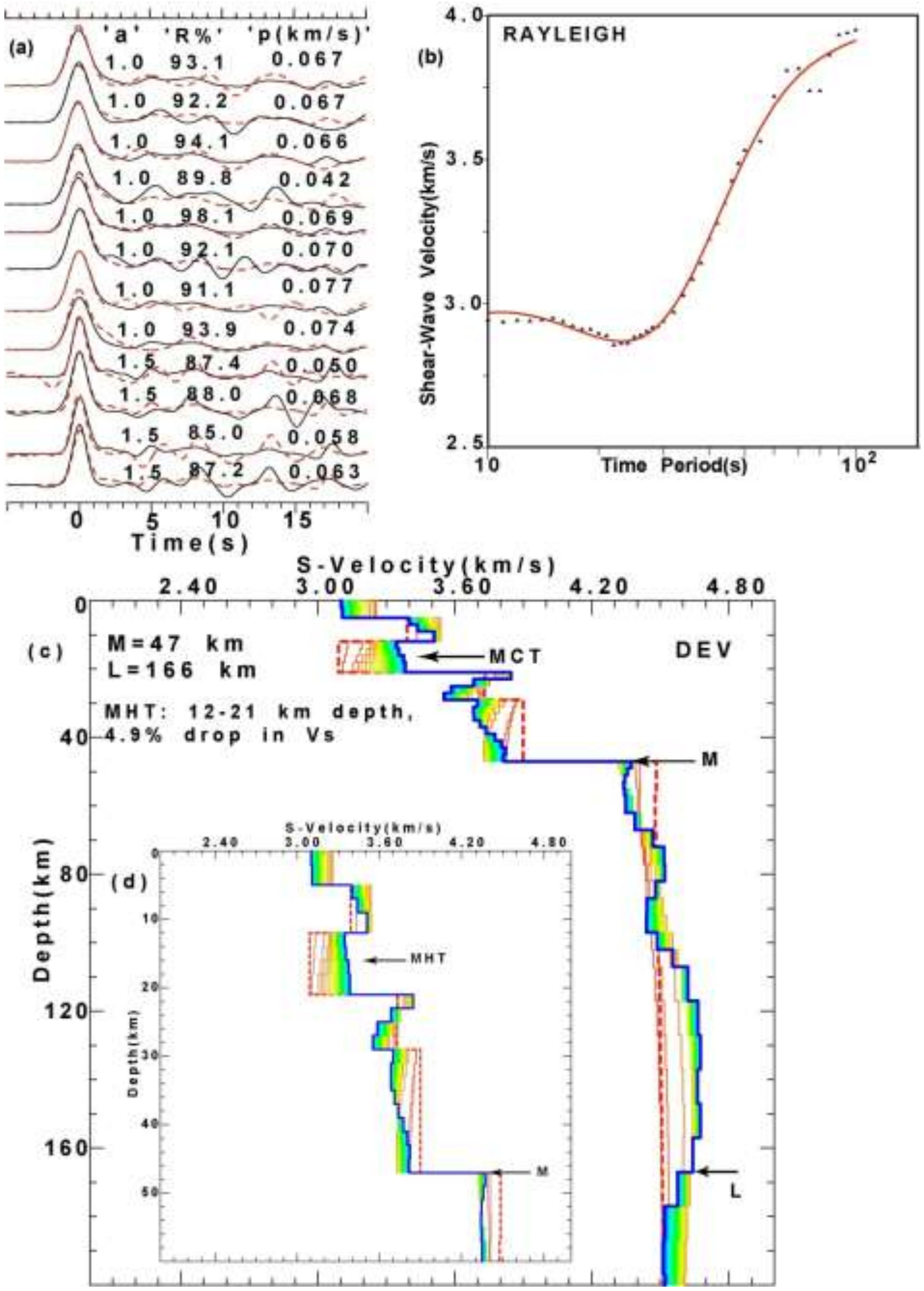


Fig S21: Results of joint inversion of P-RFs and fundamental mode surface wave group velocity dispersion (SWD) data at DEV station, (a) showing good agreement between observed (black line) and inverted (red line) radial RFs with a=1.0, 1.5 and 2.0, for different horizontal slowness (S, in s/km). Here, "a" and "R%" represent Gaussian width factor (used for estimating RF) and agreement (in %) between observed and inverted RFs, respectively. Correlation between observed and inverted dispersion curves of (b) Rayleigh waves. (c) Inverted shear velocity models showing the main Himalayan thrust (MHT), Moho (M) and LAB (L) depth estimates in km. Different colours represent different Vs models used for the joint inversion. The initial shear velocity model is shown by a thick red dotted line, while the final shear velocity model is shown by a thick blue line and (d) A plot showing zoomed portion of the crustal part from the inverted Vs models.

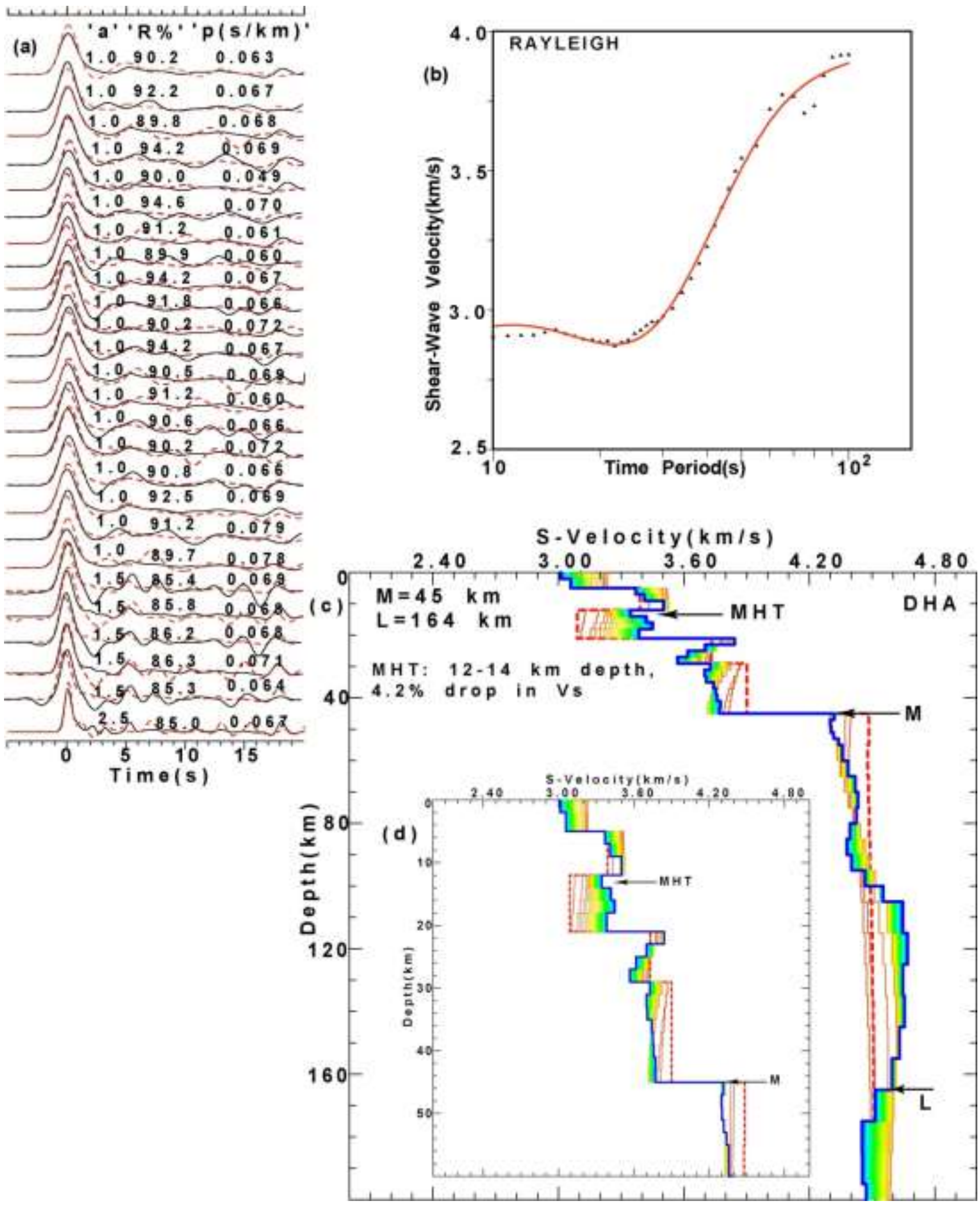


Fig S22: Results of joint inversion of P-RFs and fundamental mode surface wave group velocity dispersion (SWD) data at DHA station, (a) showing good agreement between observed (black line) and inverted (red line) radial RFs with a=1.0, 1.5 and 2.0, for different horizontal slowness (S, in s/km). Here, "a" and "R%" represent Gaussian width factor (used for estimating RF) and agreement (in %) between observed and inverted RFs, respectively. Correlation between observed and inverted dispersion curves of (b) Rayleigh waves. (c) Inverted shear velocity models showing the main Himalayan thrust (MHT), Moho (M) and LAB (L) depth estimates in km. Different colours represent different Vs models used for the joint inversion. The initial shear velocity model is shown by a thick red dotted line, while the final shear velocity model is shown by a thick blue line and (d) A plot showing zoomed portion of the crustal part from the inverted Vs models.

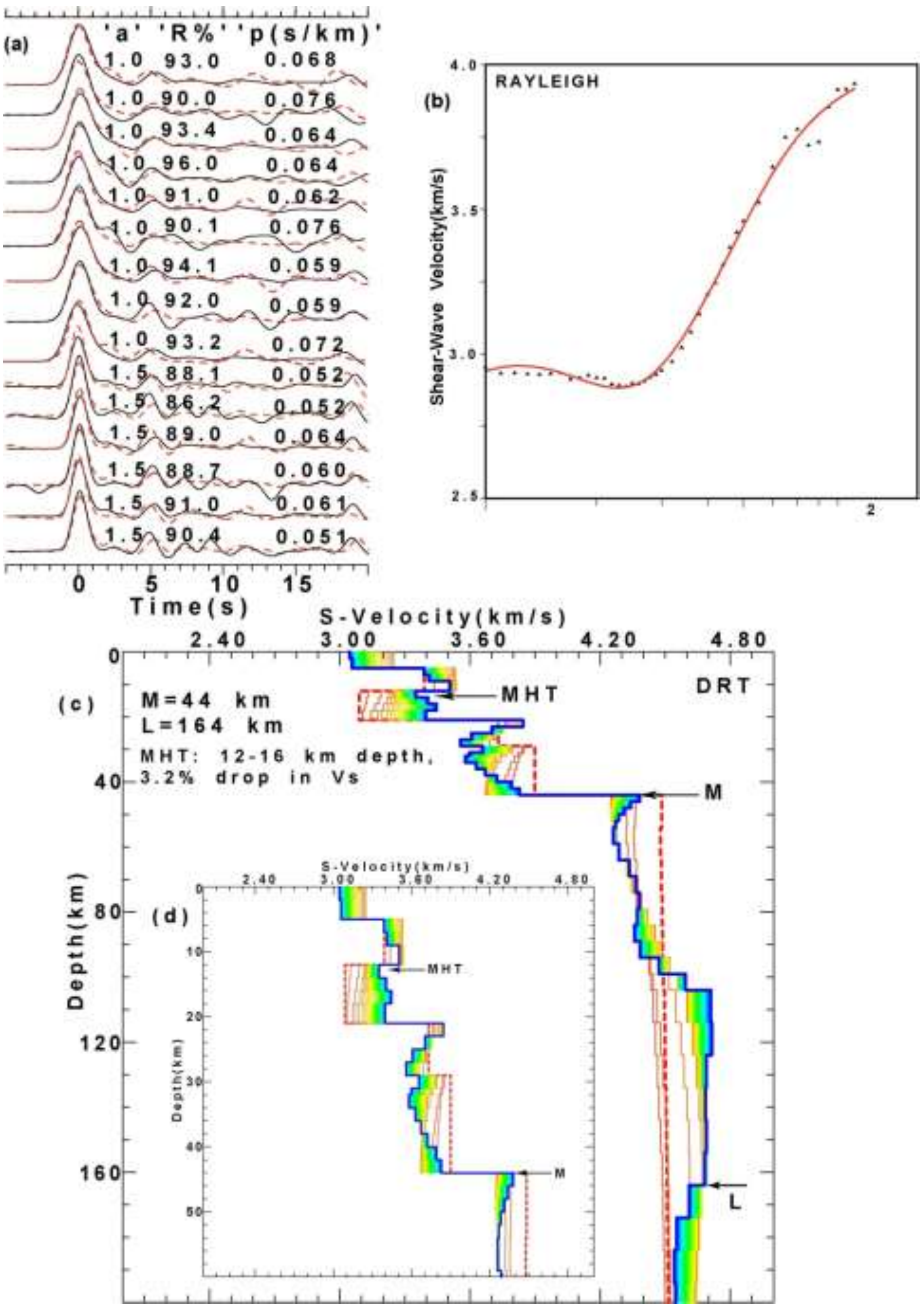


Fig S23: Results of joint inversion of P-RFs and fundamental mode surface wave group velocity dispersion (SWD) data at DRT station, (a) showing good agreement between observed (black line) and inverted (red line) radial RFs with a=1.0, 1.5 and 2.0, for different horizontal slowness (S, in s/km). Here, "a" and "R%" represent Gaussian width factor (used for estimating RF) and agreement (in %) between observed and inverted RFs, respectively. Correlation between observed and inverted dispersion curves of (b) Rayleigh waves. (c) Inverted shear velocity models showing the main Himalayan thrust (MHT), Moho (M) and LAB (L) depth estimates in km. Different colours represent different Vs models used for the joint inversion. The initial shear velocity model is shown by a thick red dotted line, while the final shear velocity model is shown by a thick blue line and (d) A plot showing zoomed portion of the crustal part from the inverted Vs models.

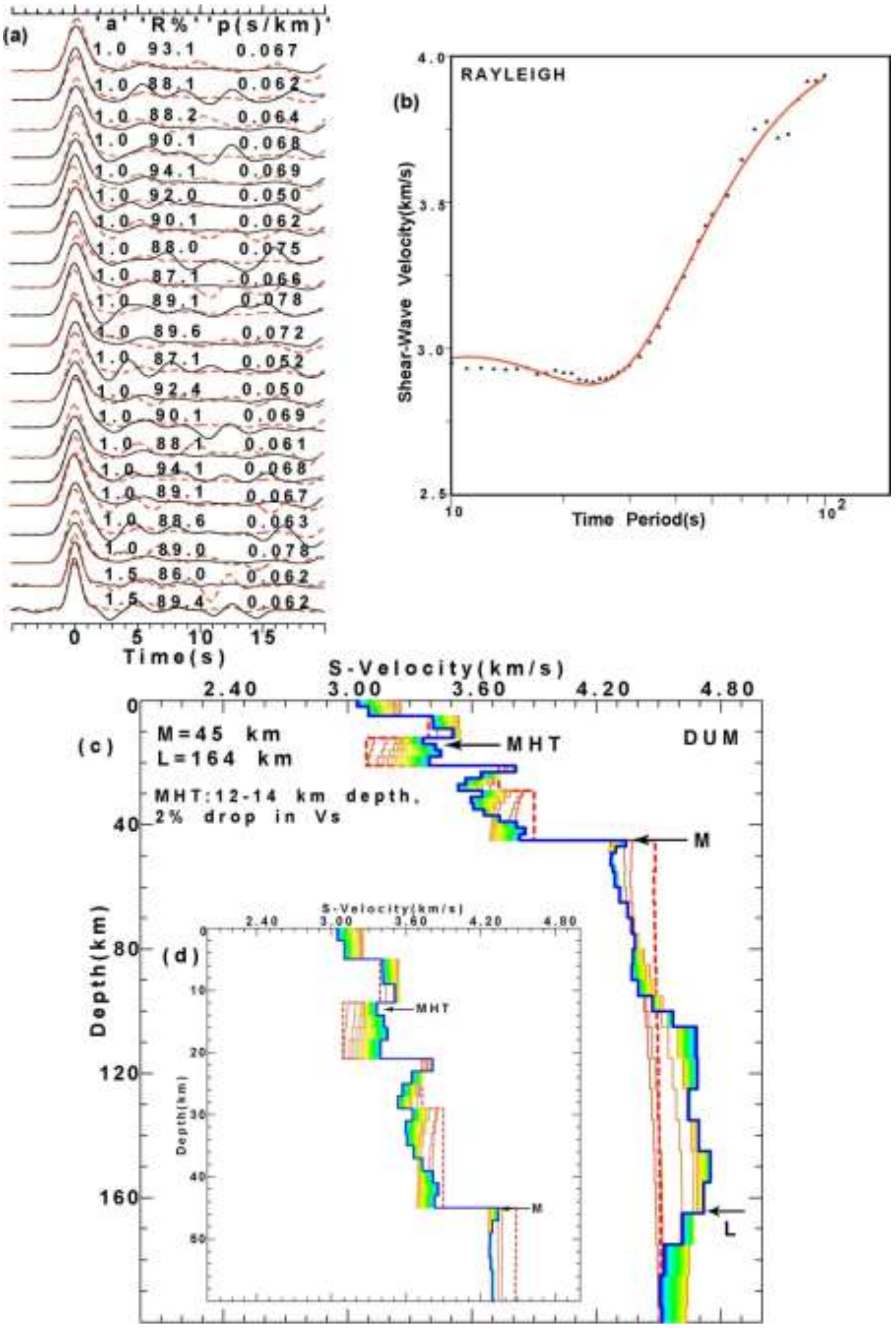

Fig S24: Results of joint inversion of P-RFs and fundamental mode surface wave group velocity dispersion (SWD) data at DUM station, (a) showing good agreement between observed (black line) and inverted (red line) radial RFs with a=1.0, 1.5 and 2.0, for different horizontal slowness (S, in s/km). Here, "a" and "R%" represent Gaussian width factor (used for estimating RF) and agreement (in %) between observed and inverted RFs, respectively. Correlation between observed and inverted dispersion curves of (b) Rayleigh waves. (c) Inverted shear velocity models showing the main Himalayan thrust (MHT), Moho (M) and LAB (L) depth estimates in km. Different colours represent different Vs models used for the joint inversion. The initial shear velocity model is shown by a thick red dotted line, while the final shear velocity model is shown by a thick blue line and (d) A plot showing zoomed portion of the crustal part from the inverted Vs models.

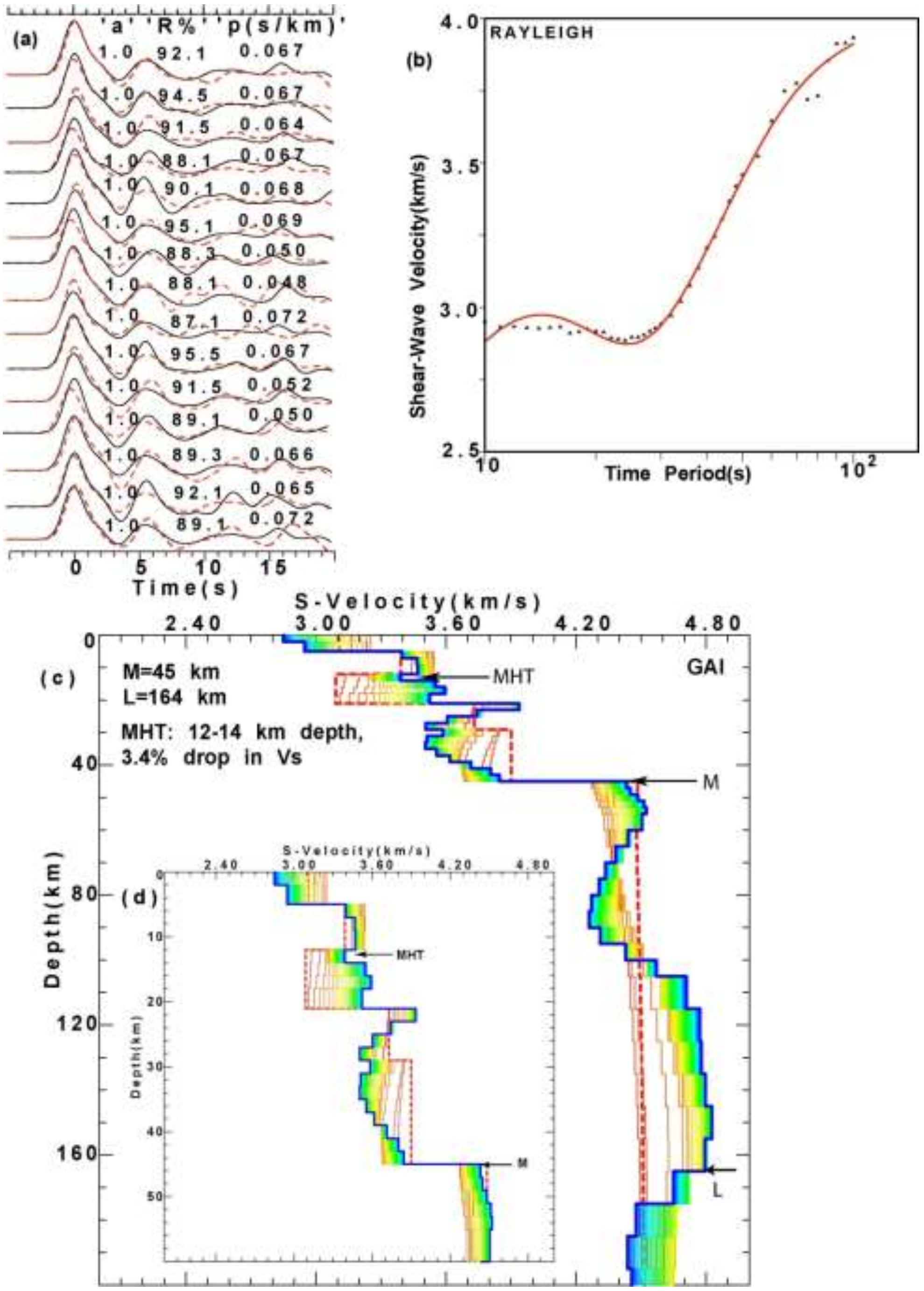

Fig S25: Results of joint inversion of P-RFs and fundamental mode surface wave group velocity dispersion (SWD) data at GAI station, (a) showing good agreement between observed (black line) and inverted (red line) radial RFs with a=1.0, 1.5 and 2.0, for different horizontal slowness (S, in s/km). Here, "a" and "R%" represent Gaussian width factor (used for estimating RF) and agreement (in %) between observed and inverted RFs, respectively. Correlation between observed and inverted dispersion curves of (b) Rayleigh waves. (c) Inverted shear velocity models showing the main Himalayan thrust (MHT), Moho (M) and LAB (L) depth estimates in km. Different colours represent different Vs models used for the joint inversion. The initial shear velocity model is shown by a thick red dotted line, while the final shear velocity model is shown by a thick blue line and (d) A plot showing zoomed portion of the crustal part from the inverted Vs models.

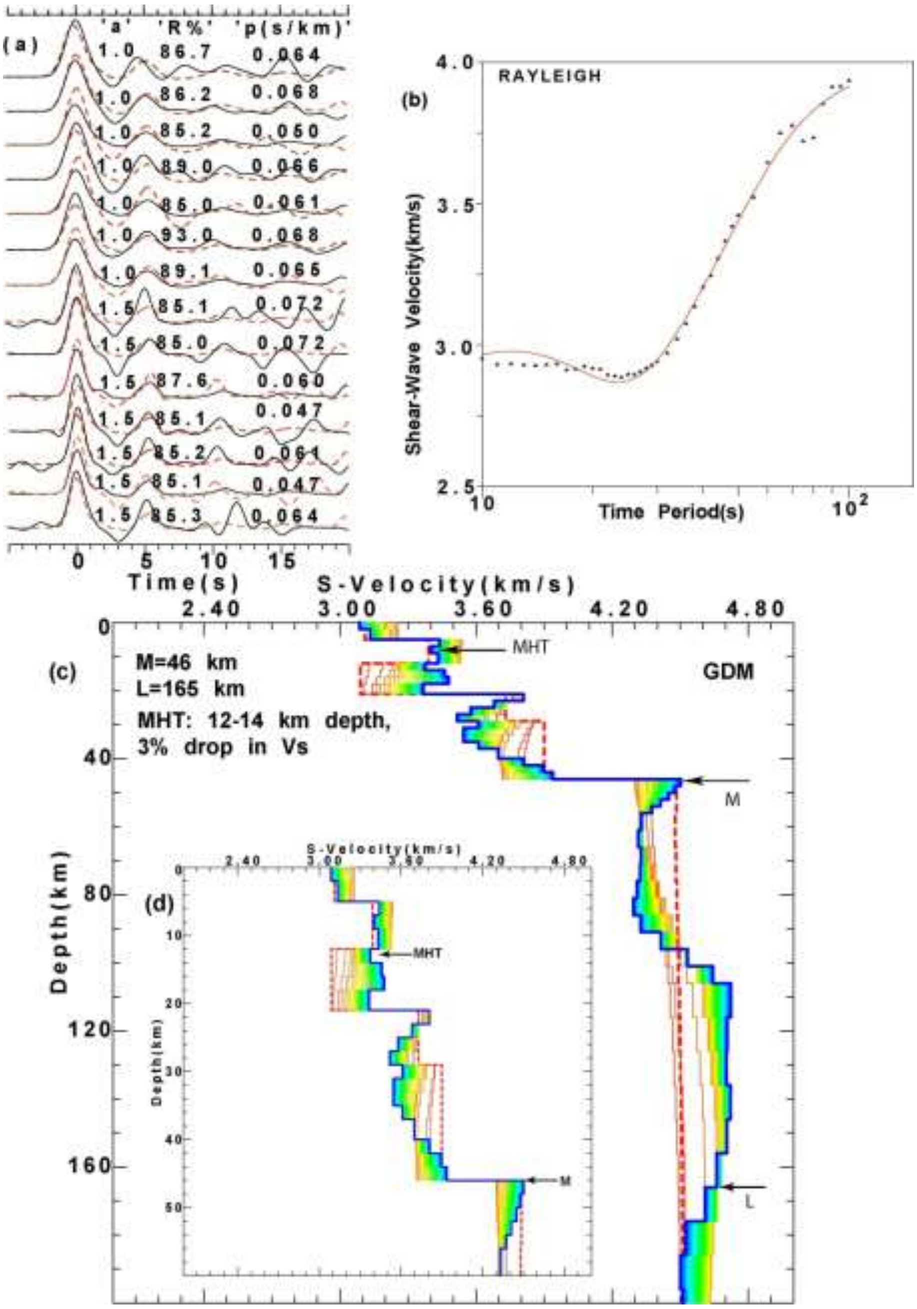


Fig S26: Results of joint inversion of P-RFs and fundamental mode surface wave group velocity dispersion (SWD) data at GDM station, (a) showing good agreement between observed (black line) and inverted (red line) radial RFs with a=1.0, 1.5 and 2.0, for different horizontal slowness (S, in s/km). Here, "a" and "R%" represent Gaussian width factor (used for estimating RF) and agreement (in %) between observed and inverted RFs, respectively. Correlation between observed and inverted dispersion curves of (b) Rayleigh waves. (c) Inverted shear velocity models showing the main Himalayan thrust (MHT), Moho (M) and LAB (L) depth estimates in km. Different colours represent different Vs models used for the joint inversion. The initial shear velocity model is shown by a thick red dotted line, while the final shear velocity model is shown by a thick blue line and (d) A plot showing zoomed portion of the crustal part from the inverted Vs models.

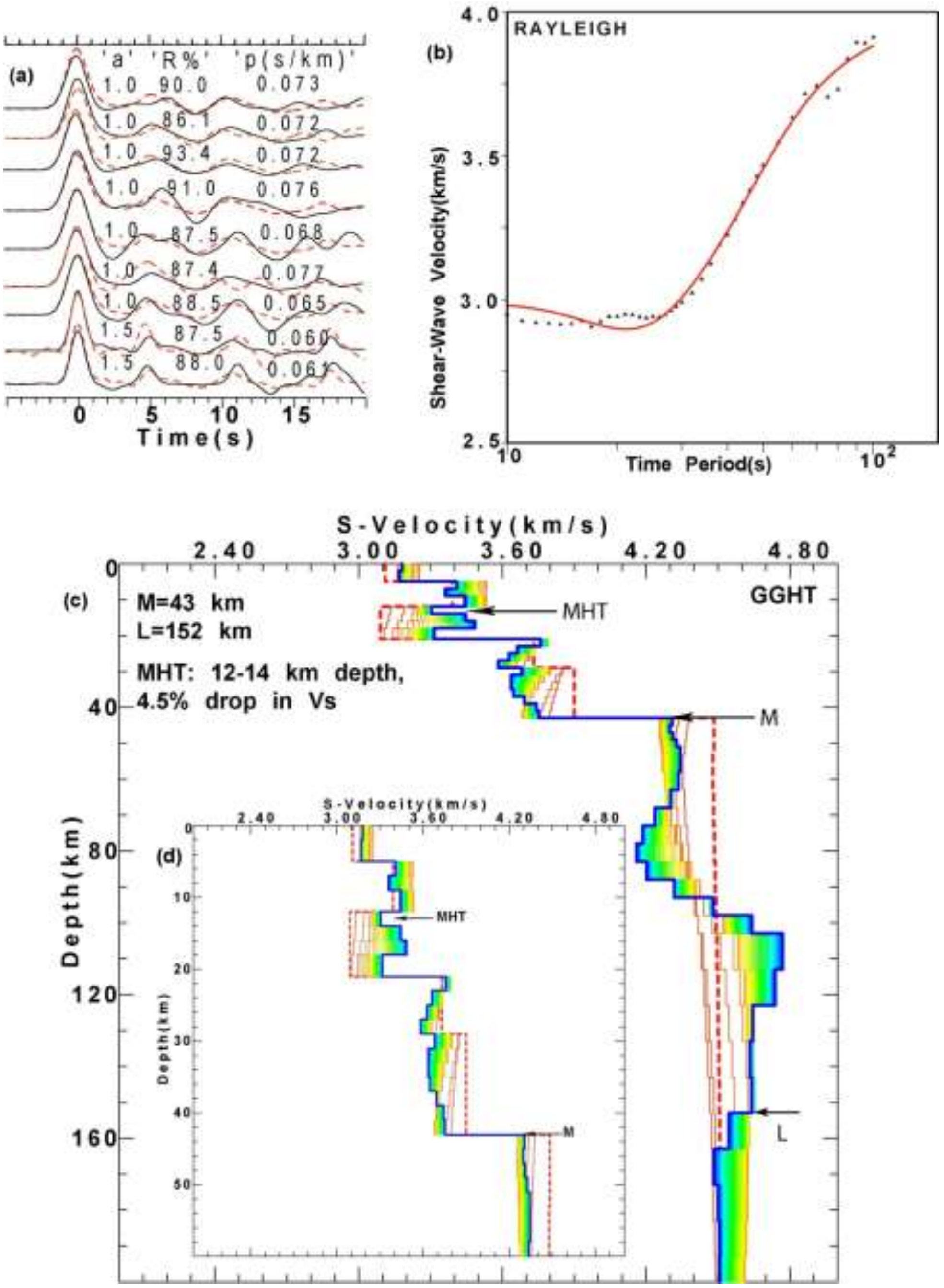


Fig S27: Results of joint inversion of P-RFs and fundamental mode surface wave group velocity dispersion (SWD) data at GGHT station, (a) showing good agreement between observed (black line) and inverted (red line) radial RFs with a=1.0, 1.5 and 2.0, for different horizontal slowness (S, in s/km). Here, "a" and "R%" represent Gaussian width factor (used for estimating RF) and agreement (in %) between observed and inverted RFs, respectively. Correlation between observed and inverted dispersion curves of (b) Rayleigh waves. (c) Inverted shear velocity models showing the main Himalayan thrust (MHT), Moho (M) and LAB (L) depth estimates in km. Different colours represent different Vs models used for the joint inversion. The initial shear velocity model is shown by a thick red dotted line, while the final shear velocity model is shown by a thick blue line and (d) A plot showing zoomed portion of the crustal part from the inverted Vs models.

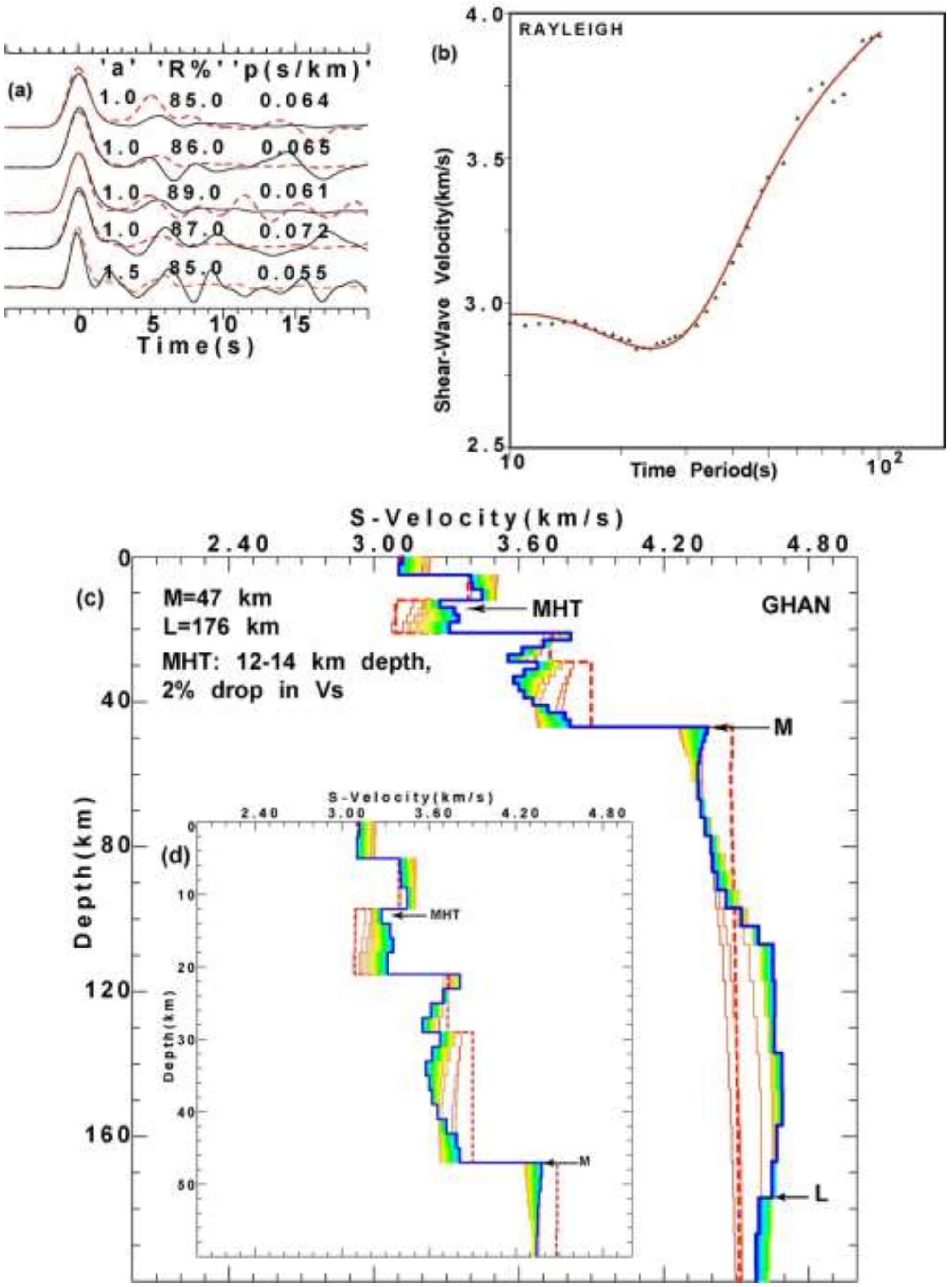


Fig 28: Results of joint inversion of P-RFs and fundamental mode surface wave group velocity dispersion (SWD) data at GHAN station, (a) showing good agreement between observed (black line) and inverted (red line) radial RFs with a=1.0, 1.5 and 2.0, for different horizontal slowness (S, in s/km). Here, "a" and "R%" represent Gaussian width factor (used for estimating RF) and agreement (in %) between observed and inverted RFs, respectively. Correlation between observed and inverted dispersion curves of (b) Rayleigh waves. (c) Inverted shear velocity models showing the main Himalayan thrust (MHT), Moho (M) and LAB (L) depth estimates in km. Different colours represent different Vs models used for the joint inversion. The initial shear velocity model is shown by a thick red dotted line, while the final shear velocity model is shown by a thick blue line and (d) A plot showing zoomed portion of the crustal part from the inverted Vs models.

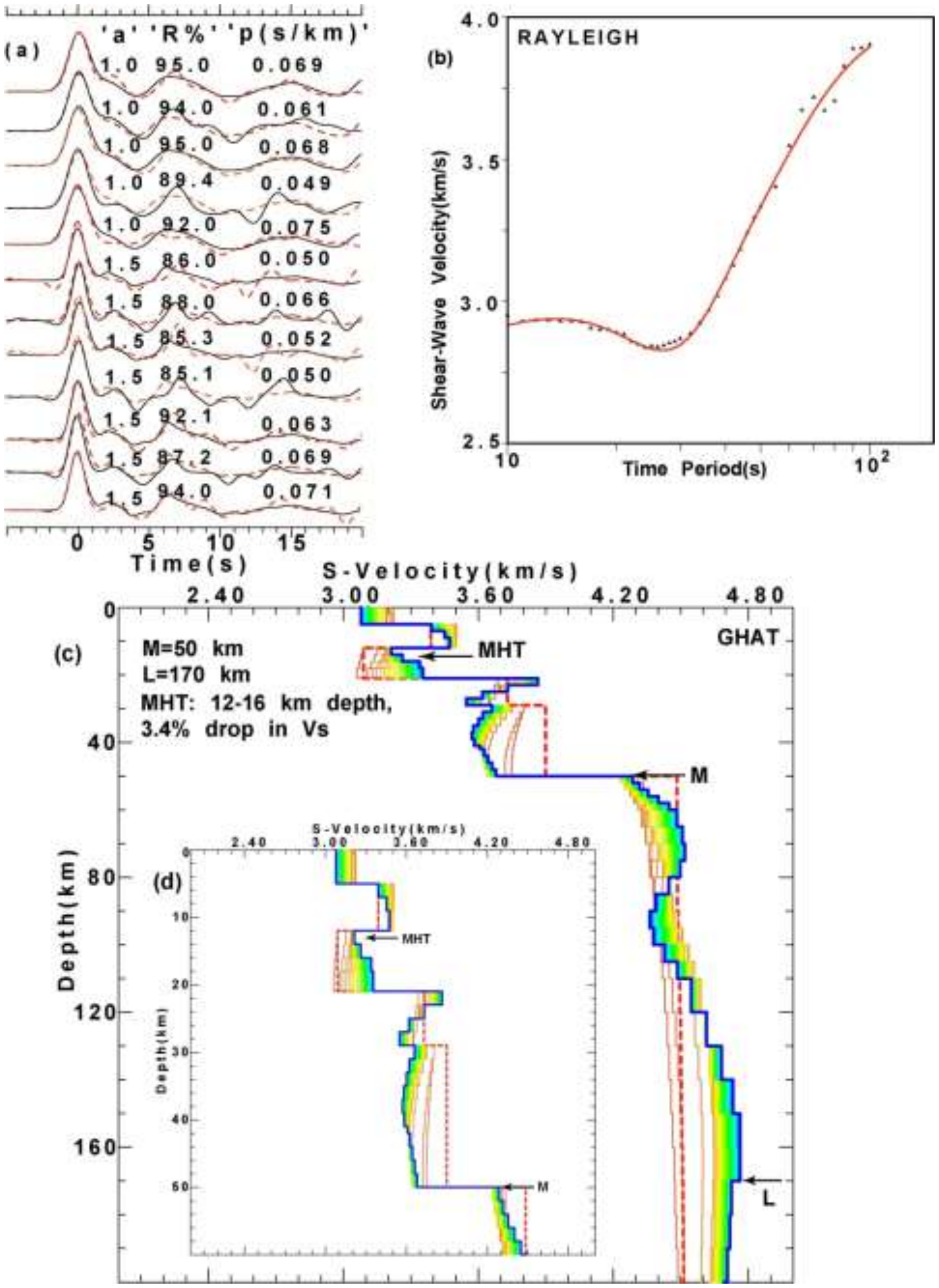


Fig S29: Results of joint inversion of P-RFs and fundamental mode surface wave group velocity dispersion (SWD) data at GHAT station, (a) showing good agreement between observed (black line) and inverted (red line) radial RFs with a=1.0, 1.5 and 2.0, for different horizontal slowness (S, in s/km). Here, "a" and "R%" represent Gaussian width factor (used for estimating RF) and agreement (in %) between observed and inverted RFs, respectively. Correlation between observed and inverted dispersion curves of (b) Rayleigh waves. (c) Inverted shear velocity models showing the main Himalayan thrust (MHT), Moho (M) and LAB (L) depth estimates in km. Different colours represent different Vs models used for the joint inversion. The initial shear velocity model is shown by a thick red dotted line, while the final shear velocity model is shown by a thick blue line and (d) A plot showing zoomed portion of the crustal part from the inverted Vs models.

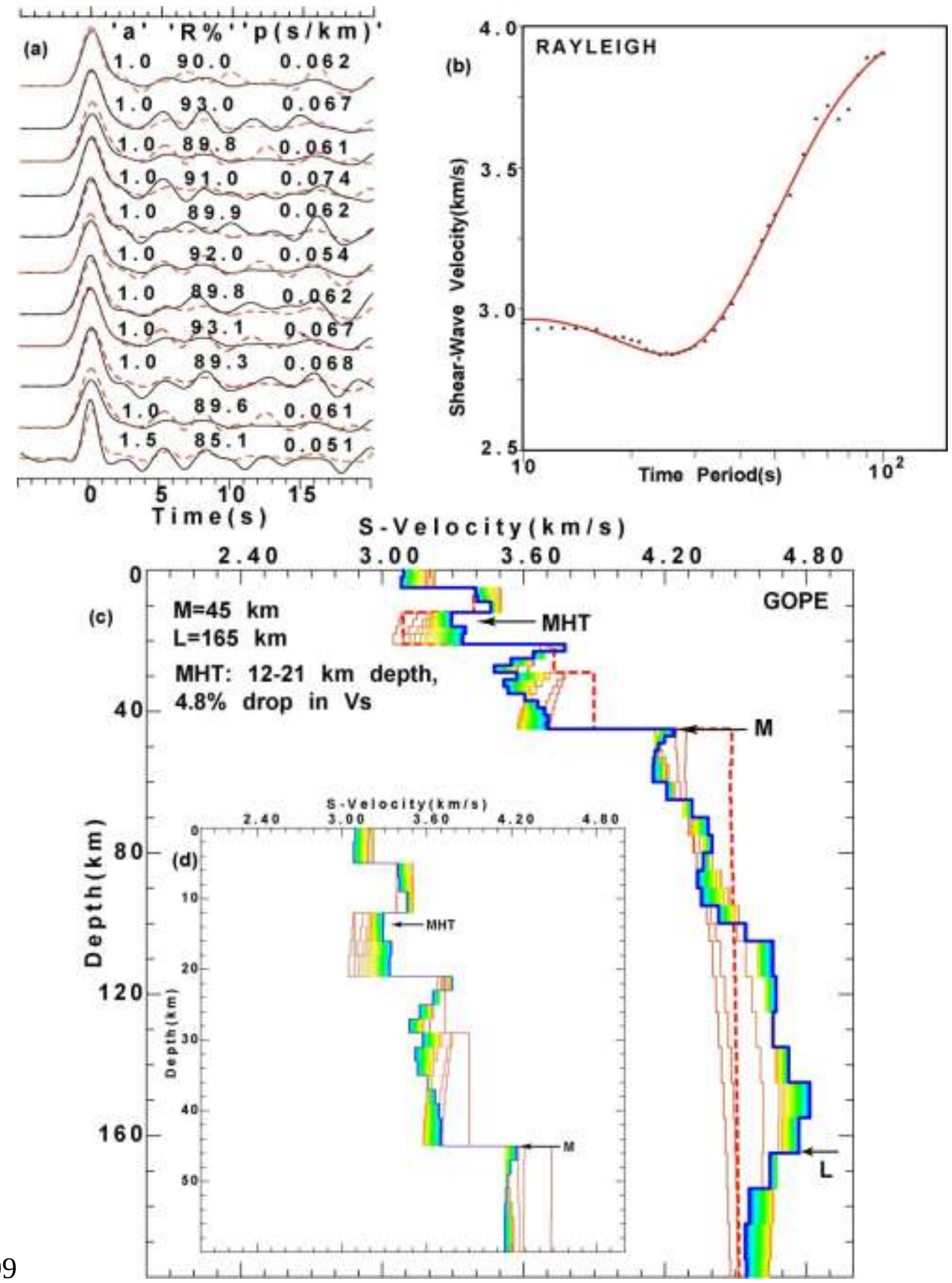


Fig S30: Results of joint inversion of P-RFs and fundamental mode surface wave group velocity dispersion (SWD) data at GOPE station, (a) showing good agreement between observed (black line) and inverted (red line) radial RFs with a=1.0, 1.5 and 2.0, for different horizontal slowness (S, in s/km). Here, "a" and "R%" represent Gaussian width factor (used for estimating RF) and agreement (in %) between observed and inverted RFs, respectively. Correlation between observed and inverted dispersion curves of (b) Rayleigh waves. (c) Inverted shear velocity models showing the main Himalayan thrust (MHT), Moho (M) and LAB (L) depth estimates in km. Different colours represent different Vs models used for the joint inversion. The initial shear velocity model is shown by a thick red dotted line, while the final shear velocity model is shown by a thick blue line and (d) A plot showing zoomed portion of the crustal part from the inverted Vs models.

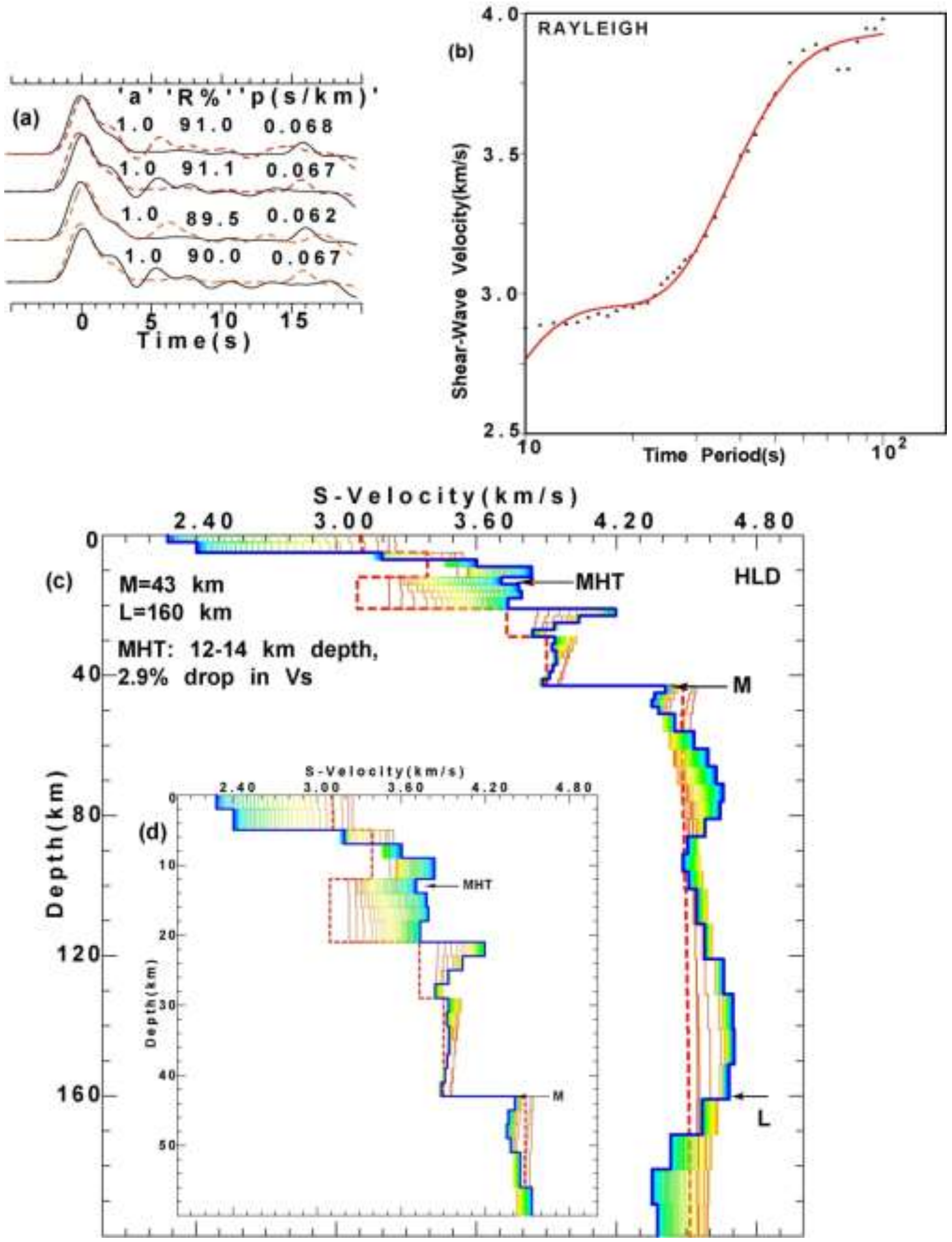


Fig S31: Results of joint inversion of P-RFs and fundamental mode surface wave group velocity dispersion (SWD) data at HLD station, (a) showing good agreement between observed (black line) and inverted (red line) radial RFs with a=1.0, 1.5 and 2.0, for different horizontal slowness (S, in s/km). Here, "a" and "R%" represent Gaussian width factor (used for estimating RF) and agreement (in %) between observed and inverted RFs, respectively. Correlation between observed and inverted dispersion curves of (b) Rayleigh waves. (c) Inverted shear velocity models showing the main Himalayan thrust (MHT), Moho (M) and LAB (L) depth estimates in km. Different colours represent different Vs models used for the joint inversion. The initial shear velocity model is shown by a thick red dotted line, while the final shear velocity model is shown by a thick blue line and (d) A plot showing zoomed portion of the crustal part from the inverted Vs models.

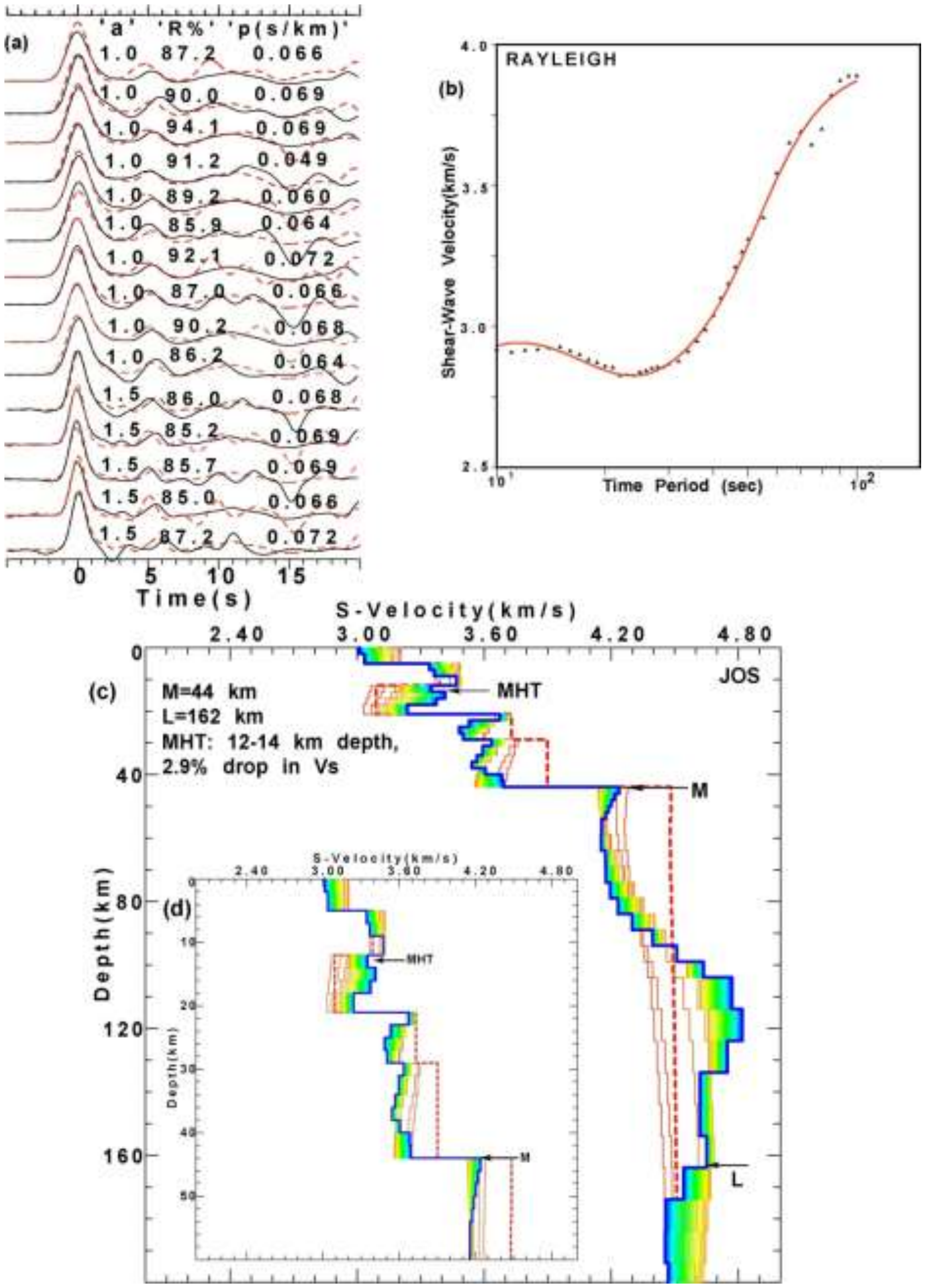


Fig S32: Results of joint inversion of P-RFs and fundamental mode surface wave group velocity dispersion (SWD) data at JOS station, (a) showing good agreement between observed (black line) and inverted (red line) radial RFs with a=1.0, 1.5 and 2.0, for different horizontal slowness (S, in s/km). Here, "a" and "R%" represent Gaussian width factor (used for estimating RF) and agreement (in %) between observed and inverted RFs, respectively. Correlation between observed and inverted dispersion curves of (b) Rayleigh waves. (c) Inverted shear velocity models showing the main Himalayan thrust (MHT), Moho (M) and LAB (L) depth estimates in km. Different colours represent different Vs models used for the joint inversion. The initial shear velocity model is shown by a thick red dotted line, while the final shear velocity model is shown by a thick blue line and (d) A plot showing zoomed portion of the crustal part from the inverted Vs models.

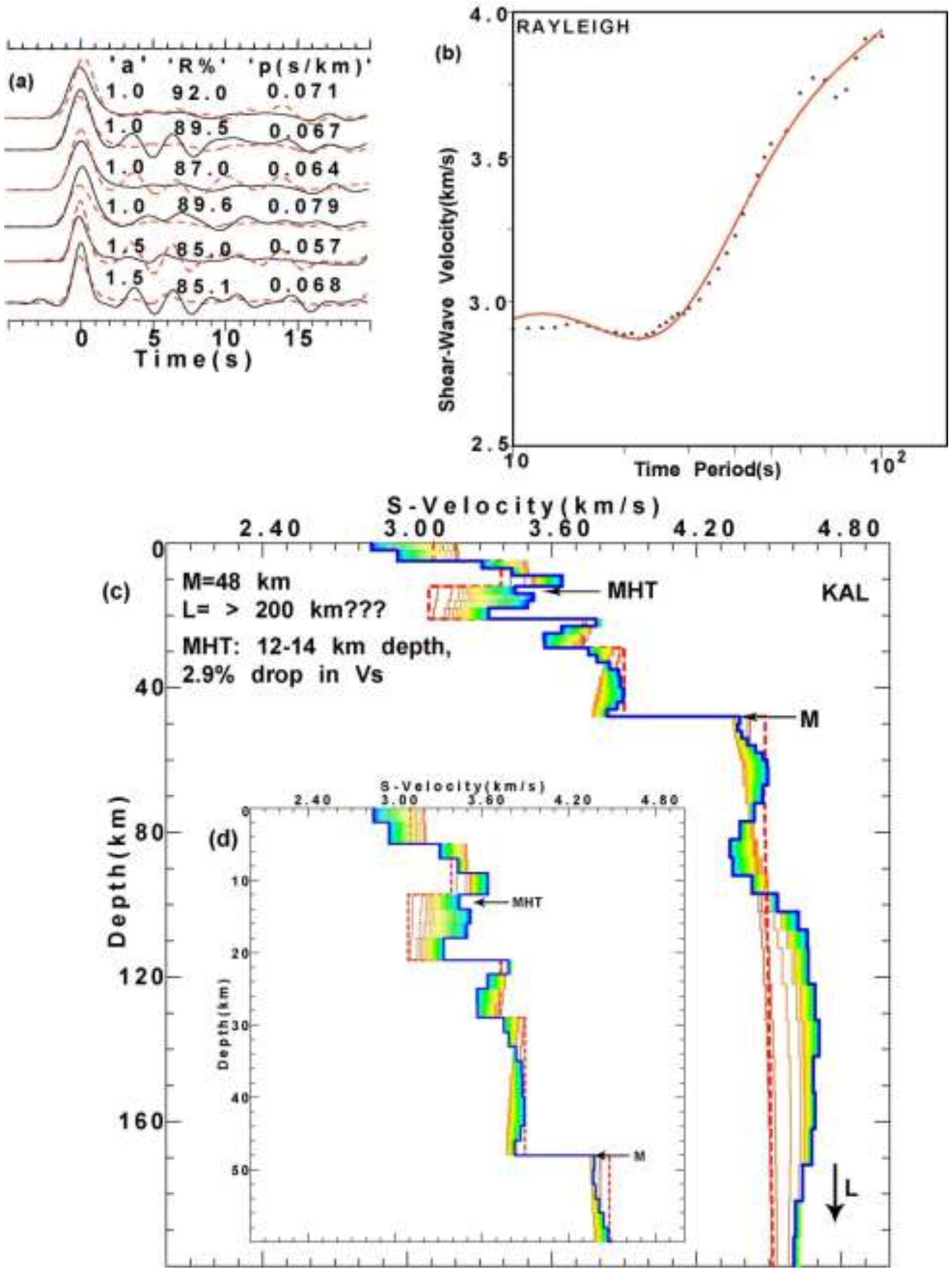


Fig S33: Results of joint inversion of P-RFs and fundamental mode surface wave group velocity dispersion (SWD) data at KAL station, (a) showing good agreement between observed (black line) and inverted (red line) radial RFs with a=1.0, 1.5 and 2.0, for different horizontal slowness (S, in s/km). Here, "a" and "R%" represent Gaussian width factor (used for estimating RF) and agreement (in %) between observed and inverted RFs, respectively. Correlation between observed and inverted dispersion curves of (b) Rayleigh waves. (c) Inverted shear velocity models showing the main Himalayan thrust (MHT), Moho (M) and LAB (L) depth estimates in km. Different colours represent different Vs models used for the joint inversion. The initial shear velocity model is shown by a thick red dotted line, while the final shear velocity model is shown by a thick blue line and (d) A plot showing zoomed portion of the crustal part from the inverted Vs models.

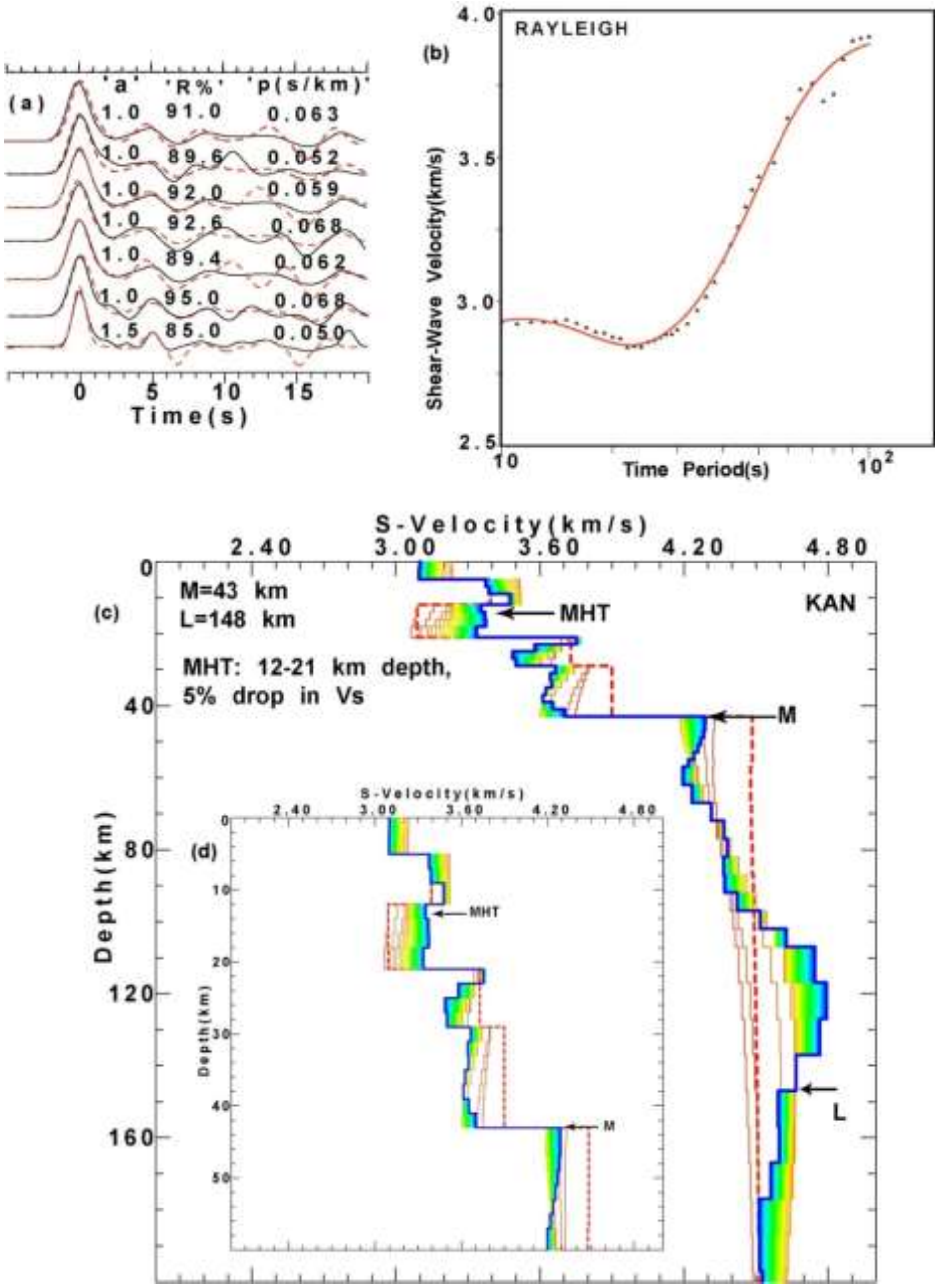


Fig S34: Results of joint inversion of P-RFs and fundamental mode surface wave group velocity dispersion (SWD) data at KAN station, (a) showing good agreement between observed (black line) and inverted (red line) radial RFs with a=1.0, 1.5 and 2.0, for different horizontal slowness (S, in s/km). Here, "a" and "R%" represent Gaussian width factor (used for estimating RF) and agreement (in %) between observed and inverted RFs, respectively. Correlation between observed and inverted dispersion curves of (b) Rayleigh waves. (c) Inverted shear velocity models showing the main Himalayan thrust (MHT), Moho (M) and LAB (L) depth estimates in km. Different colours represent different Vs models used for the joint inversion. The initial shear velocity model is shown by a thick red dotted line, while the final shear velocity model is shown by a thick blue line and (d) A plot showing zoomed portion of the crustal part from the inverted Vs models.

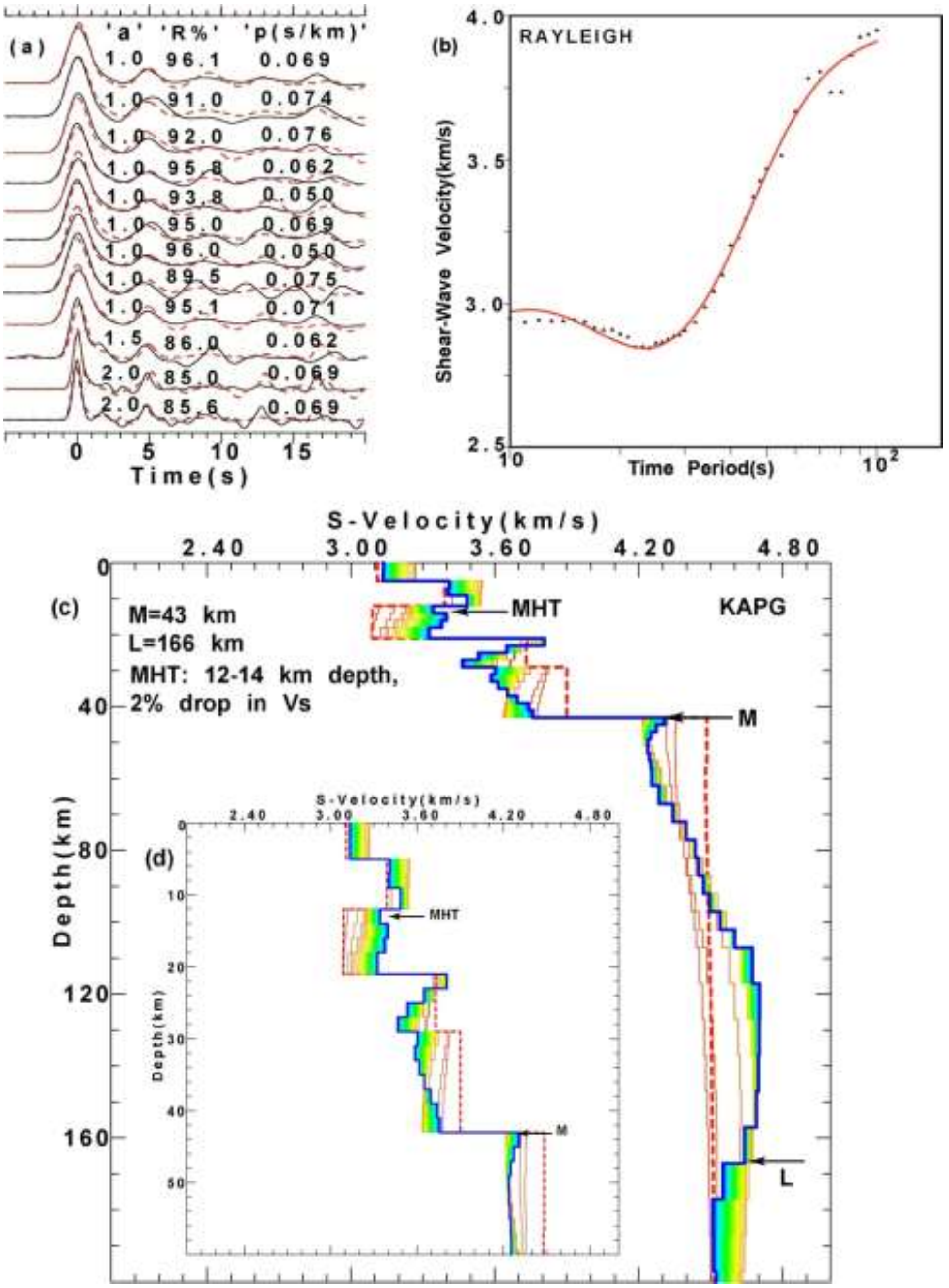


Fig S35: Results of joint inversion of P-RFs and fundamental mode surface wave group velocity dispersion (SWD) data at KAPG station, (a) showing good agreement between observed (black line) and inverted (red line) radial RFs with a=1.0, 1.5 and 2.0, for different horizontal slowness (S, in s/km). Here, "a" and "R%" represent Gaussian width factor (used for estimating RF) and agreement (in %) between observed and inverted RFs, respectively. Correlation between observed and inverted dispersion curves of (b) Rayleigh waves. (c) Inverted shear velocity models showing the main Himalayan thrust (MHT), Moho (M) and LAB (L) depth estimates in km. Different colours represent different Vs models used for the joint inversion. The initial shear velocity model is shown by a thick red dotted line, while the final shear velocity model is shown by a thick blue line and (d) A plot showing zoomed portion of the crustal part from the inverted Vs models.

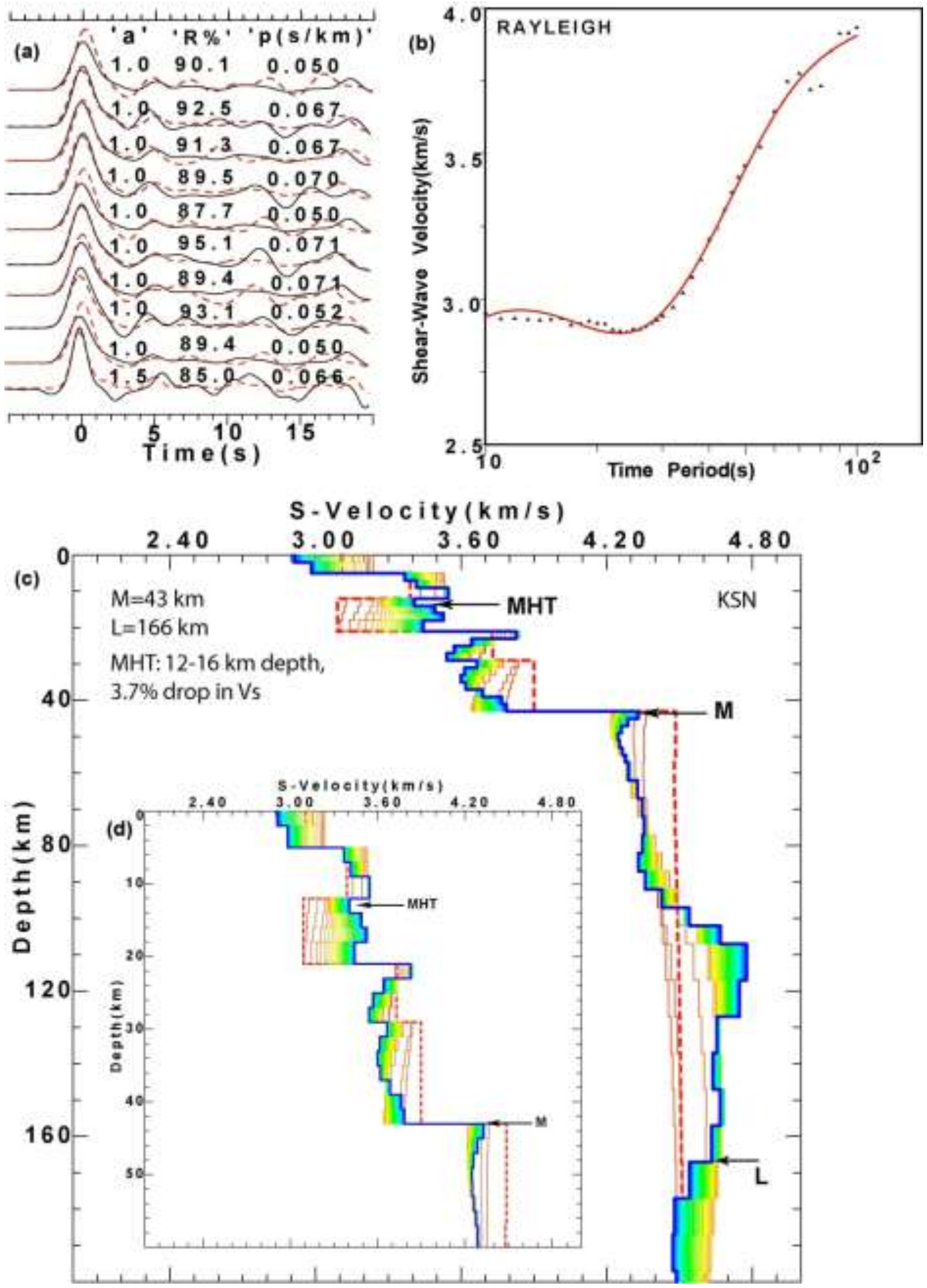


Fig S36: Results of joint inversion of P-RFs and fundamental mode surface wave group velocity dispersion (SWD) data at KSN station, (a) showing good agreement between observed (black line) and inverted (red line) radial RFs with a=1.0, 1.5 and 2.0, for different horizontal slowness (S, in s/km). Here, "a" and "R%" represent Gaussian width factor (used for estimating RF) and agreement (in %) between observed and inverted RFs, respectively. Correlation between observed and inverted dispersion curves of (b) Rayleigh waves. (c) Inverted shear velocity models showing the main Himalayan thrust (MHT), Moho (M) and LAB (L) depth estimates in km. Different colours represent different Vs models used for the joint inversion. The initial shear velocity model is shown by a thick red dotted line, while the final shear velocity model is shown by a thick blue line and (d) A plot showing zoomed portion of the crustal part from the inverted Vs models.

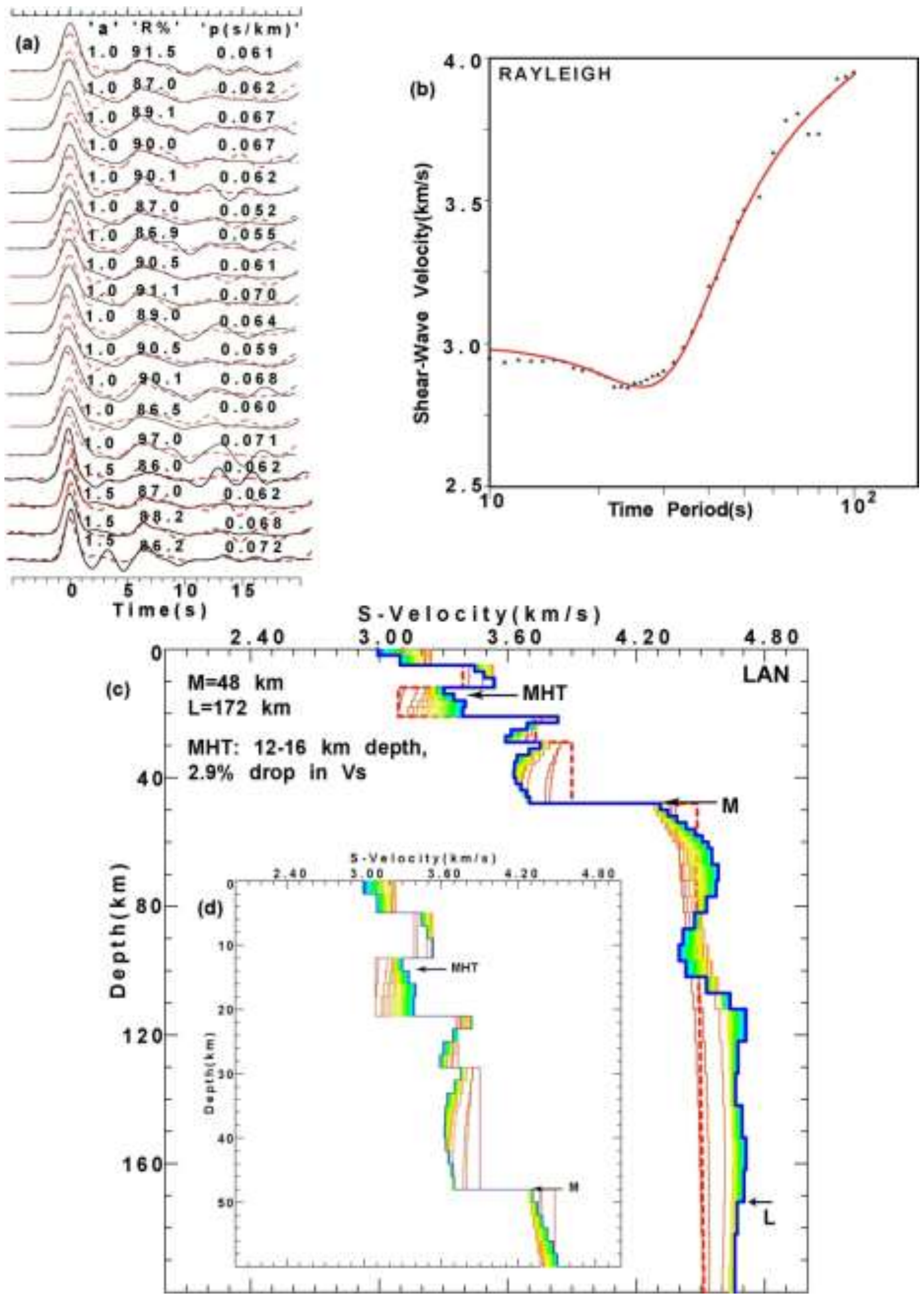


Fig S37: Results of joint inversion of P-RFs and fundamental mode surface wave group velocity dispersion (SWD) data at LAN station, (a) showing good agreement between observed (black line) and inverted (red line) radial RFs with a=1.0, 1.5 and 2.0, for different horizontal slowness (S, in s/km). Here, "a" and "R%" represent Gaussian width factor (used for estimating RF) and agreement (in %) between observed and inverted RFs, respectively. Correlation between observed and inverted dispersion curves of (b) Rayleigh waves. (c) Inverted shear velocity models showing the main Himalayan thrust (MHT), Moho (M) and LAB (L) depth estimates in km. Different colours represent different Vs models used for the joint inversion. The initial shear velocity model is shown by a thick red dotted line, while the final shear velocity model is shown by a thick blue line and (d) A plot showing zoomed portion of the crustal part from the inverted Vs models.

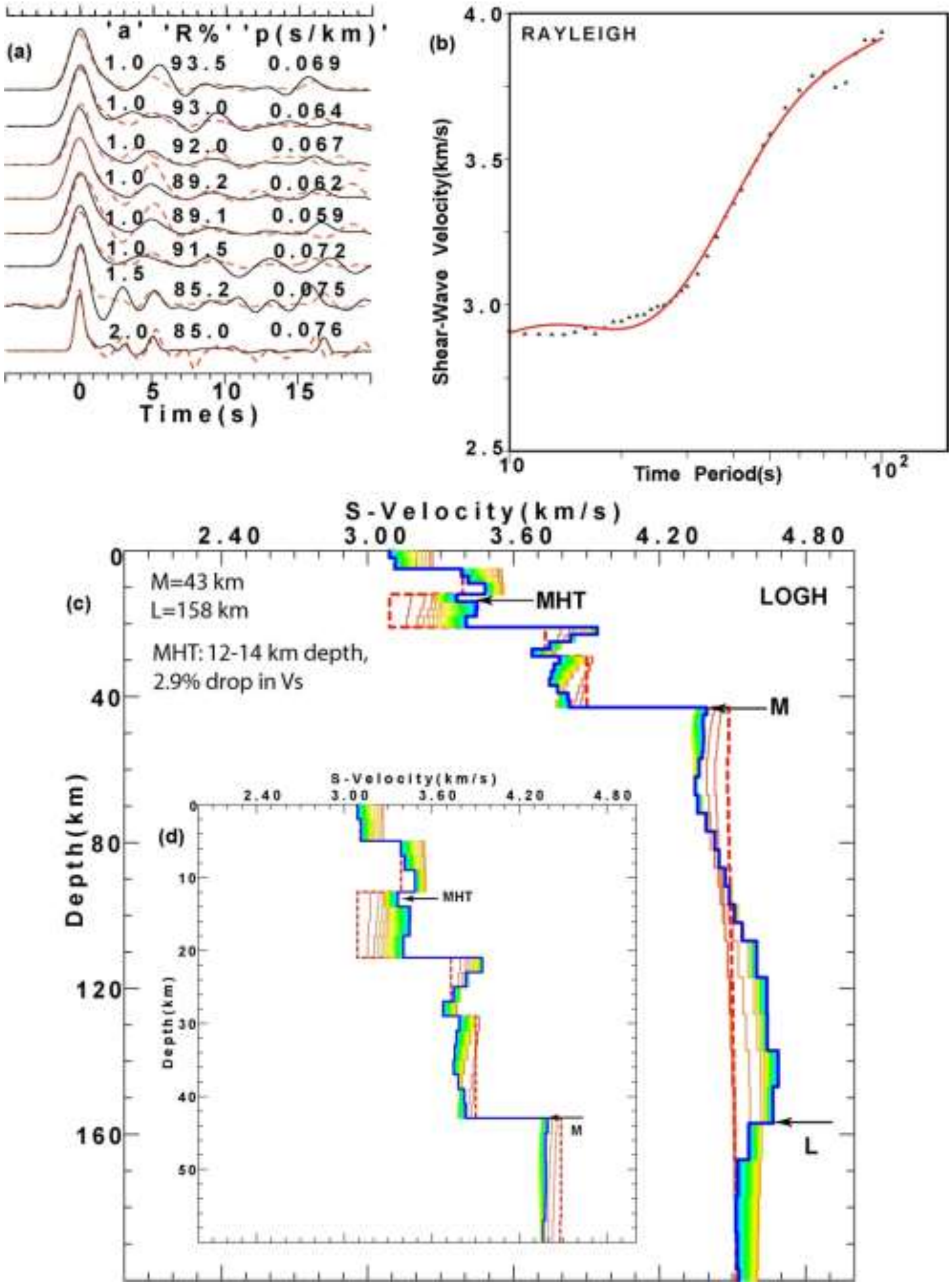


Fig S38: Results of joint inversion of P-RFs and fundamental mode surface wave group velocity dispersion (SWD) data at LOGH station, (a) showing good agreement between observed (black line) and inverted (red line) radial RFs with a=1.0, 1.5 and 2.0, for different horizontal slowness (S, in s/km). Here, "a" and "R%" represent Gaussian width factor (used for estimating RF) and agreement (in %) between observed and inverted RFs, respectively. Correlation between observed and inverted dispersion curves of (b) Rayleigh waves. (c) Inverted shear velocity models showing the main Himalayan thrust (MHT), Moho (M) and LAB (L) depth estimates in km. Different colours represent different Vs models used for the joint inversion. The initial shear velocity model is shown by a thick red dotted line, while the final shear velocity model is shown by a thick blue line and (d) A plot showing zoomed portion of the crustal part from the inverted Vs models.

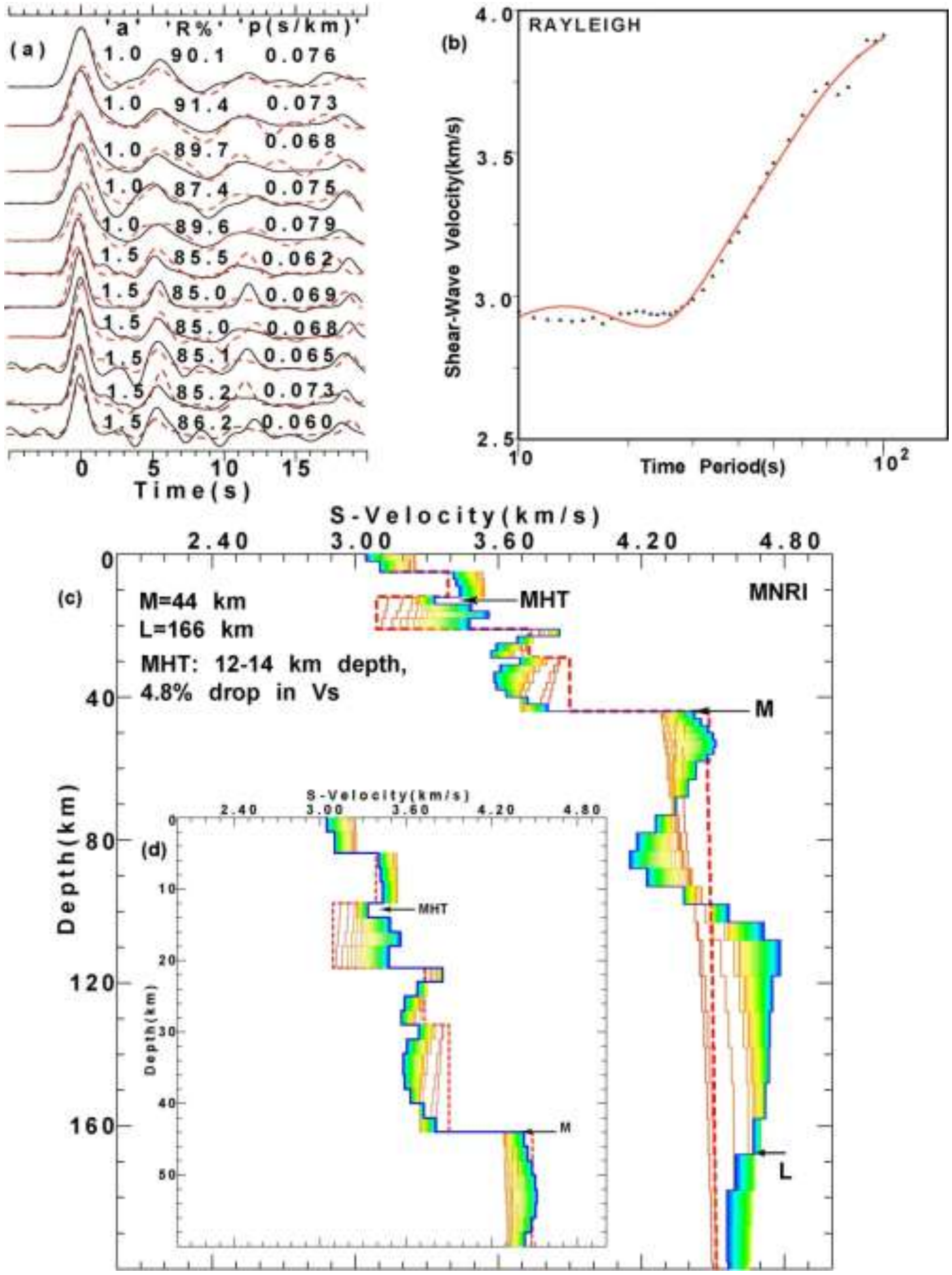


Fig S39: Results of joint inversion of P-RFs and fundamental mode surface wave group velocity dispersion (SWD) data at MNRI station, (a) showing good agreement between observed (black line) and inverted (red line) radial RFs with a=1.0, 1.5 and 2.0, for different horizontal slowness (S, in s/km). Here, "a" and "R%" represent Gaussian width factor (used for estimating RF) and agreement (in %) between observed and inverted RFs, respectively. Correlation between observed and inverted dispersion curves of (b) Rayleigh waves. (c) Inverted shear velocity models showing the main Himalayan thrust (MHT), Moho (M) and LAB (L) depth estimates in km. Different colours represent different Vs models used for the joint inversion. The initial shear velocity model is shown by a thick red dotted line, while the final shear velocity model is shown by a thick blue line and (d) A plot showing zoomed portion of the crustal part from the inverted Vs models.

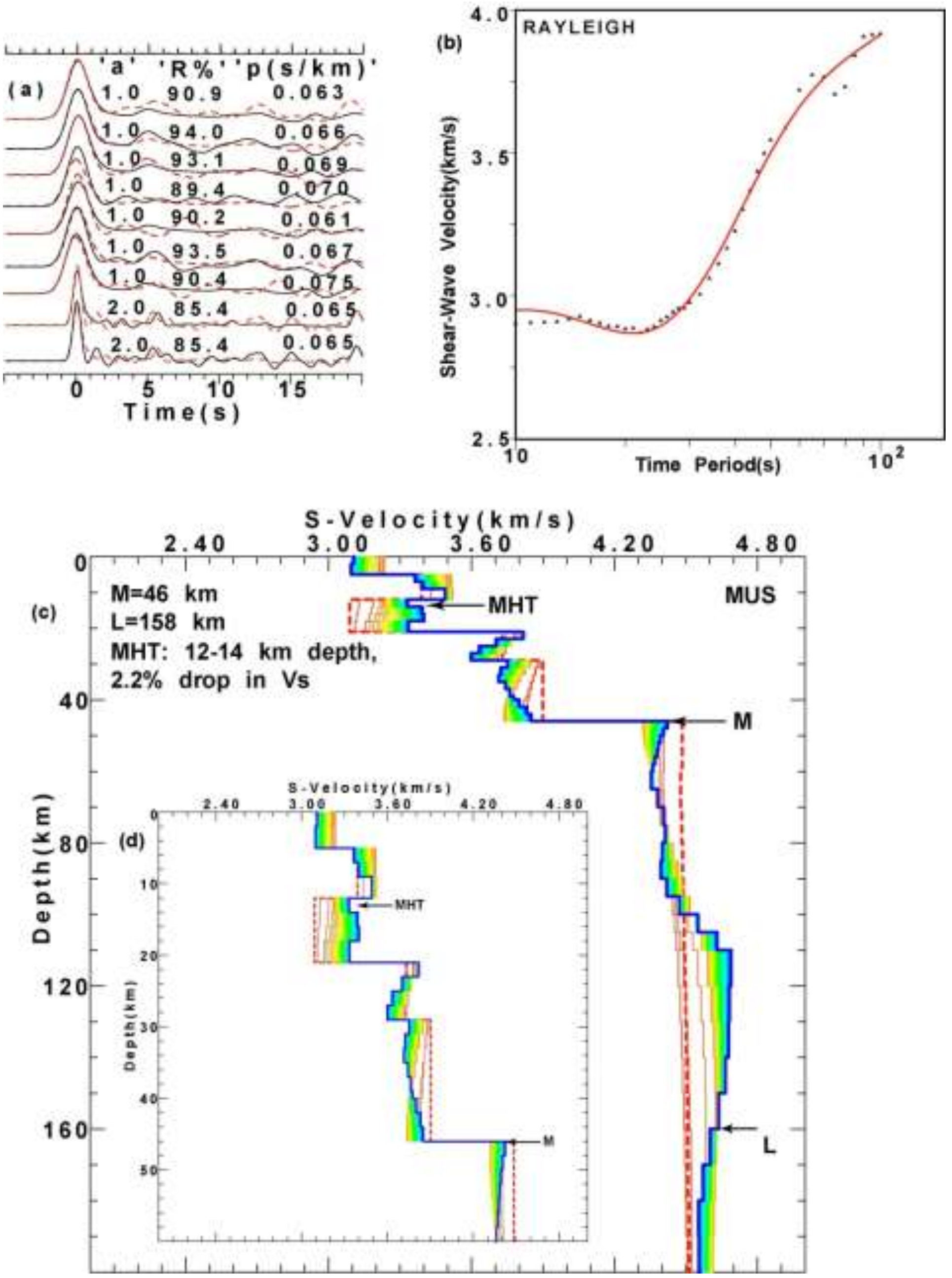


Fig S40: Results of joint inversion of P-RFs and fundamental mode surface wave group velocity dispersion (SWD) data at MUS station, (a) showing good agreement between observed (black line) and inverted (red line) radial RFs with a=1.0, 1.5 and 2.0, for different horizontal slowness (S, in s/km). Here, "a" and "R%" represent Gaussian width factor (used for estimating RF) and agreement (in %) between observed and inverted RFs, respectively. Correlation between observed and inverted dispersion curves of (b) Rayleigh waves. (c) Inverted shear velocity models showing the main Himalayan thrust (MHT), Moho (M) and LAB (L) depth estimates in km. Different colours represent different Vs models used for the joint inversion. The initial shear velocity model is shown by a thick red dotted line, while the final shear velocity model is shown by a thick blue line and (d) A plot showing zoomed portion of the crustal part from the inverted Vs models.

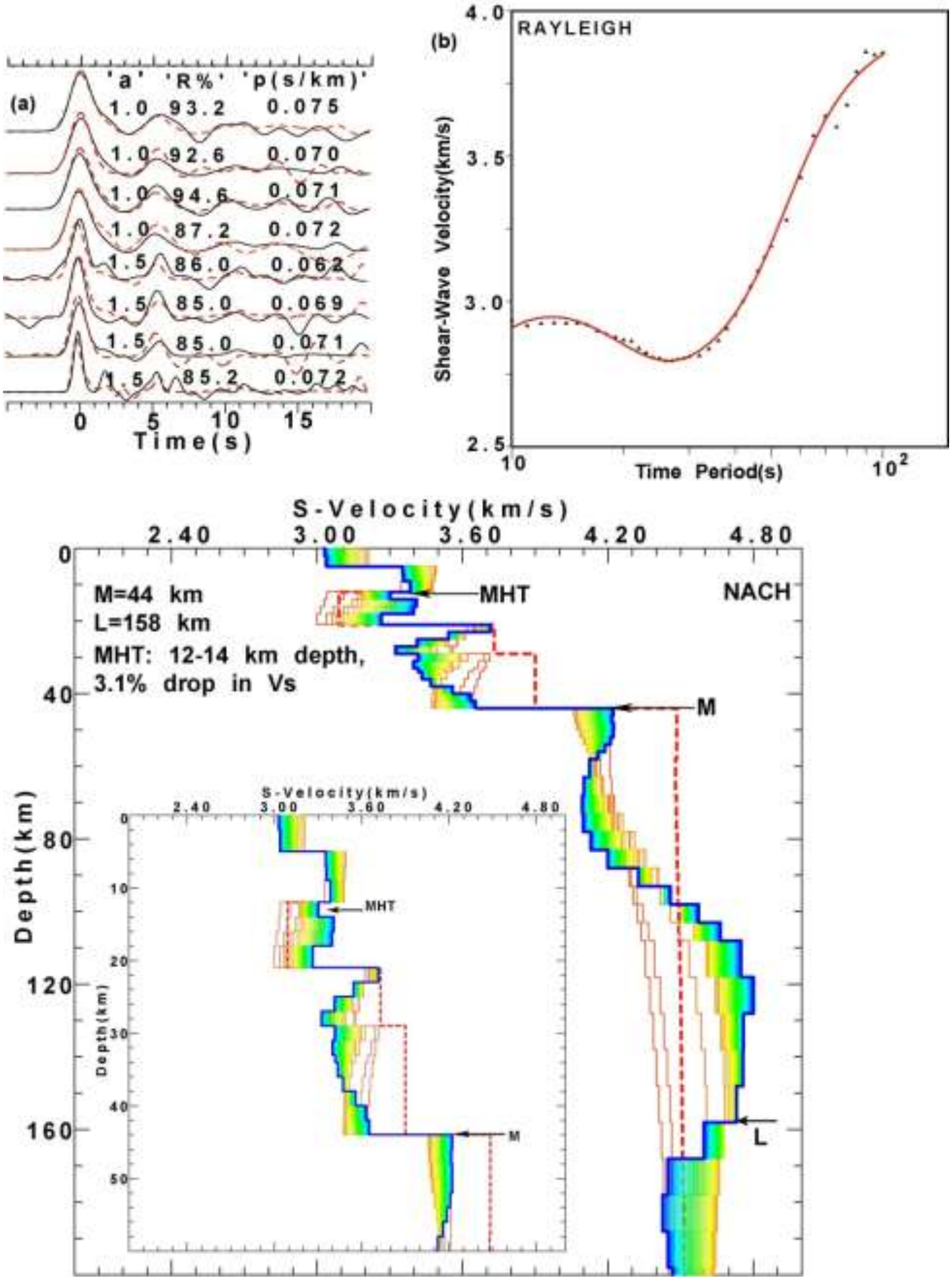


Fig S41: Results of joint inversion of P-RFs and fundamental mode surface wave group velocity dispersion (SWD) data at NACH station, (a) showing good agreement between observed (black line) and inverted (red line) radial RFs with a=1.0, 1.5 and 2.0, for different horizontal slowness (S, in s/km). Here, "a" and "R%" represent Gaussian width factor (used for estimating RF) and agreement (in %) between observed and inverted RFs, respectively. Correlation between observed and inverted dispersion curves of (b) Rayleigh waves. (c) Inverted shear velocity models showing the main Himalayan thrust (MHT), Moho (M) and LAB (L) depth estimates in km. Different colours represent different Vs models used for the joint inversion. The initial shear velocity model is shown by a thick red dotted line, while the final shear velocity model is shown by a thick blue line and (d) A plot showing zoomed portion of the crustal part from the inverted Vs models.

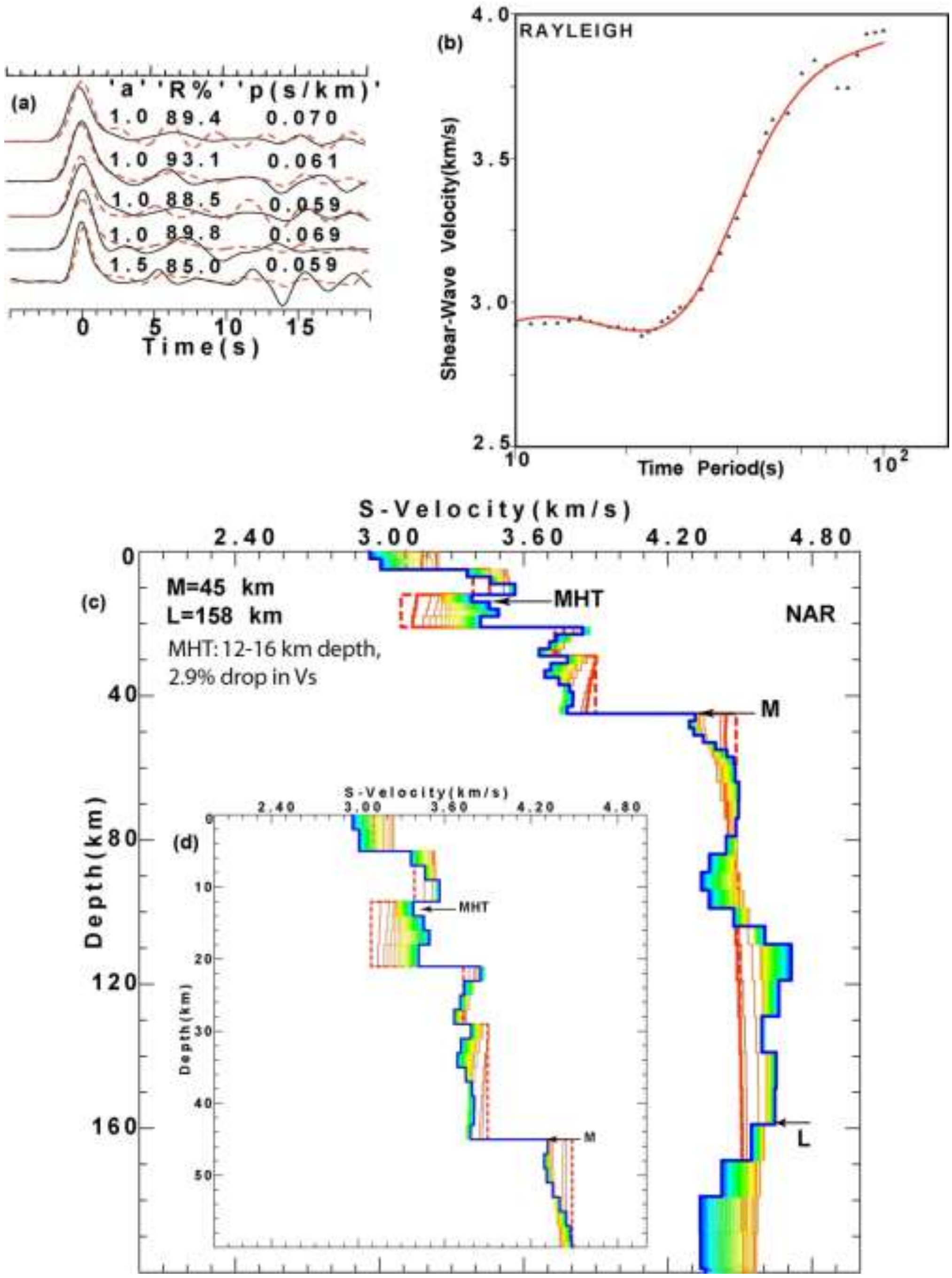


Fig S42: Results of joint inversion of P-RFs and fundamental mode surface wave group velocity dispersion (SWD) data at NAR station, (a) showing good agreement between observed (black line) and inverted (red line) radial RFs with a=1.0, 1.5 and 2.0, for different horizontal slowness (S, in s/km). Here, "a" and "R%" represent Gaussian width factor (used for estimating RF) and agreement (in %) between observed and inverted RFs, respectively. Correlation between observed and inverted dispersion curves of (b) Rayleigh waves. (c) Inverted shear velocity models showing the main Himalayan thrust (MHT), Moho (M) and LAB (L) depth estimates in km. Different colours represent different Vs models used for the joint inversion. The initial shear velocity model is shown by a thick red dotted line, while the final shear velocity model is shown by a thick blue line and (d) A plot showing zoomed portion of the crustal part from the inverted Vs models.

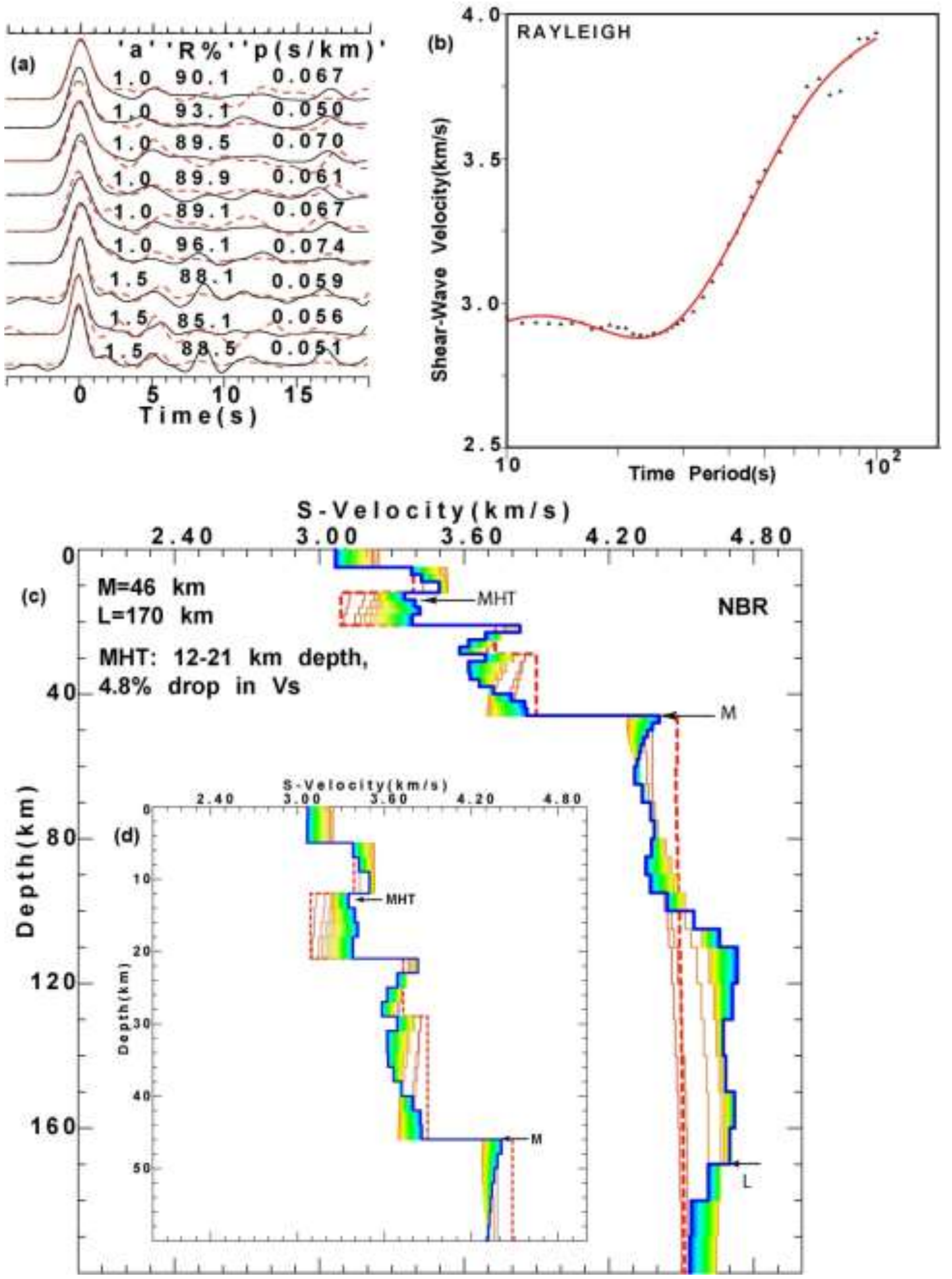


Fig S43: Results of joint inversion of P-RFs and fundamental mode surface wave group velocity dispersion (SWD) data at NBR station, (a) showing good agreement between observed (black line) and inverted (red line) radial RFs with a=1.0, 1.5 and 2.0, for different horizontal slowness (S, in s/km). Here, "a" and "R%" represent Gaussian width factor (used for estimating RF) and agreement (in %) between observed and inverted RFs, respectively. Correlation between observed and inverted dispersion curves of (b) Rayleigh waves. (c) Inverted shear velocity models showing the main Himalayan thrust (MHT), Moho (M) and LAB (L) depth estimates in km. Different colours represent different Vs models used for the joint inversion. The initial shear velocity model is shown by a thick red dotted line, while the final shear velocity model is shown by a thick blue line and (d) A plot showing zoomed portion of the crustal part from the inverted Vs models.

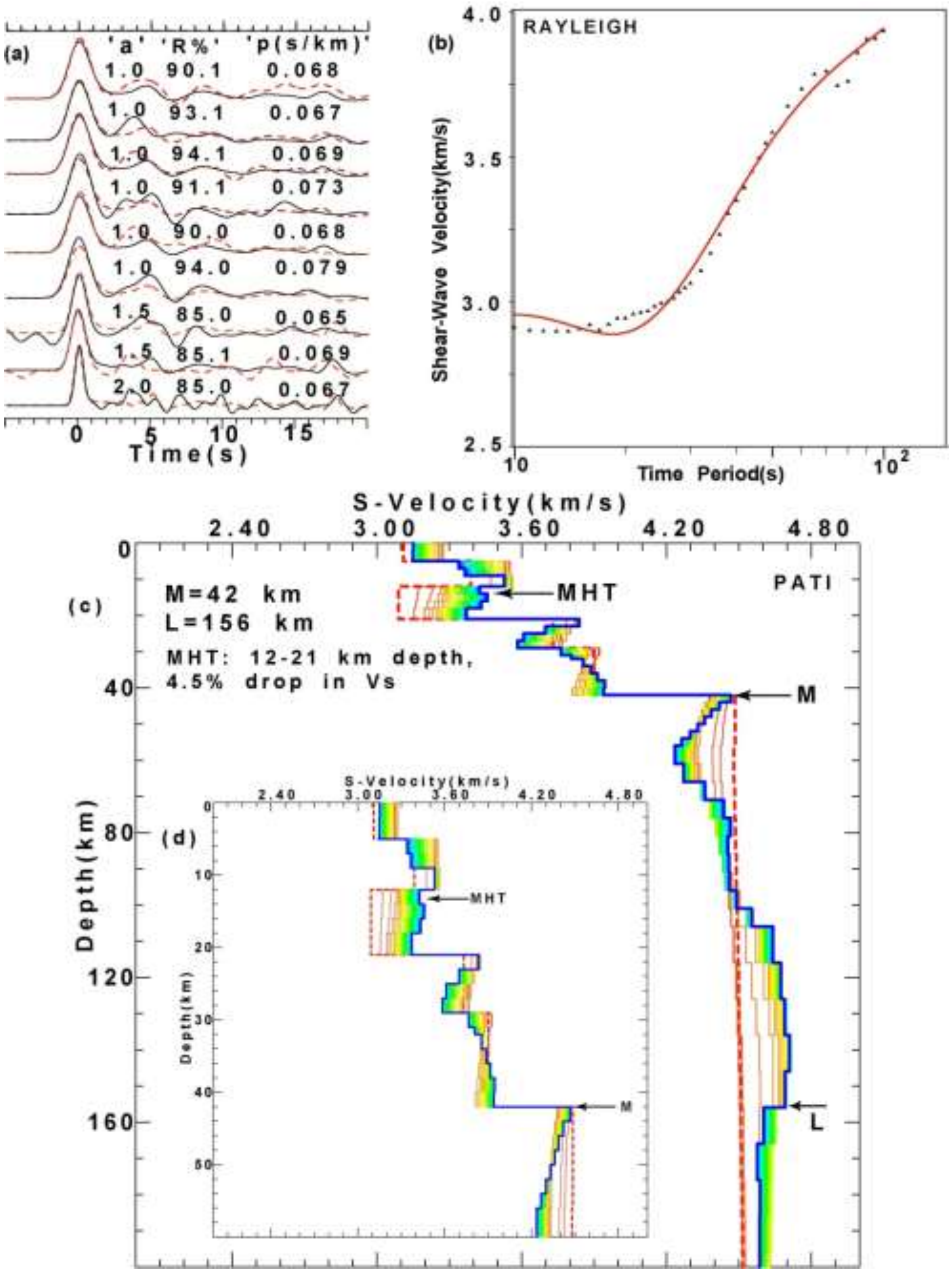


Fig S44: Results of joint inversion of P-RFs and fundamental mode surface wave group velocity dispersion (SWD) data at PATI station, (a) showing good agreement between observed (black line) and inverted (red line) radial RFs with a=1.0, 1.5 and 2.0, for different horizontal slowness (S, in s/km). Here, "a" and "R%" represent Gaussian width factor (used for estimating RF) and agreement (in %) between observed and inverted RFs, respectively. Correlation between observed and inverted dispersion curves of (b) Rayleigh waves. (c) Inverted shear velocity models showing the main Himalayan thrust (MHT), Moho (M) and LAB (L) depth estimates in km. Different colours represent different Vs models used for the joint inversion. The initial shear velocity model is shown by a thick red dotted line, while the final shear velocity model is shown by a thick blue line and (d) A plot showing zoomed portion of the crustal part from the inverted Vs models.

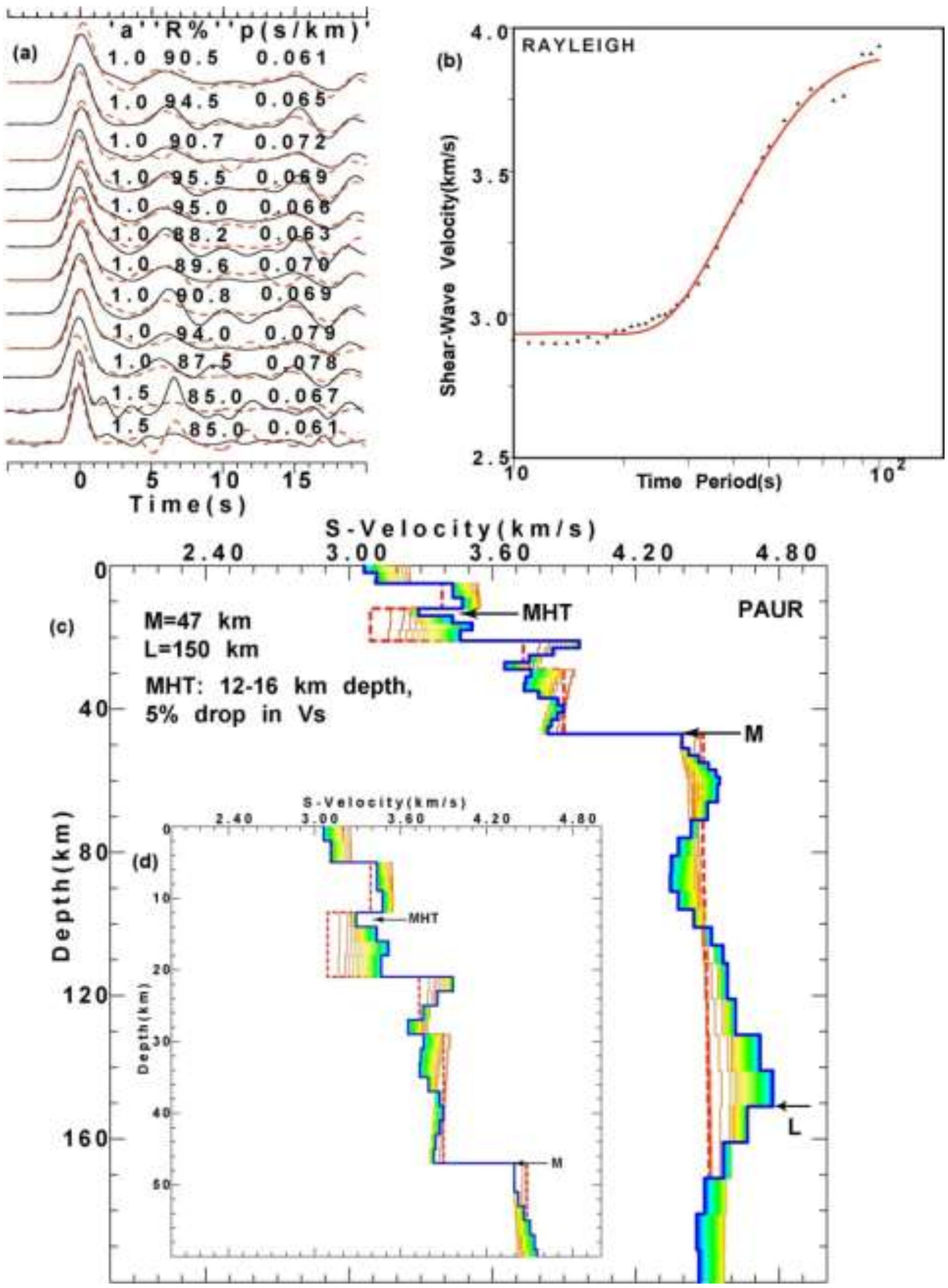


Fig S45: Results of joint inversion of P-RFs and fundamental mode surface wave group velocity dispersion (SWD) data at PAUR station, (a) showing good agreement between observed (black line) and inverted (red line) radial RFs with a=1.0, 1.5 and 2.0, for different horizontal slowness (S, in s/km). Here, "a" and "R%" represent Gaussian width factor (used for estimating RF) and agreement (in %) between observed and inverted RFs, respectively. Correlation between observed and inverted dispersion curves of (b) Rayleigh waves. (c) Inverted shear velocity models showing the main Himalayan thrust (MHT), Moho (M) and LAB (L) depth estimates in km. Different colours represent different Vs models used for the joint inversion. The initial shear velocity model is shown by a thick red dotted line, while the final shear velocity model is shown by a thick blue line and (d) A plot showing zoomed portion of the crustal part from the inverted Vs models.

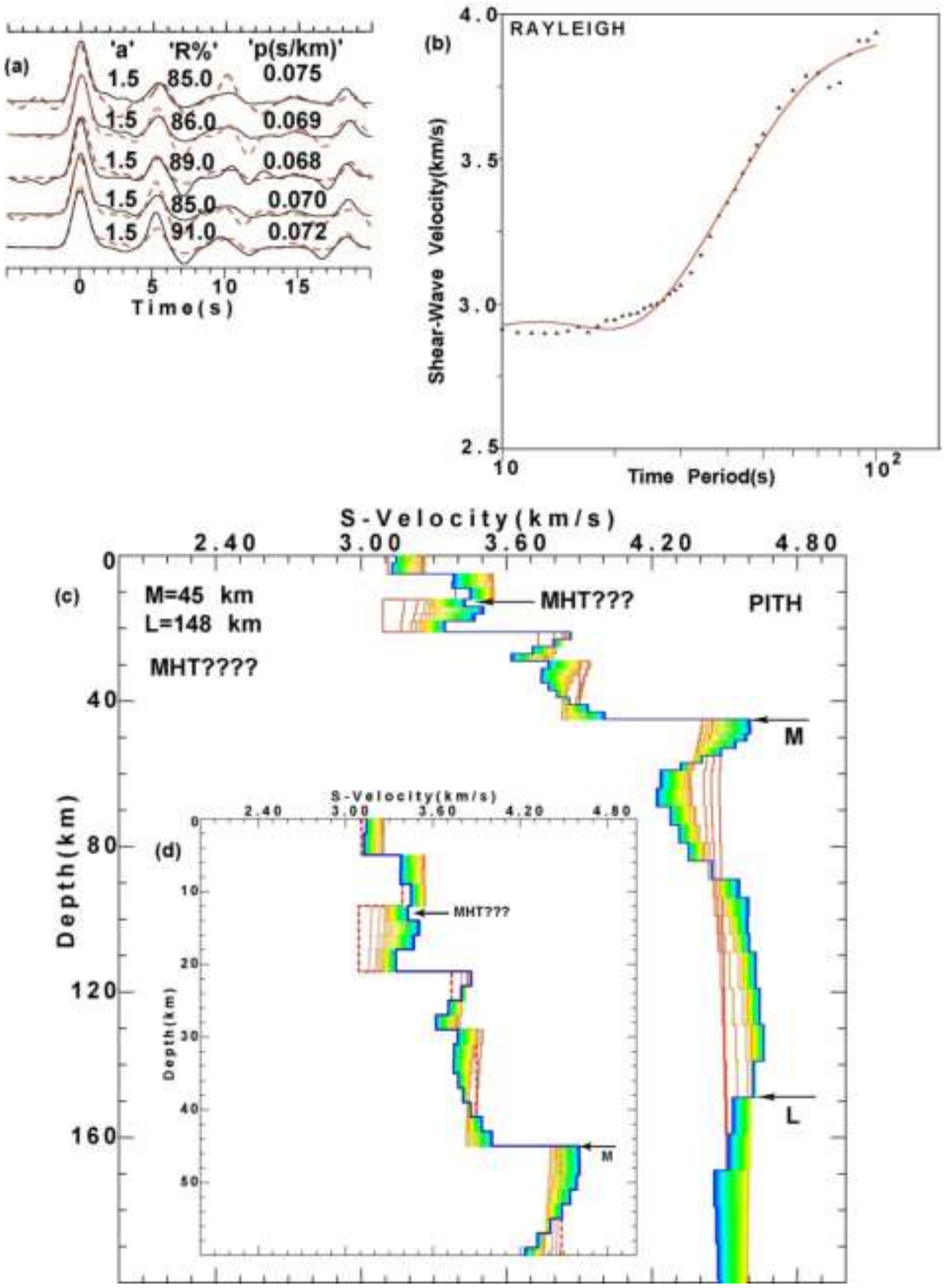


Fig S46: Results of joint inversion of P-RFs and fundamental mode surface wave group velocity dispersion (SWD) data at PITH station, (a) showing good agreement between observed (black line) and inverted (red line) radial RFs with a=1.0, 1.5 and 2.0, for different horizontal slowness (S, in s/km). Here, "a" and "R%" represent Gaussian width factor (used for estimating RF) and agreement (in %) between observed and inverted RFs, respectively. Correlation between observed and inverted dispersion curves of (b) Rayleigh waves. (c) Inverted shear velocity models showing the main Himalayan thrust (MHT), Moho (M) and LAB (L) depth estimates in km. Different colours represent different Vs models used for the joint inversion. The initial shear velocity model is shown by a thick red dotted line, while the final shear velocity model is shown by a thick blue line and (d) A plot showing zoomed portion of the crustal part from the inverted Vs models.

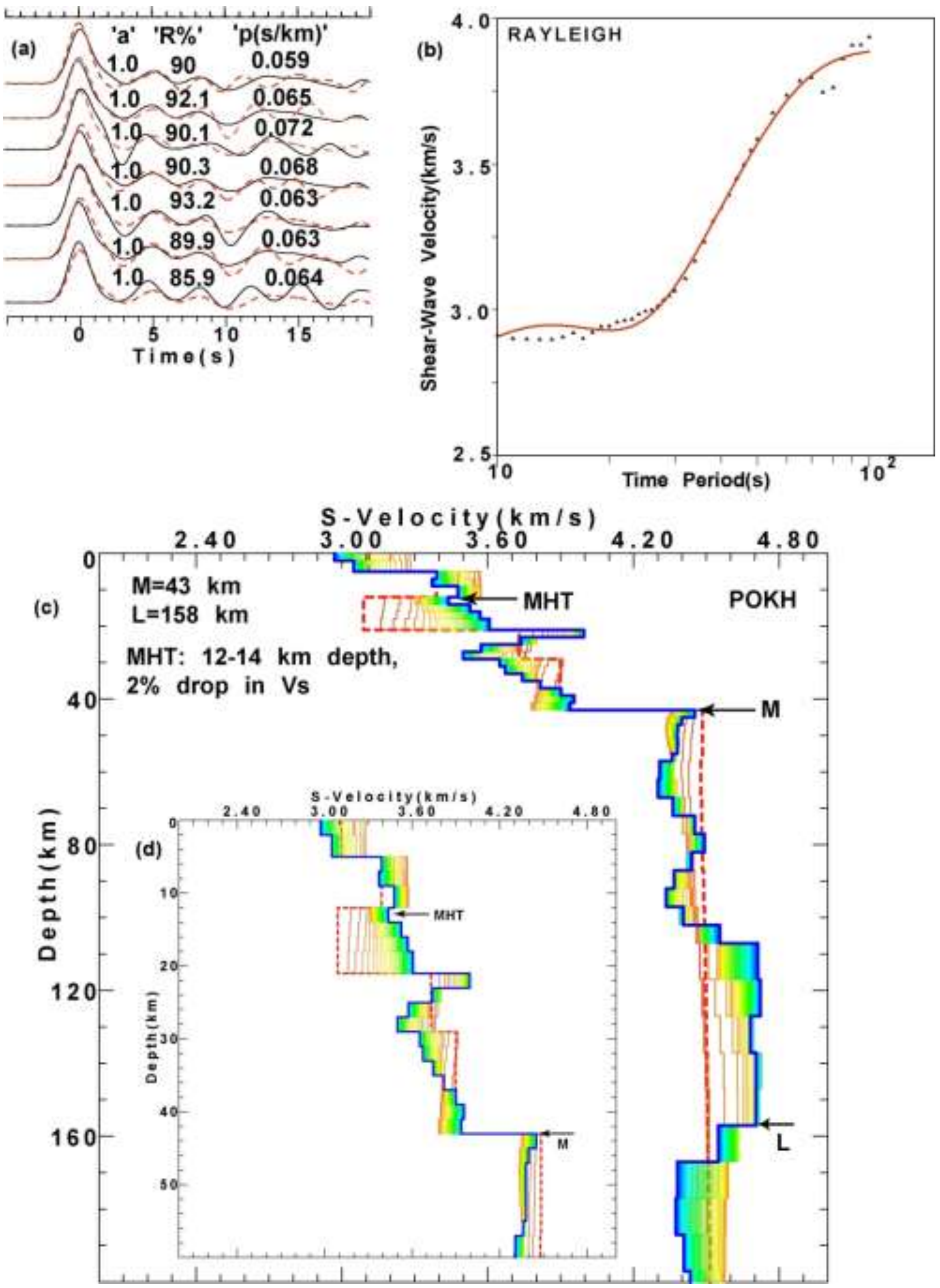


Fig S47: Results of joint inversion of P-RFs and fundamental mode surface wave group velocity dispersion (SWD) data at POKH station, (a) showing good agreement between observed (black line) and inverted (red line) radial RFs with a=1.0, 1.5 and 2.0, for different horizontal slowness (S, in s/km). Here, "a" and "R%" represent Gaussian width factor (used for estimating RF) and agreement (in %) between observed and inverted RFs, respectively. Correlation between observed and inverted dispersion curves of (b) Rayleigh waves. (c) Inverted shear velocity models showing the main Himalayan thrust (MHT), Moho (M) and LAB (L) depth estimates in km. Different colours represent different Vs models used for the joint inversion. The initial shear velocity model is shown by a thick red dotted line, while the final shear velocity model is shown by a thick blue line and (d) A plot showing zoomed portion of the crustal part from the inverted Vs models.

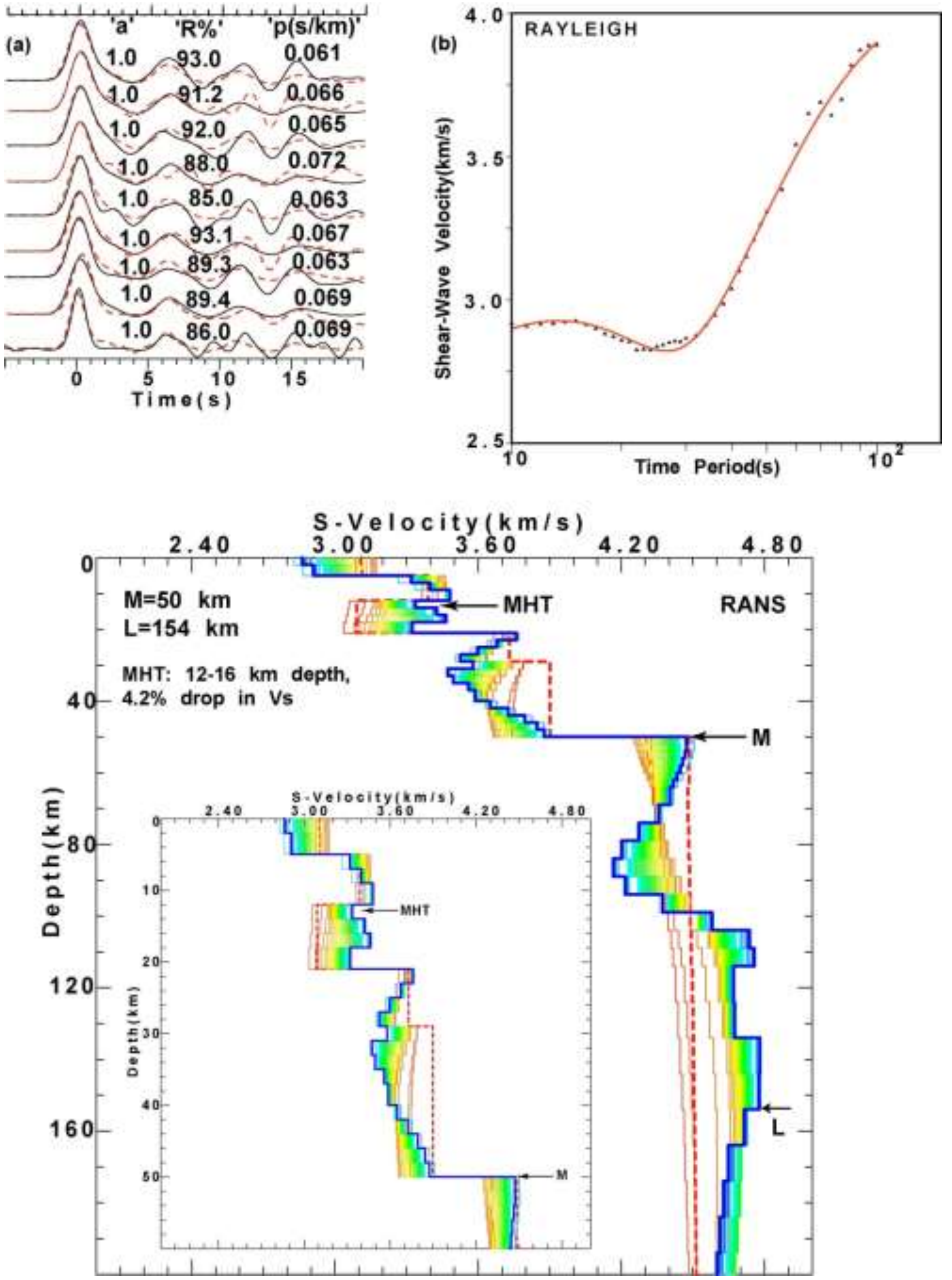


Fig S48: Results of joint inversion of P-RFs and fundamental mode surface wave group velocity dispersion (SWD) data at RANS station, (a) showing good agreement between observed (black line) and inverted (red line) radial RFs with a=1.0, 1.5 and 2.0, for different horizontal slowness (S, in s/km). Here, "a" and "R%" represent Gaussian width factor (used for estimating RF) and agreement (in %) between observed and inverted RFs, respectively. Correlation between observed and inverted dispersion curves of (b) Rayleigh waves. (c) Inverted shear velocity models showing the main Himalayan thrust (MHT), Moho (M) and LAB (L) depth estimates in km. Different colours represent different Vs models used for the joint inversion. The initial shear velocity model is shown by a thick red dotted line, while the final shear velocity model is shown by a thick blue line and (d) A plot showing zoomed portion of the crustal part from the inverted Vs models.

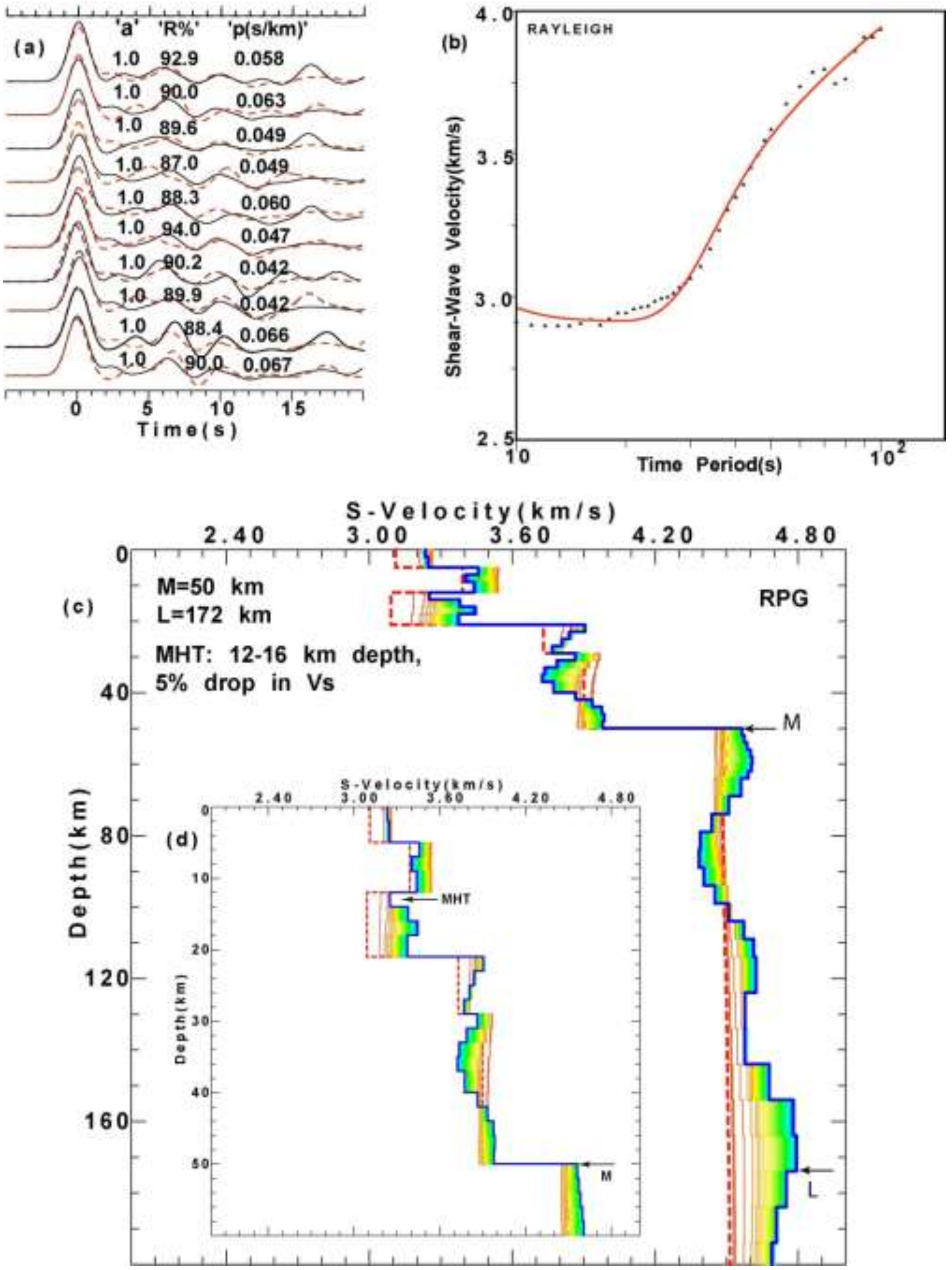


Fig S49: Results of joint inversion of P-RFs and fundamental mode surface wave group velocity dispersion (SWD) data at RPG station, (a) showing good agreement between observed (black line) and inverted (red line) radial RFs with a=1.0, 1.5 and 2.0, for different horizontal slowness (S, in s/km). Here, "a" and "R%" represent Gaussian width factor (used for estimating RF) and agreement (in %) between observed and inverted RFs, respectively. Correlation between observed and inverted dispersion curves of (b) Rayleigh waves. (c) Inverted shear velocity models showing the main Himalayan thrust (MHT), Moho (M) and LAB (L) depth estimates in km. Different colours represent different Vs models used for the joint inversion. The initial shear velocity model is shown by a thick red dotted line, while the final shear velocity model is shown by a thick blue line and (d) A plot showing zoomed portion of the crustal part from the inverted Vs models.

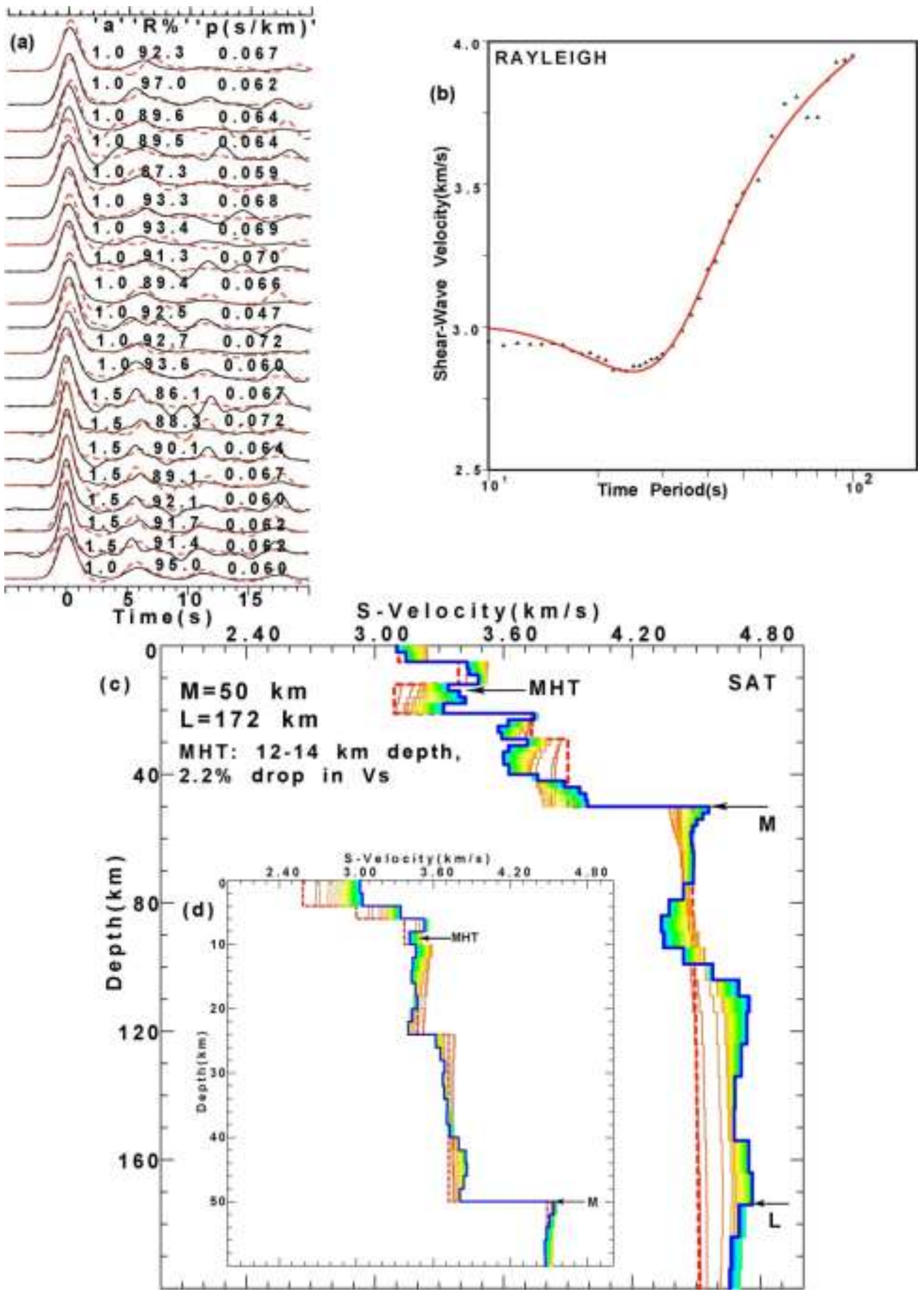


Fig S50: Results of joint inversion of P-RFs and fundamental mode surface wave group velocity dispersion (SWD) data at SAT station, (a) showing good agreement between observed (black line) and inverted (red line) radial RFs with a=1.0, 1.5 and 2.0, for different horizontal slowness (S, in s/km). Here, "a" and "R%" represent Gaussian width factor (used for estimating RF) and agreement (in %) between observed and inverted RFs, respectively. Correlation between observed and inverted dispersion curves of (b) Rayleigh waves. (c) Inverted shear velocity models showing the main Himalayan thrust (MHT), Moho (M) and LAB (L) depth estimates in km. Different colours represent different Vs models used for the joint inversion. The initial shear velocity model is shown by a thick red dotted line, while the final shear velocity model is shown by a thick blue line and (d) A plot showing zoomed portion of the crustal part from the inverted Vs models.

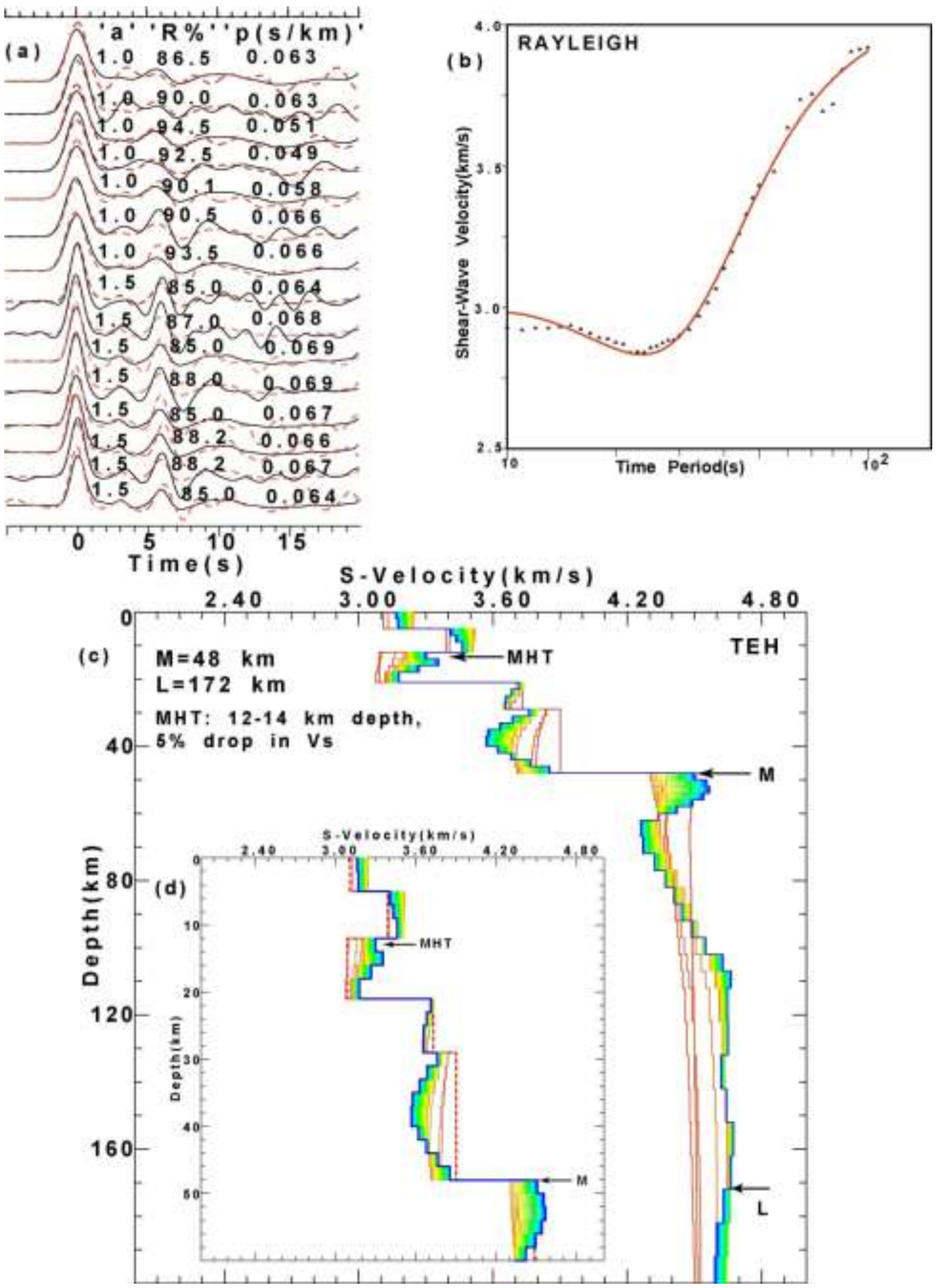


Fig S51: Results of joint inversion of P-RFs and fundamental mode surface wave group velocity dispersion (SWD) data at TEH station, (a) showing good agreement between observed (black line) and inverted (red line) radial RFs with a=1.0, 1.5 and 2.0, for different horizontal slowness (S, in s/km). Here, "a" and "R%" represent Gaussian width factor (used for estimating RF) and agreement (in %) between observed and inverted RFs, respectively. Correlation between observed and inverted dispersion curves of (b) Rayleigh waves. (c) Inverted shear velocity models showing the main Himalayan thrust (MHT), Moho (M) and LAB (L) depth estimates in km. Different colours represent different Vs models used for the joint inversion. The initial shear velocity model is shown by a thick red dotted line, while the final shear velocity model is shown by a thick blue line and (d) A plot showing zoomed portion of the crustal part from the inverted Vs models.

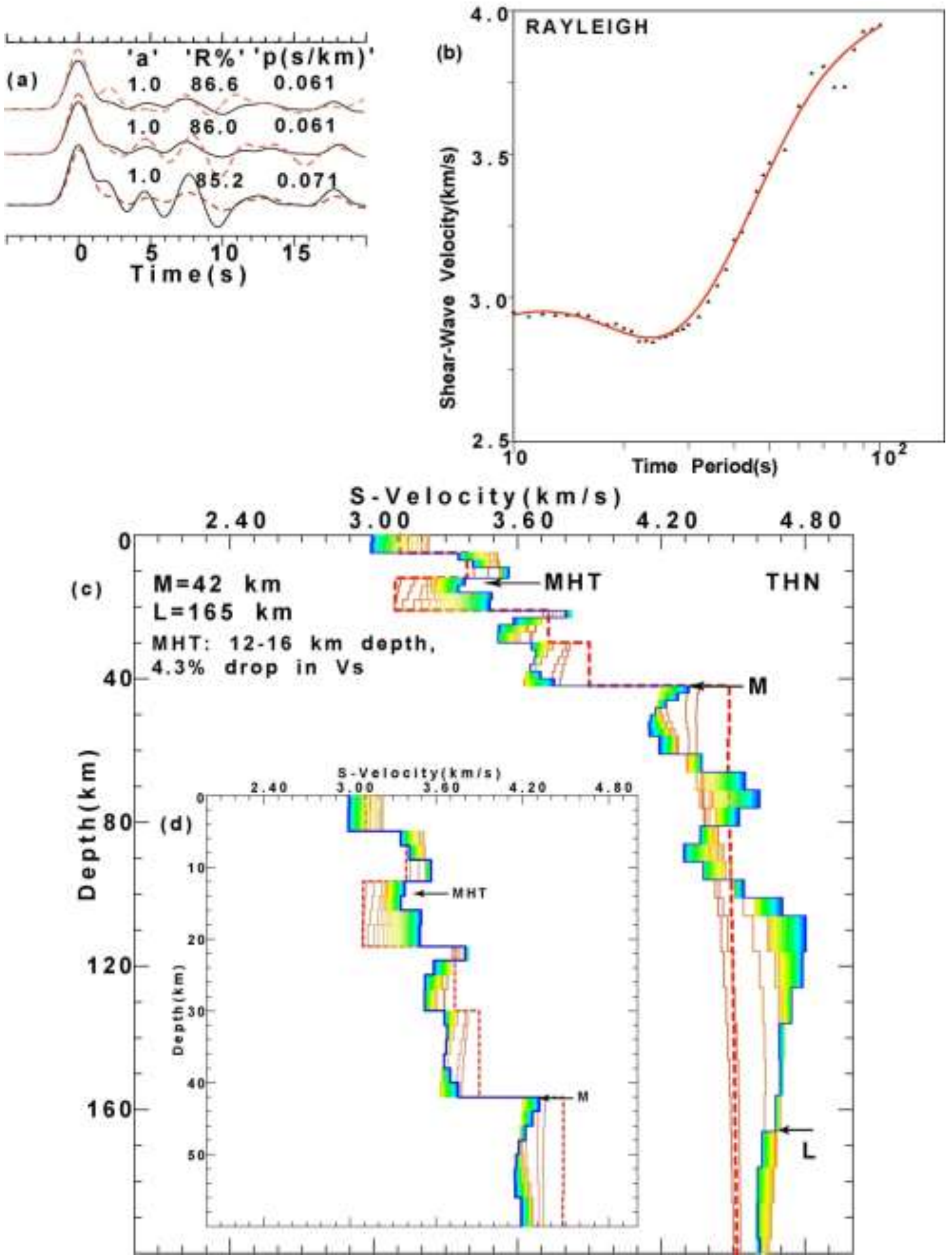


Fig S52: Results of joint inversion of P-RFs and fundamental mode surface wave group velocity dispersion (SWD) data at THN station, (a) showing good agreement between observed (black line) and inverted (red line) radial RFs with a=1.0, 1.5 and 2.0, for different horizontal slowness (S, in s/km). Here, "a" and "R%" represent Gaussian width factor (used for estimating RF) and agreement (in %) between observed and inverted RFs, respectively. Correlation between observed and inverted dispersion curves of (b) Rayleigh waves. (c) Inverted shear velocity models showing the main Himalayan thrust (MHT), Moho (M) and LAB (L) depth estimates in km. Different colours represent different Vs models used for the joint inversion. The initial shear velocity model is shown by a thick red dotted line, while the final shear velocity model is shown by a thick blue line and (d) A plot showing zoomed portion of the crustal part from the inverted Vs models.

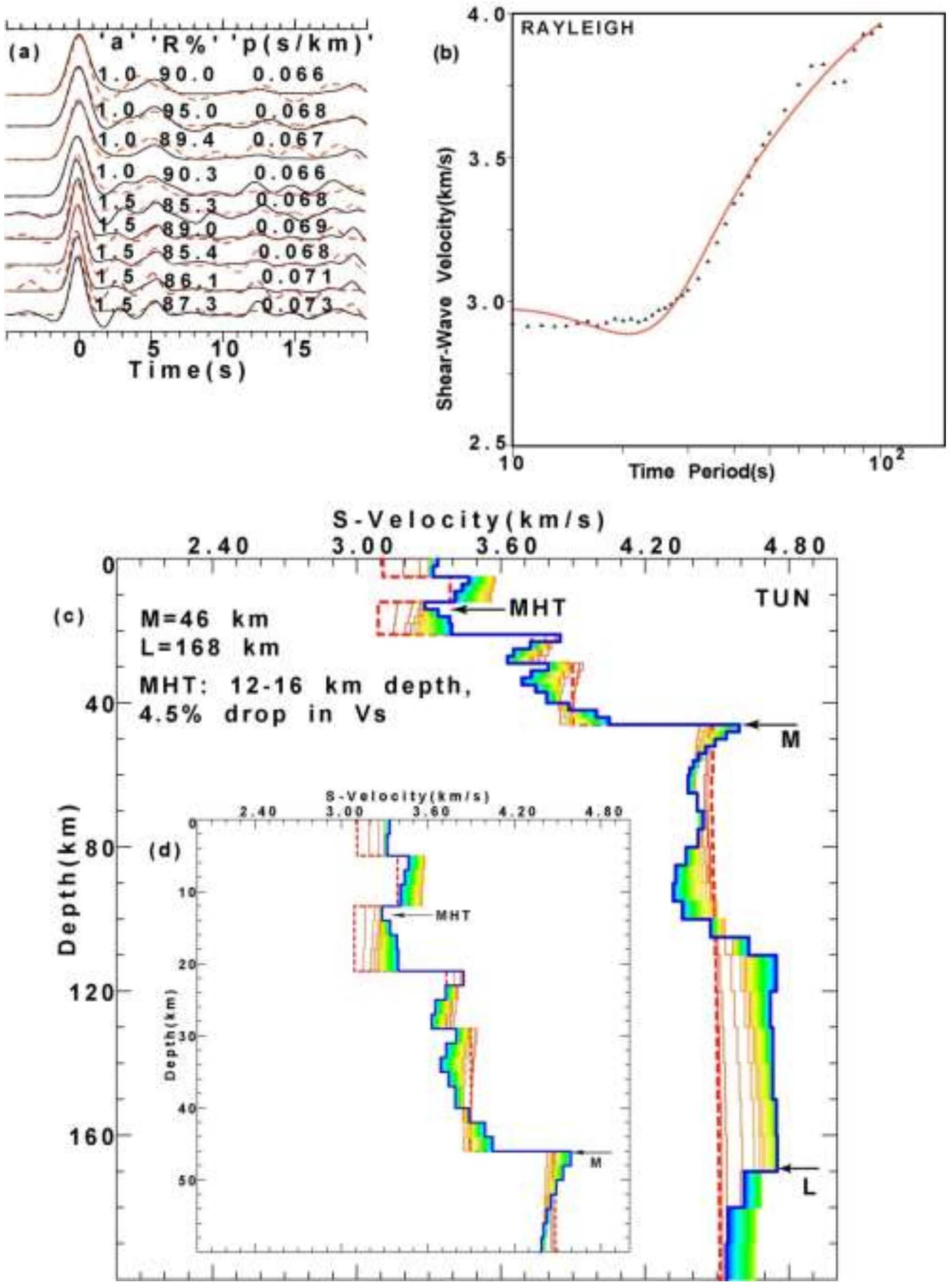


Fig S53: Results of joint inversion of P-RFs and fundamental mode surface wave group velocity dispersion (SWD) data at TUN station, (a) showing good agreement between observed (black line) and inverted (red line) radial RFs with a=1.0, 1.5 and 2.0, for different horizontal slowness (S, in s/km). Here, "a" and "R%" represent Gaussian width factor (used for estimating RF) and agreement (in %) between observed and inverted RFs, respectively. Correlation between observed and inverted dispersion curves of (b) Rayleigh waves. (c) Inverted shear velocity models showing the main Himalayan thrust (MHT), Moho (M) and LAB (L) depth estimates in km. Different colours represent different Vs models used for the joint inversion. The initial shear velocity model is shown by a thick red dotted line, while the final shear velocity model is shown by a thick blue line and (d) A plot showing zoomed portion of the crustal part from the inverted Vs models.

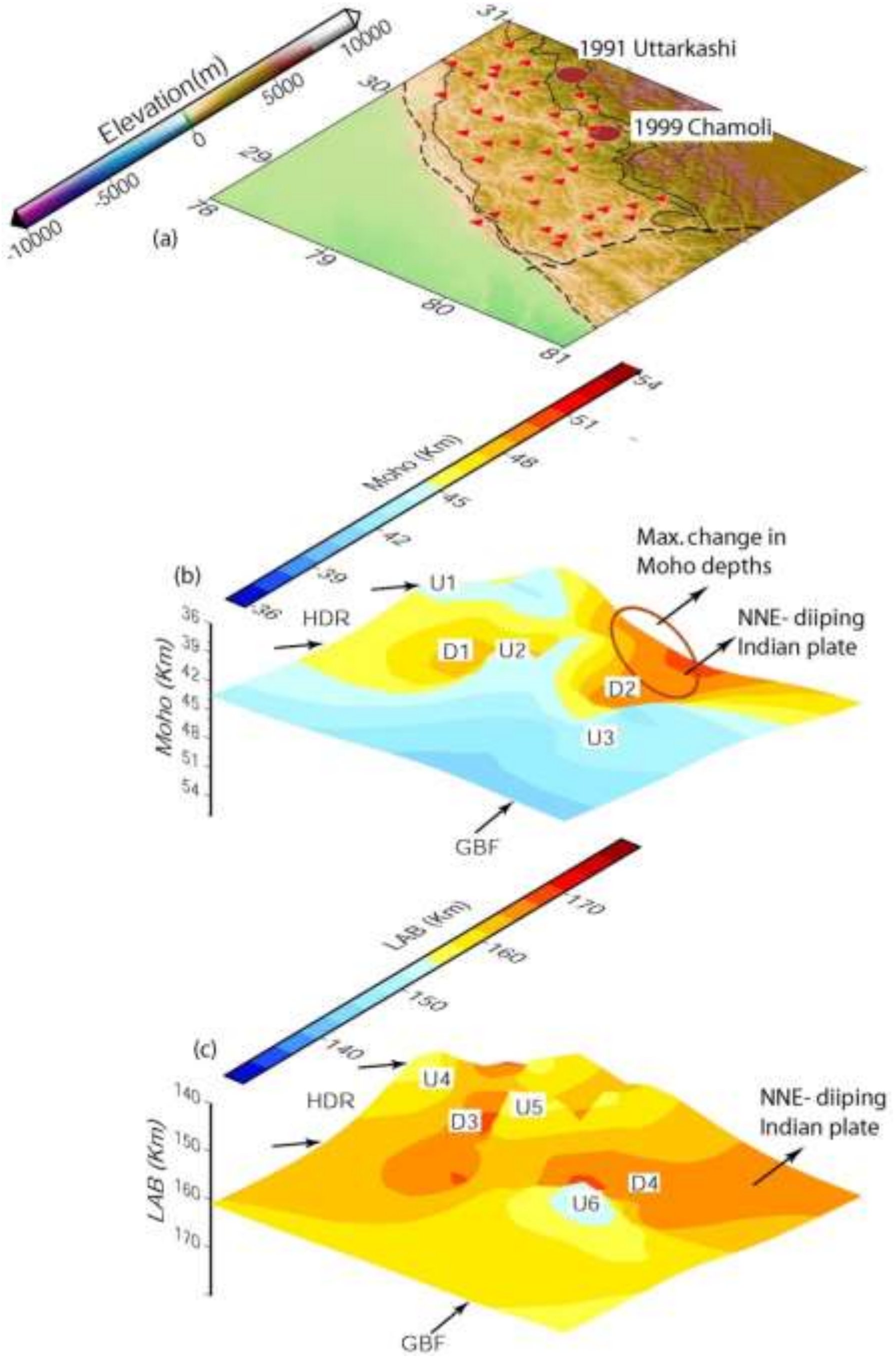


Figure S54: (a) Elevation (in m) map along with seismic stations (marked by red filled triangles). While two large red filled circles represent the locations of the 1991 $M_w$6.6 Uttarkashi and 1999 $M_w$6.4 Chamoli earthquakes. 3-D surface plots of modelled (b) Moho (km) and (c) Lithospheric thickness (in km). The zone with the maximum changes in Moho depths are marked by a black elliptical area in Fig. 7b while "N- dipping Indian plate" marks the inferred north-easterly subducted Indian Moho and Lithosphere (Figs. 7b-c). Marked crustal/lithospheric thinning zones are shown by U1, U2, U3, U4, U5, and U6 while marked crustal/lithospheric thickening zones are marked by D1, D2, D3 and D4. Black arrows show the inferred NE-ward extension of the DHR and GBF.

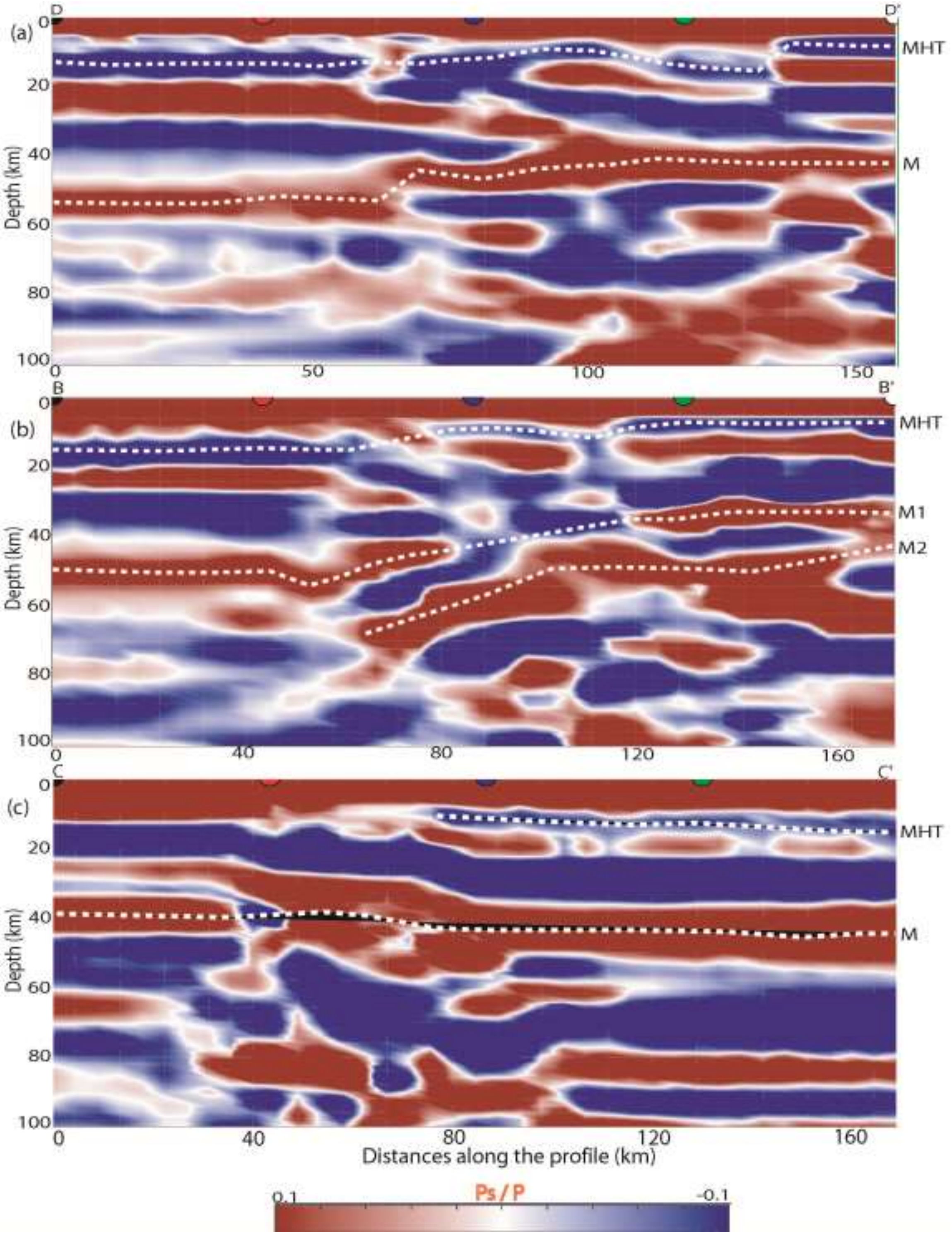


Figure S55: CCP stacking of PRFs using 1-D IASP91 velocity model along three profiles, whose locations are shown in Fig. 1a. Dotted lines show north dipping Main Himalayan Thrust (MHT) and Moho (M) below the Lesser Himalaya in the Uttarakhand Himalaya (a) DD' profile, (b) BB' profile and (c) CC' profiles. The locations of these profiles are shown in Fig.2a. Double Moho structure, which is shown by white dotted lines and marked by M1 and M2, is also mapped below the epicentral zone of the 1999 Chamoli earthquake along the BB' profile.

Table S1: Modelled MHT, Moho and LAB depths at 45 broadband stations in the Uttarakhand Himalaya

| S. Nos. | Station | Latitude ($^oN$) | Longitude ($^oE$) | MHT thick (km) | Drop in Vs at MHT(%) | Moho (km) | Increase in Vs(km/s) | LAB Thick (km) | Drop in Vs at LAB(%) |
|---|---|---|---|---|---|---|---|---|---|
| 1 | ALM | 29.59 | 79.65 | 2 | 3 | 44 | 0.52 | 154 | 1.7 |
| 2 | AUGM | 30.39 | 79.02 | 2 | 3.4 | 45 | 0.60 | 155 | 1.8 |
| 3 | BAR | 30.81 | 78.21 | 2 | 3.4 | 49 | 0.61 | 166 | 2.2 |
| 4 | BDKD | 30.58 | 78.65 | 2 | 1.7 | 42 | 0.58 | 152 | 2.34 |
| 5 | BNG | 29.78 | 80.05 | 4 | 5.7 | 46 | 0.60 | 156 | 2.34 |
| 6 | BTL | 29.36 | 79.55 | 4 | 3.1 | 39 | 0.50 | 178 | 2.2 |
| 7 | CHA | 30.69 | 77.87 | 4 | 4.2 | 49 | 0.55 | 168 | 1.7 |
| 8 | CHIN | 30.58 | 78.33 | 2 | 2 | 46 | 0.55 | 165 | 2.0 |
| 9 | CHPT | 29.33 | 80.11 | 2 | 3.4 | 42 | 0.55 | 161 | 2.2 |
| 10 | DDHT | 29.80 | 80.25 | 2 | 3.2 | 42 | 0.55 | 162 | 2.5 |
| 11 | DEV | 30.14 | 78.61 | 9 | 4.9 | 47 | 0.58 | 166 | 2.0 |
| 12 | DHA | 30.43 | 78.24 | 2 | 4.2 | 45 | 0.54 | 164 | 2.0 |
| 13 | DHRL | 29.85 | 80.54 | 4 | 4.2 | 46 | 0.60 | 136 | 1.0 |
| 14 | DRT | 29.77 | 79.42 | 4 | 3.2 | 44 | 0.56 | 164 | 2.0 |
| 15 | DUM | 29.75 | 79.02 | 2 | 2 | 45 | 0.55 | 164 | 2.1 |
| 16 | GAI | 30.05 | 79.29 | 2 | 3.4 | 45 | 0.56 | 164 | 3.2 |
| 17 | GDM | 30.00 | 79.56 | 2 | 3 | 46 | 0.55 | 165 | 1.9 |
| 18 | GGHT | 29.66 | 80.04 | 2 | 4.5 | 43 | 0.54 | 152 | 2.6 |
| 19 | GHAT | 30.26 | 79.45 | 4 | 3.4 | 50 | 0.57 | 170 | 1.7 |
| 20 | GHAN | 30.43 | 78.64 | 2 | 2 | 47 | 0.60 | 176 | 1.7 |
| 21 | GOPE | 30.41 | 79.32 | 9 | 4.8 | 45 | 0.53 | 165 | 3.0 |
| 22 | HLD | 29.23 | 79.53 | 2 | 2.9 | 43 | 0.52 | 160 | 2.5 |
| 23 | JOS | 30.72 | 78.44 | 2 | 2.9 | 44 | 0.52 | 162 | 2.34 |
| 24 | KAL | 30.52 | 77.84 | 2 | 2.9 | 48 | 0.57 | 172 | 1.0 |
| 25 | KAN | 30.51 | 78.38 | 9 | 5 | 43 | 0.60 | 148 | 2.3 |
| 26 | KAPG | 30.27 | 79.22 | 2 | 2 | 43 | 0.53 | 166 | 2.3 |
| 27 | KSN | 29.85 | 79.60 | 4 | 3.7 | 43 | 0.53 | 166 | 2.3 |
| 28 | LAN | 29.84 | 78.68 | 4 | 2.9 | 48 | 0.60 | 172 | 1.0 |
| 29 | LOGH | 29.40 | 80.09 | 2 | 2.9 | 43 | 0.57 | 158 | 2.3 |
| 30 | MNRI | 30.07 | 80.24 | 2 | 4.8 | 44 | 0.62 | 166 | 2.0 |
| 31 | MUS | 30.46 | 78.07 | 2 | 2.2 | 46 | 0.56 | 158 | 1.0 |
| 32 | NACH | 29.91 | 80.17 | 2 | 3.1 | 44 | 0.56 | 158 | 2.7 |
| 33 | NAR | 30.16 | 78.29 | 4 | 2.9 | 45 | 0.53 | 158 | 2.3 |
| 34 | NBR | 30.15 | 79.38 | 9 | 4.8 | 46 | 0.54 | 170 | 2.1 |
| 35 | PATI | 29.41 | 79.93 | 9 | 4.5 | 42 | 0.52 | 156 | 2.0 |
| 36 | PAUR | 30.16 | 78.77 | 4 | 5 | 47 | 0.57 | 150 | 2.1 |
| 37 | PITH | 29.552 | 80.234 | ??? | ??? | 45 | 0.60 | 148 | 1.9 |
| 38 | SAT | 29.92 | 78.71 | 2 | 2.2 | 50 | 0.56 | 172 | 1.5 |
| 39 | THE | 30.37 | 78.43 | 2 | 5 | 48 | 0.65 | 172 | 1.1 |
| 40 | TRNR | 30.64 | 78.98 | 9 | 5 | 48 | 0.56 | 170 | 2.0 |
| 41 | TUN | 30.93 | 77.85 | 4 | 4.5 | 46 | 0.55 | 168 | 2.8 |
| 42 | POKH | 29.92 | 78.92 | 2 | 2 | 43 | 0.52 | 158 | 3.2 |
| 43 | RANS | 30.59 | 79.14 | 4 | 4.2 | 50 | 0.59 | 154 | 2.0 |
| 44 | RPG | 30.28 | 78.979 | 4 | 5 | 50 | 0.59 | 172 | 1.3 |
| 45 | THN | 30.02 | 79.05 | 4 | 4.3 | 42 | 0.56 | 165 | 1.4 |
